# The Genome Organization of 5S rRNA Genes in the Model Organism *Tribolium castaneum* and Its Sibling Species *Tribolium freemani*

**DOI:** 10.3390/genes15060776

**Published:** 2024-06-13

**Authors:** Marin Volarić, Evelin Despot-Slade, Damira Veseljak, Martina Pavlek, Tanja Vojvoda Zeljko, Brankica Mravinac, Nevenka Meštrović

**Affiliations:** Ruđer Bošković Institute, Bijenička Cesta 54, 10000 Zagreb, Croatia; mvolaric@irb.hr (M.V.); evelin.despot.slade@irb.hr (E.D.-S.); damira.veseljak@irb.hr (D.V.); martina.pavlek@irb.hr (M.P.); tanja.vojvoda@irb.hr (T.V.Z.); brankica.mravinac@irb.hr (B.M.)

**Keywords:** 5S rRNA, genome organization, *Tribolium*, sibling species, long-read assemblies

## Abstract

5S ribosomal DNAs (rDNAs) are arranged in tandem and are often under-represented in genome assemblies. In the present study, we performed a global and in-depth analysis of the 5S rDNAs in the model insect *Tribolium castaneum* and its closely related species *Tribolium freemani*. To accomplish this goal, we used our recently published genome assemblies based on Nanopore and PacBio long-read sequencing. Although these closely related species share the 5S rRNA gene sequence with high homology, they show a different organization of the 5S rDNA locus. Analysis of 5S rDNA arrays in *T. castaneum* revealed a typical tandemly repeated organization characterized by repeat units consisting of the 121 bp long 5S rRNA gene and the 71 bp long nontranscribed spacer (NTS). In contrast, *T. freemani* showed a much more complex organization of 5S rDNA arrays characterized by two patterns. The first is based on the association of 5S rRNA gene with arrays of a satellite DNA, representing the NTS sequence of the 5S rDNA genes in *T. freemani*. The second, more complex type is characterized by a somewhat less frequent occurrence of the 5S rRNA gene and its association with longer satellite DNA arrays that are regularly interrupted by Jockey-like retrotransposons. This organization, in which the ribosomal gene is associated with two completely different repetitive elements such as satellite DNAs and retrotransposons, suggests that the 5S rRNA gene, regardless of its crucial function in the genome, could be a subject of extremely dynamic genomic rearrangements.

## 1. Introduction

The ribosomal genes (rDNA), which code for 18S, 5.8S, 28S, and 5S ribosomal RNA (rRNA) are directly involved in the biogenesis and function of ribosomes, which are responsible for protein synthesis. Clusters of the 18S, 5.8S, and 28S rRNA genes linked by intergenic spacers form the nucleolus organizer region (NOR). They are typically encoded in a co-transcribed operon, known as the 45S in humans. In contrast, the copies of the 5S rRNA gene are not associated with the NOR and may be clustered at one or more chromosomal sites outside the NOR. The 5S rDNA locus is typically arranged in tandem arrays with repeat units composed of an approximately 120 bp long gene and a nontranscribed spacer (NTS). Dispersed organization of 5S rDNA loci is less common. Data from a broad range of higher eukaryotes have shown that 5S rDNA genes are highly conserved even among unrelated taxa, whereas NTS regions evolve more rapidly, both in length and in nucleotide sequence (reviewed in [1]). The 5S rRNA gene contains a three-part RNA polymerase III promoter consisting of the three motifs: Box-A, Internal Element (IE) and Box-C, which appear to be conserved among species [2]. It has been proposed that 5S rRNA evolution results from a mixed process of concerted and birth-and-death evolution [3]. The model of concerted evolution, which is common for tandemly repeated sequences such as 5S rRNA genes, states that repeated units undergo homogenization, making the multiple copies more homogenous in sequence than would be expected given the mutation frequencies. In contrast, the birth-and-death model of evolution implies repeated 5S rDNA amplification at different genomic locations. Some of the duplicated genes could be preserved in the genome for a long time, while others could be deleted. Over time, sequences of different members of the same gene family can become very different within species. Consequently, high levels of intragenomic repeat variation are expected in 5S rDNA repeats that evolve through the birth-and-death process. 

Another interesting feature of 5S rDNA evolution is that although 5S rDNAs are typically organized in monotonous tandems associated with NTS, they can also be found in combination with other repetitive elements. For example, single copies of the 5S rDNA are found inserted into 45S rDNA intergenic spacers in the genomes of some fishes [4]. Several studies on the organization and molecular evolution of 5S rDNA have reported association of 5S rRNA genes with satellite DNAs (satDNAs) [5,6]. SatDNAs are the most abundant and rapidly evolving noncoding DNAs of all eukaryotic genomes. SatDNAs are characterized by monomer sequences tandemly arranged into long arrays. Previous studies on many animal and plant species indicate preferential localization of satDNAs in the (peri)centromeric heterochromatin [7]. However, some studies have revealed examples where satDNAs also spread to euchromatic regions [8]. 5S rRNA genes could be also linked with transposable elements (TEs). TEs represent another abundant fraction of repetitive DNAs in complex genomes, and in contrast to tandemly organized satDNA they are dispersed throughout the genome. TEs occur in a wide variety of structures and sequences. 5S rRNA genes in zebrafish are insertion targets for a specific family of retrotransposons called Mutsu [9,10]. The DNA sequence of Mutsu retrotransposons is approximately 5500 bp long and consists of two open reading frames (ORFs) flanked by untranslated regions (UTRs). Recent evidence has shown the occurrence of 5S-related nonautonomous retrotransposons, so-called Cassandras, in plants [11]. Cassandras harbor highly conserved 5S rDNA-related sequences within their long terminal repeats. 

Next-generation sequencing (NGS) techniques have been intensively used to assemble genomes across species in the past two decades. However, the genome assemblies produced exclusively with NGS reads are usually poor in continuity due to the presence of repetitive sequences, especially in those regions consisting of tandemly repeated sequences such as 5S rDNA loci. For that reason, these assemblies fail to resolve the organization and evolution of 5S rDNA clusters on the genome-wide scale [12]. 

The representative species of Coleoptera is the red flour beetle, *T. castaneum*. *T. castaneum* has become one of the most important models in the field of evolution, physiology, and development of insects [13]. *T. freemani*, is a sibling species of *T. castaneum*. Although the hybrid offspring are sterile [14], the fact that the two species can hybridize speaks in favor of their genetic similarity. The two siblings are also excellent models for the study of repetitive DNA sequences, as their genomes are rich in repeats. Our extra efforts have been made recently to improve the continuity of the *T. castaneum* assembly, by filling the gaps and elongating the repetitive regions, using long-read Nanopore sequencing [15]. In this way, with our new genome assembly, called TcasONT, we have improved the previous reference assembly Tcas5.2 [16] by extending it by 45 Mb. By analyzing tandemly organized satDNAs, we showed that TcasONT is superior to Tcas5.2, especially with regard to repetitive sequences [15]. We also generated the high-quality genome assembly of *T. freemani* by using highly accurate PacBio HiFi sequencing [17]. 

In this study, we analyzed all putative 5S rRNA genes of the model organism *T. castaneum* and its sibling species *T. freemani* to uncover their genome organization and evolution. For this purpose, we used our recently published assemblies of *T. castaneum* and *T. freemani*, which were generated using long-read sequencing technology and were found to be rich in the repetitive fraction of the genome [15,17].

## 2. Materials and Methods

### 2.1. Insect Material

Laboratory cultures of the red flour beetle *T. castaneum*, highly cultured strain Georgia 2 (GA2), were routinely reared in whole wheat flour with the addition of whole rye flour and oats under conditions favoring faster multiplication (32 °C and 70% relative humidity) in the dark. The initial stock of the flour beetle *T. freemani* was obtained from USDA-ARS (Manhattan, KS, USA) in 2015 and maintained as a laboratory culture. The insects were reared in the whole wheat flour at 27 °C and 70% humidity in the dark, being sub-cultured every four weeks.

### 2.2. Data Resources

The reference genome assembly of *T. castaneum* Tcas5.2 (GCA_000002335.3) can be found at the European Nucleotide Archive 758 (ENA) under the BioProject accession PRJNA15718 [16]. The *T. castaneum* genome assembly TcasONT (GCA_950066185) can be found under PRJEB61413. The genome annotation used in this study is available in FigShare at https://doi.org/10.6084/m9.figshare.22683325.v1 (accessed on 15 March 2024). The *T. freemani* genome assembly Tfree1.0 (GCA_939628115.1) can be accessed at the European Nucleotide Archive (ENA) under the BioProject accession PRJEB52307 [17]. The annotated data used in this study are openly available in FigShare at https://doi.org/10.6084/m9.figshare.19682400.v1 (accessed on 15 March 2024).

### 2.3. 5S rDNA Analyses

Annotation and discovery of the 5S rRNA genes within the *T. castaneum* and *T. freemani* assemblies were performed using the NCBI’s BLAST algorithm and the interface to the R programming language package metablastr [18]. As a query, the 5S rRNA gene from *Drosophila melanogaster* (GenBank entry X58539.1) was used, and the repeat was discovered if the BLAST search resulted in a query coverage and percent identity > 70% for the sequence. RepeatMasker is a widely used tool for finding and masking repetitive elements within a given target sequence [19]. RepeatMasker was used previously in order to obtain the GFF/GTF formatted data with the position and orientation of different repetitive elements in the genomes, and their features in relation to 5S rDNA organization were examined. Repetitive and transposable elements were identified using the RepeatMasker program on the Galaxy platform (usegalaxy.org) with the RepBase database and the “Hexapoda” species listing for clade-specific repeats. All masking was performed on the Galaxy server using the RepBase RELEASE 20181026 and RepeatMasker (4.0.9_p2). All of the repeat content and sequence analyses were performed on the GFF files present in the annotation data. We used MAFFT [20] to create multiple sequence alignments of 5S rRNA genes, NTS regions, and Jockey-like elements. Initial sequence manipulations, visualization, and similarity values were obtained using Geneious 2023.2.1 software and BioEdit v.7.0.5. Secondary rRNA structures were created in Geneious using RNAfold [21] with the DNA Matthews 2004 energy model.

### 2.4. PCR Amplification, Cloning, and Probe Preparation

Genomic DNAs were isolated from adults using standard phenol–chloroform extraction. The 5S rRNA genes were amplified with specific primers (5′ CCATACCACGTTGTAAAGCAC 3′ and 5′ TCGTCCGATCACTGAAGTTAAGC 3′) derived from consensus sequences. PCR was carried out using reaction buffer, 1.5 mM MgCl_2_, 0.2 mM dNTPs, 0.5 U GoTaq DNA polymerase (Promega, Madison, WI, USA), 0.4 mM of each primer, and 20 ng of genomic DNA. Amplification was performed under the following conditions: 2 min initial denaturation at 94 °C, followed by 30 cycles of: 95 °C for 30 s, 55 °C for 30 s, and 72 °C for 1 min. Final extension was completed at 72 °C for 10 min. PCR products were purified using the QIAquick PCR Purification Kit (Qiagen, Venlo, The Netherlands). PCR products were ligated in a pGEMT-Easy vector (Promega) and transformed in *Escherichia coli* DH5α-competent cells (Invitrogen, Waltham, MA, USA). Recombinant clones were sequenced by the Macrogen Europe Laboratory (Amsterdam, The Netherlands). DNA probes for fluorescence in situ hybridization (FISH) were generated from cloned 5S rDNA fragments. DNA probes were labeled with biotin-16-dUTP by using the Nick Translation Kit (Roche, Basel, Switzerland).

### 2.5. Slide Preparation and Fluorescence In Situ Hybridization (FISH)

The localization of the 5S rDNA on the *T. castaneum* and *T. freemani* chromosomes was performed using FISH. Chromosome spreads were prepared from male gonads of pupae using the squash method as previously described [22]. FISH with biotin-labeled 5S rDNA probes obtained using nick translation was performed for 18 h at 37 °C in a buffer containing 60% formamide, 2× SSC, 10% dextran sulfate, 20 mM Na_2_HPO_4_, and 10 ng/μL of biotin-labeled probe. Post-hybridization washes were performed in 50% formamide/2× SSC at 37 °C. Biotin-labeled probes were detected with fluorescein-avidin D and biotinylated anti-avidin D (Vector Laboratories, Newark, CA, USA) by signal amplification across three layers of fluorophore conjugates and antibodies using the following dilutions: 1:500 fluorescein-avidin D, 1:100 biotinylated anti-avidin D, and 1:2000 fluorescein-avidin D. Finally, the slides were counterstained in 4′,6-diamidino-2-phenylindole (DAPI) solution for 15 min, air-dried, and embedded in Mowiol 4–88 mounting medium (Sigma-Aldrich, St. Louis, MO, USA). Visualization of the slides and image acquisition were performed using a Leica TCS SP8 X confocal laser scanning microscope (Leica Microsystems, Wetzlar, Germany) equipped with an HC PL APO CS2 63×/1.40 oil objective, a 405 nm diode laser, and a supercontinuum excitation laser (Leica Microsystems, Wetzlar, Germany). Images were captured separately for each fluorochrome (DAPI or fluorescein) and processed with ImageJ [23] and Adobe Photoshop v.21.0.3. (Adobe Systems, San Jose, CA, USA).

## 3. Results

To identify 5S rDNA genes in *T. castaneum*, a BLAST search was performed on our recently provided TcasONT genome assembly using *D. melanogaster* 5S rDNA as the query. Two arrays of 5S rRNA genes were found on chromosome LG2 and two on chromosome LG3 (Table 1). A total of 307 5S rRNA genes were mapped and extracted. They were organized as tandemly repeated arrays containing between 6 and 201 5S rRNA genes. Only four dispersed copies were found on other chromosomes. For comparison, only five copies of 5S rRNA genes were found in the previous genome assembly Tcas5.2 using the same approach. In addition, to identify 5S rDNA genes in the sibling species *T. freemani* we used our recently generated long-read genome assembly based on PacBio HiFi technology [17]. Similar to *T. castaneum*, four arrays of 5S rRNA genes were found distributed on chromosomes fLG2 and fLG3 (Table 1).

A total of 136 5S rRNA genes were found in *T. freemani*, which is more than twice fewer than in the *T. castaneum* genome. Sequence analyses showed that the 5S rRNA genes are 121 bp in length. However, detailed examination of the arrays revealed that there is a subset of 5S rDNA copies of shorter length in both species, and we hypothesize that these shorter variants are pseudogenes. In *T. castaneum*, there are about 20 5S rDNA copies with a deletion of 30 bases at the end of the gene (Supplementary Figure S1A). In *T. freemani*, as many as 32 5S rDNA copies among 113 have a 30 bp deletion at the beginning of the gene (Supplementary Figure S1B). Apart from these sequence deletions, the intraspecific sequence similarity is very high: 99.4% in both species (Supplementary Figure S1).

Considering that repetitiveness of 5S rRNA clusters can lead to assembly collapse, which can result in the number of units in a genome assembly being lower than in the real genome, we performed additional analyses of the mapping of both 5S rRNA arrays and repetitive content in surrounding regions to raw data cosmpared to assemblies (Supplementary Figure S2A,B). The results confirmed the previously shown results [15,17] that both genomes provide an excellent platform for the analysis of repetitive sequences, especially problematic tandem repeats.

**Figure 1:**
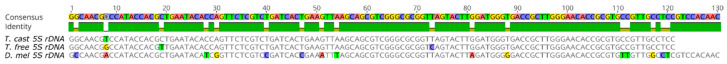
Alignment of consensus sequences of 5S rRNA gene in *T. castaneum* and *T. freemani*. *T. castaneum* and *T. freemani* consensuses were generated from alignments (Supplementary Figure S1). 5S rRNA gene from *Drosophila melanogaster* was taken from NCBI GenBank database (accession number NR_001870.2).

The species–specific consensus sequences were generated using sequence alignments without truncated copies. The sequence comparison between the consensus sequence of *T. castaneum* and *T. freemani* also shows a high interspecific nucleotide similarity with only three nucleotide changes between the species (Figure 1).

**Figure 2:**
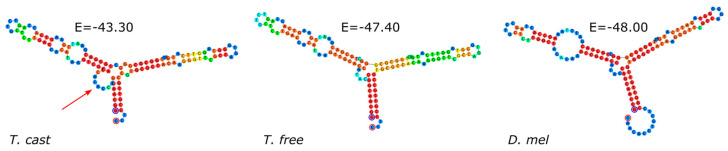
Secondary structure of 5S rDNA in *T. castaneum*, *T. freemani* and *D. melanogaster* using RNAfold [21] with DNA Matthews 2004 energy model with folding energies represented above the structure in kcal/mol. The arrow indicates changes in internal loops between *T. castaneum* and *T. freemani*.

As expected, *T. freemani* differs more from the 5S rDNA of *D. melanogaster* showing 15 nucleotide changes compared to *T. castaneum*. The predicted secondary structures show a high similarity between *T. castaneum* and *T. freemani*, matching also that of *D. melanogaster* (Figure 2). However, the three substitutions between *T. freemani* and *T. castaneum* affect the secondary structure and change the internal loop structure (marked by an arrow in Figure 2).

**Figure 3:**
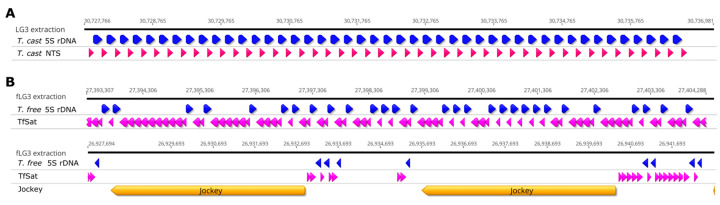
Organization of 5S rRNA gene (blue) and NTS sequences in *T. castaneum* (**A**) and *T. freemani* (**B**). *T. freemani* has two types of organization: one that comes with satDNA sequence TfSat (**above**) and the other composed of satDNA TfSat and Jockey retrotransposon element (**below**).

Analysis of the organization of the 5S rDNA arrays in *T. castaneum* revealed a typical tandemly repeated organization characterized by repeated units consisting of a 5S rDNA gene of 121 bp and a 71 bp long NTS (Figure 3A). Analyses of the distribution of shorter 5S rRNA variants (pseudogenes) show their random occurrence among the complete genes in the long array on LG2 chromosomes (Supplementary Figure S3). The NTS alignment in *T. castaneum* shows conservation of sequence length but slightly lower intraspecific similarity (94.1%) (Supplementary Figure S4A) than that of the 5S rRNA genes.

The analysis of the 5S rRNA gene in *T. freemani* proved to be much more complex and revealed two organizational patterns. The first is characterized by the association of the 5S rRNA gene with satDNA (called TfSat; publication in preparation). In this organization, the 5S rRNA gene alternates with tandemly repeated arrays composed of one to ten TfSat monomers (Figure 3B). This organization suggests that TfSat satDNA represents an NTS sequence in *T*. *freemani* 5S rDNA arrays (Figure 3B). Alignment of TfSat monomers revealed two groups; one group has a longer monomer of 122 bp while the other group is based on a 71 bp long monomer (Supplementary Figure S4B). Sequence similarity among these NTS sequences is 72.8%. TfSat satDNA is found in the *T. freemani* genome exclusively in association with the 5S rRNA gene, and it is not present in the *T. castaneum* genome assembly in any form.

**Figure 4:**
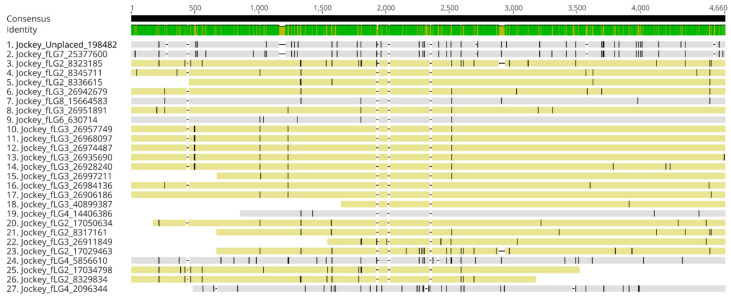
Alignment of Jockey elements in *T. freemani* with those associated with 5S rRNA genes highlighted (in yellow). The remaining Jockey elements (in gray) are dispersed throughout the genome.

The other, more complex type of 5S rDNA organization in *T. freemani* is characterized by a somewhat less frequent occurrence of 5S rRNA genes and their association with longer satDNA arrays (Figure 3B). In addition, the repeats of 5S rDNA gene copies associated with TfSat satDNA arrays were regularly interrupted by DNA segments of ~5 kb. On closer inspection, these segments were found to be the Jockey-like non-long terminal repeat (non-LTR) retrotransposons, which belong to the group of Long Interspersed Nuclear Elements (LINEs). Jockey-like retrotransposons found in 5S rDNA arrays show the highest similarity in the parts that include nucleic-acid-binding protein with other Jockey elements from RepBase database (Supplementary Figure S5). To investigate the relationship between Jockey-like retrotransposons associated with 5S rRNA genes and those distributed throughout the genome, all Jockey-like retrotransposons were extracted and aligned (Figure 4).

The results showed that Jockey-like retrotransposons were mostly associated with 5S rRNA genes and exhibit high sequence similarity and relative conservation in length. Jockey-like gene copies located outside the 5S rDNA arrays were slightly more divergent. Analysis of Jockey-like elements in *T. castaneum* also showed the presence of these elements in its genome, but they are not associated with 5S rDNA genes, and at the same time they are far more divergent in sequence and length than in *T. freemani* (Supplementary Figure S6). When analyzing the distribution of shorter 5S rRNA variants, the results showed that pseudogenes are exclusively associated with this complex organization of 5S rRNA genes containing the Jockey-like element (Supplementary Figure S3B).

**Figure 5:**
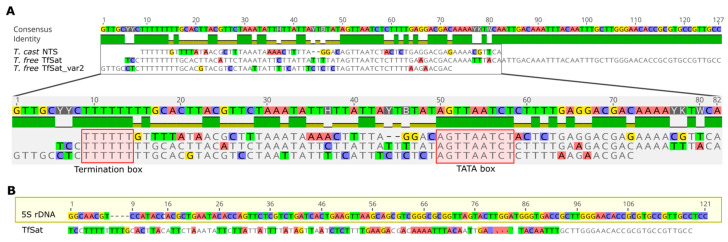
(**A**) Alignment of NTS consensus sequences in *T. castaneum* and *T. freemani* with marked regions of termination and TATA box in red. Consensus sequences were generated based on alignments (Supplementary Figure S3). TfSat_var2 represents a consensus sequence of a shorter variant of NTS in *T. freemani*. (**B**) Alignment of 5S rRNA gene and longer variant of *T. freemani* NTS (TfSat).

We also wanted to analyze the NTS sequences between these sibling species to find putative conserved traits, because the NTS sequences are often highly polymorphic and differ in sequence and length even between closely related species. For this reason, we compared the consensuses of *T. castaneum* and *T. freemani* NTS sequences and found a relatively high similarity, especially between the NTS of *T. castaneum* and the shorter variant of *T. freemani* (Figure 5A). All three NTSs show similarity in a ~70 bp sequence stretch. They start with conserved stretches of poly(T), which are required for 5S rDNA transcription termination. In addition, these sequences showed conservation in the portion of sequence located −25 to −30 nt from the start of the 5S rRNA gene, AGTTAATCT, which is likely a TATA control element (Figure 5A). Interestingly, closer inspection of the longer monomer of TfSat revealed that the sequence ends in a 26 bp segment that represents the fully conserved end of 5S rDNA (Figure 5B). The distribution of the short and long variant of TfSat satDNA, which represents the NTS sequence of the 5SrRNA gene in *T. freemani*, shows that the short variant is associated with arrays on the LG2 chromosome, while the long variant is characteristic for the arrays on LG3 (Supplementary Figure S4B).

To determine whether the 5S rDNA arrays are located at the same genomic sites in these sibling species, we examined the flanking regions around the 5S rDNA arrays for the presence of genes. Two 5S rDNA arrays on LG3 showed collinearity between the species, i.e., identical genes are present in both flanking regions of these arrays. More specifically, the 5S rDNA array at position 28.6 Mb in *T. castaneum* and that at 26.9 Mb in *T. freemani* have the same genes on both sides (LOC659040, LOC660138, and LOC660260). Similarly, the same genes (LOC659040, LOC659881, and Cpsf4) were found in both flanking regions for the 5S rDNA array on 30.7 Mb and on 27.3 Mb in *T. castaneum* and *T. freemani*, respectively.

**Figure 6:**
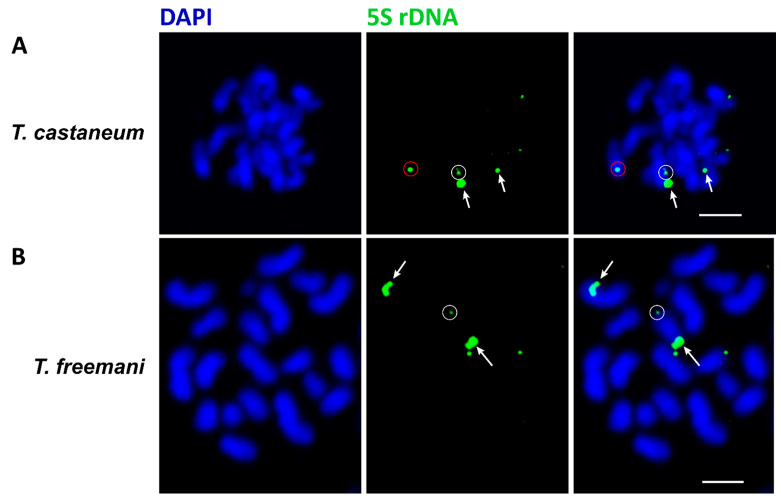
Chromosomes of *T. castaneum* (**A**) and *T. freemani* (**B**) stained by DAPI (blue) after FISH experiments with a biotin-labeled probe specific for 5S rDNA (green). The white arrows show signals of 5S rDNA in the pericentromeric regions, the white circles show the signals located on chromosome 3 and the red circle shows the signal located on chromosome 2. Scale bar: 3 µm.

Finally, we used fluorescence in situ hybridization (FISH) to investigate whether 5S rDNA loci are localized in pericentromeric regions, as these regions are partially assembled in the analyzed long-read-based assemblies (Figure 6).

The results confirmed the three most dominant arrays found in the assembled genomes (circles). In addition, some relatively strong signals (marked by arrows) indicated the presence of 5S rDNA arrays in (peri)centromeric regions of some chromosomes that are not present in the assemblies of *T. castaneum* and *T. freemani*. Our detection of 5S rDNA in the (peri)centromeric regions is in full agreement with the study of the *T. castaneum* centromeres, which revealed that 5S rDNA is found in the pool of sequences closely associated with the centromere-specific protein cCenH3 [22]. In this work, the length of the 5SrRNA was estimated to be 119 bp, in contrast to 121 bp in our work. This small discrepancy in the definition of the 5S RNA gene length is due to the fact that the length determination in the earlier work was based on the automatic 5S annotation.

## 4. Discussion

Improved sequencing technologies that can generate ultra-long reads, together with assembly algorithms, enable deciphering of the structures of complex genomic regions, including those consisting of abundant repetitive sequences. As a result, end-to-end maps of all human chromosomes have recently been generated, including highly repetitive regions such as (peri-)centromeres and telomeres [24]. 5S rRNA genes often form tandemly repeated arrays that can be quite long and are not present even in high-quality genome assemblies [25]. Accordingly, our analysis of the currently NCBI-referenced genome of *T. castaneum*, Tcas5.2 [16], based on BAC sequencing, showed that only a few copies of the 5S rRNA are present in the assembly. In this work, we analyzed the 5S rDNA clusters in *T. castaneum* and its sibling species *T. freemani* using our new, recently provided assemblies generated with long reads such as Nanopore for *T. castaneum* [15] and PacBio HiFi for *T. freemani* [17]. The 5S rRNA genes, regardless of their essentiality in ribogenesis, often show high genomic dynamics in organization, especially in NTS regions, even in closely related species. For this reason, in addition to studying the 5S rRNA genes in *T. castaneum*, an important food pest and the second most popular model insect after *Drosophila* [26], we also included its sibling species *T. freemani* in the study to reveal evolutionary dynamics influencing the genome organization of the 5S rRNA cluster.

The analyses of the 5S rDNA sequences showed a similar pattern in both species with a high intra-species nucleotide similarity of the 5S rRNA genes, which can be considered functional with their length of 121 bp. However, in addition to these functional, full-sized genes, a relatively high proportion of truncated gene sequences with significant deletions of 30 bp were also found, which can be regarded as pseudogenes. Such a finding is not unexpected, because the comprehensive studies of 5S rRNA genes in Metazoa showed that 5S rRNA-coding regions are divided into two types: functional, which are very conserved, and flexible 5S rRNA genes which are much more variable, for example pseudogenes [1]. Pseudogenes result from the duplication of a 5S rRNA gene, followed by degeneration, which is often a sequence deletion and thus leads to nonfunctional copies of the original gene (reviewed in [27]). It is assumed that rRNA genes are maintained in excess of the amount required for ribogenesis, so it is unlikely that even a significant proportion of pseudogenes would affect the functional potential of the 5S rRNA gene in the cell. Our results showed a very low sequence diversity, with only three nucleotide changes between the functional 5S rRNA genes of *T. castaneum* and *T. freemani*. However, even this minor sequence difference causes a change in the secondary structure of the 5S rRNA. This is in accordance with the results of a comparison of the 5S rRNA genes of metazoans and land plants, which shows that considerable conservation of genes and nucleotide changes have an impact on secondary structure that manifests in slight differences in the length of hairpins [28]. In addition, the observed conservation of the secondary structure, especially the stem–loop positions, could be a consequence of sequence evolution through compensatory mutations, which was previously shown for the 18S and 28S rRNA genes.

Our analyses of 5S rDNA cluster organization between *T. castaneum* and *T. freemani* demonstrated an unusually high rate of structural differences between these closely related species. *T. castaneum* exhibits the most common organization of 5S rRNA clusters, characterized by a tandemly repeated unit containing the 5S rRNA gene and a short NTS sequence. In contrast, 5S rRNA clusters in *T. freemani* showed a much more complex organization. *T. freemani* has two types of NTS sequences sharing relatively high sequence similarity, but differing in length. The longer NTS variant is derived from a shorter NTS sequence plus part of the 5S rDNA. Interestingly, both *T. freemani* NTSs in 5SrRNA clusters form tandemly repeated arrays composed exclusively of NTS sequences, so that they resemble satDNA in 5SrRNA clusters. The rearrangement between 5S rRNA and satDNAs has already been observed in some species. For example, in the plant *Plantago lagopus*, PLsatB satDNA includes sequence parts which correspond with 5SrRNA and NTS fragments [29]. A similar process probably produced PcP190 satDNA in frogs, which is derived from the 5S rDNA and NTS [5]. In contrast to these examples, in *T. freemani* NTS sequences themselves show satDNA features forming tandemly arranged arrays in 5S rRNA clusters. However, what makes the cluster organization of the 5S rDNA in *T. freemani* unique is the fact that, in addition to satDNA which probably plays role as NTS, these clusters also contain transposon sequences. Examples of 5S rRNA genes associated with TE elements have also been found in other organisms. For example, so-called Cassandras, the 5S-related nonautonomous retrotransposons in plants, harbor highly conserved 5S rDNA sequences within their long terminal repeats [30]. Moreover, recent work has shown evidence for a gradual coevolution of Cassandra transposons with their corresponding 5S rDNAs [11]. However, the organization of 5S rDNA clusters in *T. freemani* is, as far as we know, the first example of 5S rRNA genes being associated with two types of the most abundant repetitive sequences, satDNA and TE. This organizational pattern supports the hypothesis that 5S rRNA genes exhibit a very dynamic genomic organization independent of their importance for ribogenesis. The question remains as to the biological differences in function or the evolutionary advantage of one 5S rDNA arrangement over another.

Until recently, due to high divergence of NTS sequences, even between related species, it was thought that the NTS had no function. Although comparative studies of the NTS sequences in *T. castaneum* and *T. freemani* show a completely different form of organization, relatively high similarity in NTS sequences, especially in conserved sequence parts is evident. These motifs correspond with control elements such as the start (TATA box) and termination (poly(T)) sites of transcription. This is in accordance with studies of deletion mutants which have shown that NTSs have control elements that are required for the expression of 5S rDNA genes (reviewed in [31]).

In general, two processes, concerted and birth-and-death evolution, have been proposed to be responsible for the observed patterns of evolutionary dynamics of 5S rRNA clusters [32]. Overall, our data suggest that homogenization of 5S rDNA and NTSs, including satDNA and Jockey transposons associated with 5S DNAs, was promoted, thus proclaiming concerted evolution as a crucial process in the evolution of 5S rRNA clusters in *T. castaneum* and *T. freemani*.

## Figures and Tables

**Table 1:** Distribution of 5S rRNA gene in *T. castaneum* and *T. freemani* genome assemblies.

*T. castaneum*			*T. freemani*		
Chromosome	Number of 5S rRNA Genes	Chromosome Position (bp)	Chromosome	Number of 5S rRNA Genes	Chromosome Position (bp)
LG2	6	352,544	fLG2	8	8,321,430
	201	1,470,718		8	17,033,695
LG3	54	28,555,083	fLG3	37	26,902,696
	46	30,727,906		83	27,386,887
Total	307			136	

## Data Availability

The data presented in this study are openly available in: *T. castaneum* genome assembly Tcas5.2 ENA PRJNA15718; *T. castaneum* genome assembly TcasONT ENA PRJEB61413; and *T. freemani* genome assembly ENA accession PRJEB52307. *T. castaneum* genome annotations are available from https://doi.org/10.6084/m9.figshare.22683325.v1 (accessed on 15 March 2024) and *T. freemani* genome annotations from https://doi.org/10.6084/m9.figshare.19682400.v1 (accessed on 15 March 2024).

## Supplementary Materials

The following supporting information can be downloaded at: https://www.mdpi.com/article/10.3390/genes15060776/s1, Figure S1. Alignments of 5S rRNA genes in *T. castaneum* and *T. freemani*. Figure S2. Analysis of 5S cluster integrity and repeat content in assemblies in comparison to raw read data. Figure S3. Distribution of long form of 5S rDNA (green) and short (pseudogene) (red) in *T. castaneum* and *T. freemani*. Figure S4. Alignment of NTS sequences in *T. castaneum* and *T. freemani*. Figure S5. Jockey elements in RepBase from *T. castaneum* and *T. freemani*. Figure S6. Alignment of Jockey elements in *T. castaneum*.
