# Supplementary material for: The Genome Organization of 5S rRNA Genes in the Model Organism Tribolium castaneum and Its Sibling Species Tribolium freemani
Source: Genes (Basel). 2024 Jun 13;15(6):776. doi: 10.3390/genes15060776 (PMC11202950; doi:10.3390/genes15060776)
Supplement: Supplementary file 1 [file genes-15-00776-s001.zip › genes-2984536-supplementary.pdf]

# Supplementary Materials

## A

```
Consensus        GGCAACGTCCATACCACGCTGAATACACCAGTTCTCGTCTGATCACTGAAGTT-AAGCAGCGTCGGGCGCGGTTAGTACTTGGATGGGTGACCGCTTGGG-AACACCGCG-TG-C--CGTTGCCTCC  121
LG3_-_5Scons     .......G........T........T..........................C-.......A......................................-........-..-.--.........--  119
LG3_-_5Scons 2   .....................................................-..............................................-........-..-.--...........  121
LG3_-_5Scons 3   .....................................................-..............................................-........-..-.--...........  121
LG3_-_5Scons 4   .....................................................-..............................................-........-..-.--...........  121
LG3_-_5Scons 5   .....................................................-..............................................-........-..-.--...........  121
LG3_-_5Scons 6   .....................................................-..............................................-........-..-.--...........  121
LG3_-_5Scons 7   .....................................................-..............................................-........-..-.--...........  121
LG3_-_5Scons 8   .....................................................-..............................................-........-..-.--...........  121
LG3_-_5Scons 9   .....................................................-..............................................-........-..-.--...........  121
LG3_-_5Scons 10  .....................................................-..............................................-........-..-.--...........  121
LG3_-_5Scons 11  .....................................................-..............................................-........-..-.--...........  121
LG3_-_5Scons 12  .....................................................-..............................................-........-..-.--...........  121
LG3_-_5Scons 13  .....................................................-..............................................-........-..-.--...........  121
LG3_-_5Scons 14  .....................................................-..............................................-........-..-.--...........  121
LG3_-_5Scons 15  .....................................................-..............................................-........-..-.--...........  121
LG3_-_5Scons 16  .....................................................-..............................................-........-..-.--...........  121
LG3_-_5Scons 17  .....................................................-..............................................-........-..-.--...........  121
LG3_-_5Scons 18  .....................................................-..............................................-........-..-.--...........  121
LG3_-_5Scons 19  .....................................................-..............................................-........-..-.--...........  121
LG3_-_5Scons 20  .....................................................-..............................................-........-..-.--...........  121
LG3_-_5Scons 21  .....................................................-..............................................-........-..-.--...........  121
LG3_-_5Scons 22  .....................................................-..............................................-........-..-.--...........  121
LG3_-_5Scons 23  .....................................................-..............................................-........-..-.--...........  121
LG3_-_5Scons 24  .....................................................-..............................................-........-..-.--...........  121
LG3_-_5Scons 25  .....................................................-..............................................-........-..-.--...........  121
LG3_-_5Scons 26  .....................................................-..............................................-........-..-.--...........  121
LG3_-_5Scons 27  .....................................................-..............................................-........-..-.--...........  121
LG3_-_5Scons 28  .....................................................-..............................................-........-..-.--...........  121
LG3_-_5Scons 29  .....................................................-..............................................-........-..-.--...........  121
LG3_-_5Scons 30  .....................................................-..............................................-........-..-.--...........  121
LG3_-_5Scons 31  .....................................................-..............................................-........-..-.--...........  121
LG3_-_5Scons 32  .....................................................-..............................................-........-..-.--...........  121
LG3_-_5Scons 33  .....................................................-..............................................-........-..-.--...........  121
LG3_-_5Scons 34  .....................................................-..............................................-........-..-.--...........  121
LG3_-_5Scons 35  .....................................................-..............................................-........-..-.--...........  121
LG3_-_5Scons 36  .....................................................-..............................................-........-..-.--...........  121
LG3_-_5Scons 37  .....................................................-..............................................-........-..-.--...........  121
LG3_-_5Scons 38  .....................................................-..............................................-........-..-.--...........  121
LG3_-_5Scons 39  .....................................................-..............................................-........-..-.--...........  121
LG3_-_5Scons 40  .....................................................-..............................................-........-..-.--...........  121
LG3_-_5Scons 41  .....................................................-..............................................-........-..-.--...........  121
LG3_-_5Scons 42  .....................................................-..............................................-........-..-.--...........  121
LG3_-_5Scons 43  .....................................................-..............................................-........-..-.--...........  121
LG3_-_5Scons 44  .....................................................-..............................................-........-..-.--...........  121
LG3_-_5Scons 45  .....................................................-..............................................-........-..-.--...........  121
LG3_-_5Scons 46  .....................................................-..............................................-........-..-.--...........  121
LG3_-_5Scons 47  .....................................................-..............................................-........-..-.--...........  121
LG3_-_5Scons 48  .....................................................-..............................................-........-..-.--...........  121
LG3_-_5Scons 49  .....................................................-..............................................-........-..-.--...........  121
LG3_-_5Scons 50  .....................................................-..............................................-........-..-.--...........  121
LG3_-_5Scons 51  .....................................................-..............................................-........-..-.--...........  121
LG3_-_5Scons 52  .....................................................-..............................................-........-..-.--...........  121
LG3_-_5Scons 53  .....................................................-..............................................-........-..-.--...........  121
LG3_-_5Scons 54  .....................................................-..............................................-........-..-.--...........  121
LG3_-_5Scons 55  .....................................................-..............................................-........-..-.--...........  121
LG3_-_5Scons 56  .....................................................-..............................................-........-..-.--...........  121
LG3_-_5Scons 57  .....................................................-..............................................-........-..-.--...........  121
LG3_-_5Scons 58  .....................................................-..............................................-........-..-.--...........  121
LG3_-_5Scons 59  .....................................................-..............................................-........-..-.--...........  121
LG3_-_5Scons 60  .....................................................-..............................................-........-..-.--...........  121
LG3_-_5Scons 61  .....................................................-..............................................-........-..-.--...........  121
LG3_-_5Scons 62  .....................................................-..............................................-........-..-.--...........  121
LG3_-_5Scons 63  .....................................................-..............................................-........-..-.--...........  121
LG3_-_5Scons 64  .....................................................-..............................................-........-..-.--...........  121
LG3_-_5Scons 65  .....................................................-..............................................-........-..-.--...........  121
LG3_-_5Scons 66  .....................................................-..............................................-........-..-.--...........  121
LG3_-_5Scons 67  .....................................................-..............................................-........-..-.--...........  121
LG3_-_5Scons 68  .....................................................-..............................................-........-..-.--...........  121
LG3_-_5Scons 69  .....................................................-..............................................-........-..-.--...........  121
LG3_-_5Scons 70  .....................................................-..............................................-........-..-.--...........  121
LG3_-_5Scons 71  .....................................................-..............................................-........-..-.--...........  121
LG3_-_5Scons 72  .....................................................-..............................................-........-..-.--...........  121
LG3_-_5Scons 73  .....................................................-..............................................-........-..-.--...........  121
LG3_-_5Scons 74  .....................................................-..............................................-........-..-.--...........  121
LG3_-_5Scons 75  .....................................................-..............................................-........-..-.--...........  121
LG3_-_5Scons 76  .....................................................-..............................................-........-..-.--...........  121
LG3_-_5Scons 77  .....................................................-..............................................-........-..-.--...........  121
LG3_-_5Scons 78  .....................................................-..............................................-........-..-.--...........  121
LG3_-_5Scons 79  .....................................................-..............................................-........-..-.--...........  121
LG3_-_5Scons 80  .....................................................-..............................................-........-..-.--...........  121
LG3_-_5Scons 81  .....................................................-..............................................-........-..-.--...........  121
LG3_-_5Scons 82  .....................................................-..............................................-........-..-.--...........  121
LG3_-_5Scons 83  .....................................................-..............................................-........-..-.--...........  121
LG3_-_5Scons 84  .....................................................-..............................................-........-..-.--...........  121
LG3_-_5Scons 85  .....................................................-..............................................-........-..-.--...........  121
LG3_-_5Scons 86  .....................................................-..............................................-........-..-.--...........  121
LG3_-_5Scons 87  .....................................................-..............................................-........-..-.--...........  121
```

```
LG3_-_5Scons 88  .....................................................-..............................................-.........-..-.-.--..........  121
LG3_-_5Scons 89  .....................................................-..............................................-.........-..-.-.--..........  121
LG3_-_5Scons 90  .....................................................-..............................................-.........-..-.-.--..........  121
LG3_-_5Scons 91  .....................................................-..............................................-.........-..-.-.--..........  121
LG3_-_5Scons 92  .....................................................-..............................................-.........-..-.-.--..........  121
LG3_-_5Scons 93  .....................................................-..............................................-.........-..-.-.--..........  121
LG3_-_5Scons 94  .....................................................-..............................................-.........-..-.-.--..........  121
LG3_-_5Scons 95  .....................................................-..............................................-.........-..-.-.--..........  121
LG3_-_5Scons 96  .....................................................-..............................................-.........-..-.-.--..........  121
LG3_-_5Scons 97  .....................................................-..............................................-.........-..-.-.--..........  121
LG3_-_5Scons 98  .....................................................-..............................................-.........-..-.-.--..........  121
LG3_-_5Scons 99  .....................................................-..............................................-.........-..-.-.--..........  121
LG2_-_5Scons     .....................................................-..............................................-.........-..-.-.--..........  121
LG2_-_5Scons 2   .....................................................-..............................................-.........-..-.-.--..........  121
LG2_-_5Scons 3   .....................................................-..............................................-.........-..-.-.--..........  121
LG2_-_5Scons 4   .....................................................-..............................................-.........-..-.-.--..........  121
LG2_-_5Scons 5   .....................................................-..............................................-.........-..-.-.--..........  121
LG2_-_5Scons 6   .....................................................-..............................................-.........-..-.-.--..........  121
LG2_-_5Scons 7   .....................................................-..............................................-.........-..-.-.--..........  121
LG2_-_5Scons 8   .....................................................-..............................................-.........-..-.-.--..........  121
LG2_-_5Scons 9   .....................................................-..............................................-.........-..-.-.--..........  121
LG2_-_5Scons 10  .....................................................-..............................................-.........-..-.-.--..........  121
LG2_-_5Scons 11  .....................................................-..............................................-.........-..-.-.--..........  121
LG2_-_5Scons 12  .....................................................-..............................................-.........-..-.-.--..........  121
LG2_-_5Scons 13  .....................................................-..............................................-.........-..-.-.--..........  121
LG2_-_5Scons 14  .....................................................-..............................................-.........-..-.-.--..........  121
LG2_-_5Scons 15  .....................................................-..............................................-.........-..-.-.--..........  121
LG2_-_5Scons 16  .....................................................-..............................................-.........-..-.-.--..........  121
LG2_-_5Scons 17  .....................................................-..............................................-.........-..-.-.--..........  121
LG2_-_5Scons 18  .....................................................-..............................................-.........-..-.-.--..........  121
LG2_-_5Scons 19  .....................................................-..............................................-.........-..-.-.--..........  121
LG2_-_5Scons 20  .....................................................-..............................................-.........-..-.-.--..........  121
LG2_-_5Scons 21  .....................................................-..............................................-.........-..-.-.--..........  121
LG2_-_5Scons 22  .....................................................-..............................................-.........-..-.-.--..........  121
LG2_-_5Scons 23  .....................................................-..............................................-.........-..-.-.--..........  121
LG2_-_5Scons 24  .....................................................-..............................................-.........-..-.-.--..........  121
LG2_-_5Scons 25  .....................................................-..............................................-.........-..-.-.--..........  121
LG2_-_5Scons 26  .....................................................-..............................................-.........-..-.-.--..........  121
LG2_-_5Scons 27  .....................................................-..............................................-.........-..-.-.--..........  121
LG2_-_5Scons 28  .....................................................-..............................................-.........-..-.-.--..........  121
LG2_-_5Scons 29  .....................................................-..............................................-.........-..-.-.--..........  121
LG2_-_5Scons 30  .....................................................-..............................................-.........-..-.-.--..........  121
LG2_-_5Scons 31  .....................................................-..............................................-.........-..-.-.--..........  121
LG2_-_5Scons 32  .....................................................-..............................................-.........-..-.-.--..........  121
LG2_-_5Scons 33  .....................................................-..............................................-.........-..-.-.--..........  121
LG2_-_5Scons 34  .....................................................-..............................................-.........-..-.-.--..........  121
LG2_-_5Scons 35  .....................................................-..............................................-.........-..-.-.--..........  121
LG2_-_5Scons 36  .....................................................-..............................................-.........-..-.-.--..........  121
LG2_-_5Scons 37  .....................................................-..............................................-.........-..-.-.--..........  121
LG2_-_5Scons 38  .....................................................-..............................................-.........-..-.-.--..........  121
LG2_-_5Scons 39  .....................................................-..............................................-.........-..-.-.--..........  121
LG2_-_5Scons 40  .....................................................-..............................................-.........-..-.-.--..........  121
LG2_-_5Scons 41  .....................................................-..............................................-.........-..-.-.--..........  121
LG2_-_5Scons 42  .....................................................-..............................................-.........-..-.-.--..........  121
LG2_-_5Scons 43  .....................................................-..............................................-.........-..-.-.--..........  121
LG2_-_5Scons 44  .....................................................-..............................................-.........-..-.-.--..........  121
LG2_-_5Scons 45  .....................................................-..............................................-.........-..-.-.--..........  121
LG2_-_5Scons 46  .....................................................-..............................................-.........-..-.-.--..........  121
LG2_-_5Scons 47  .....................................................-..............................................-.........-..-.-.--..........  121
LG2_-_5Scons 48  .....................................................-..............................................-.........-..-.-.--..........  121
LG2_-_5Scons 49  .....................................................-..............................................-.........-..-.-.--..........  121
LG2_-_5Scons 50  .....................................................-..............................................-.........-..-.-.--..........  121
LG2_-_5Scons 51  .....................................................-..............................................-.........-..-.-.--..........  121
LG2_-_5Scons 52  .....................................................-..............................................-.........-..-.-.--..........  121
LG2_-_5Scons 53  .....................................................-..............................................-.........-..-.-.--..........  121
LG2_-_5Scons 54  .....................................................-..............................................-.........-..-.-.--..........  121
LG2_-_5Scons 55  .....................................................-..............................................-.........-..-.-.--..........  121
LG2_-_5Scons 56  .....................................................-..............................................-.........-..-.-.--..........  121
LG2_-_5Scons 57  .....................................................-..............................................-.........-..-.-.--..........  121
LG2_-_5Scons 58  .....................................................-..............................................-.........-..-.-.--..........  121
LG2_-_5Scons 59  .....................................................-..............................................-.........-..-.-.--..........  121
LG2_-_5Scons 60  .....................................................-..............................................-.........-..-.-.--..........  121
LG2_-_5Scons 61  .....................................................-..............................................-.........-..-.-.--..........  121
LG2_-_5Scons 62  .....................................................-..............................................-.........-..-.-.--..........  121
LG2_-_5Scons 63  .....................................................-..............................................-.........-..-.-.--..........  121
LG2_-_5Scons 64  .....................................................-..............................................-.........-..-.-.--..........  121
LG2_-_5Scons 65  .....................................................-..............................................-.........-..-.-.--..........  121
LG2_-_5Scons 66  .....................................................-..............................................-.........-..-.-.--..........  121
LG2_-_5Scons 67  .....................................................-..............................................-.........-..-.-.--..........  121
LG2_-_5Scons 68  .....................................................-..............................................-.........-..-.-.--..........  121
LG2_-_5Scons 69  .....................................................-..............................................-.........-..-.-.--..........  121
LG2_-_5Scons 70  .....................................................-..............................................-.........-..-.-.--..........  121
LG2_-_5Scons 71  .....................................................-..............................................-.........-..-.-.--..........  121
LG2_-_5Scons 72  .....................................................-..............................................-.........-..-.-.--..........  121
LG2_-_5Scons 73  .....................................................-..............................................-.........-..-.-.--..........  121
LG2_-_5Scons 74  .....................................................-..............................................-.........-..-.-.--..........  121
LG2_-_5Scons 75  .....................................................-..............................................-.........-..-.-.--..........  121
LG2_-_5Scons 76  .....................................................-..............................................-.........-..-.-.--..........  121
LG2_-_5Scons 77  .....................................................-..............................................-.........-..-.-.--..........  121
LG2_-_5Scons 78  .....................................................-..............................................-.........-..-.-.--..........  121
LG2_-_5Scons 79  .....................................................-..............................................-.........-..-.-.--..........  121
LG2_-_5Scons 80  .....................................................-..............................................-.........-..-.-.--..........  121
LG2_-_5Scons 81  .....................................................-..............................................-.........-..-.-.--..........  121
LG2_-_5Scons 82  .....................................................-..............................................-.........-..-.-.--..........  121
LG2_-_5Scons 83  .....................................................-..............................................-.........-..-.-.--..........  121
```

```
LG2_-_5Scons 84  .....................................................-..............................................-.........-..-.--..........  121
LG2_-_5Scons 85  .....................................................-..............................................-.........-..-.--..........  121
LG2_-_5Scons 86  .....................................................-..............................................-.........-..-.--..........  121
LG2_-_5Scons 87  .....................................................-..............................................-.........-..-.--..........  121
LG2_-_5Scons 88  .....................................................-..............................................-.........-..-.--..........  121
LG2_-_5Scons 89  .....................................................-..............................................-.........-..-.--..........  121
LG2_-_5Scons 90  .....................................................-..............................................-.........-..-.--..........  121
LG2_-_5Scons 91  .....................................................-..............................................-.........-..-.--..........  121
LG2_-_5Scons 92  .....................................................-..............................................-.........-..-.--..........  121
LG2_-_5Scons 93  .....................................................-..............................................-.........-..-.--..........  121
LG2_-_5Scons 94  .....................................................-..............................................-.........-..-.--..........  121
LG2_-_5Scons 95  .....................................................-..............................................-.........-..-.--..........  121
LG2_-_5Scons 96  .....................................................-..............................................-.........-..-.--..........  121
LG2_-_5Scons 97  .....................................................-..............................................-.........-..-.--..........  121
LG2_-_5Scons 98  .....................................................-..............................................-.........-..-.--..........  121
LG2_-_5Scons 99  .....................................................-..............................................-.........-..-.--..........  121
LG2_-_5Scons 100 .....................................................-..............................................-.........-..-.--..........  121
LG2_-_5Scons 101 .....................................................-..............................................-.........-..-.--..........  121
LG2_-_5Scons 102 .....................................................-..............................................-.........-..-.--..........  121
LG2_-_5Scons 103 .....................................................-..............................................-.........-..-.--..........  121
LG2_-_5Scons 104 .....................................................-..............................................-.........-..-.--..........  121
LG2_-_5Scons 105 .....................................................-..............................................-.........-..-.--..........  121
LG2_-_5Scons 106 .....................................................-..............................................-.........-..-.--..........  121
LG2_-_5Scons 107 .....................................................-..............................................-.........-..-.--..........  121
LG2_-_5Scons 108 .....................................................-..............................................-.........-..-.--..........  121
LG2_-_5Scons 109 .....................................................-..............................................-.........-..-.--..........  121
LG2_-_5Scons 110 .....................................................-..............................................-.........-..-.--..........  121
LG2_-_5Scons 111 .....................................................-..............................................-.........-..-.--..........  121
LG2_-_5Scons 112 .....................................................-..............................................-.........-..-.--..........  121
LG2_-_5Scons 113 .....................................................-..............................................-.........-..-.--..........  121
LG2_-_5Scons 114 .....................................................-..............................................-.........-..-.--..........  121
LG2_-_5Scons 115 .....................................................-..............................................-.........-..-.--..........  121
LG2_-_5Scons 116 .....................................................-..............................................-.........-..-.--..........  121
LG2_-_5Scons 117 .....................................................-..............................................-.........-..-.--..........  121
LG2_-_5Scons 118 .....................................................-..............................................-.........-..-.--..........  121
LG2_-_5Scons 119 .....................................................-..............................................-.........-..-.--..........  121
LG2_-_5Scons 120 .....................................................-..............................................-.........-..-.--..........  121
LG2_-_5Scons 121 .....................................................-..............................................-.........-..-.--..........  121
LG2_-_5Scons 122 .....................................................-..............................................-.........-..-.--..........  121
LG2_-_5Scons 123 .....................................................-..............................................-.........-..-.--..........  121
LG2_-_5Scons 124 .....................................................-..............................................-.........-..-.--..........  121
LG2_-_5Scons 125 .....................................................-..............................................-.........-..-.--..........  121
LG2_-_5Scons 126 .....................................................-..............................................-.........-..-.--..........  121
LG2_-_5Scons 127 .....................................................-..............................................-.........-..-.--..........  121
LG2_-_5Scons 128 .....................................................-..............................................-.........-..-.--..........  121
LG2_-_5Scons 129 .....................................................-..............................................-.........-..-.--..........  121
LG2_-_5Scons 130 .....................................................-..............................................-.........-..-.--..........  121
LG2_-_5Scons 131 .....................................................-..............................................-.........-..-.--..........  121
LG2_-_5Scons 132 .....................................................-..............................................-.........-..-.--..........  121
LG2_-_5Scons 133 .....................................................-..............................................-.........-..-.--..........  121
LG2_-_5Scons 134 .....................................................-..............................................-.........-..-.--..........  121
LG2_-_5Scons 135 .....................................................-..............................................-.........-..-.--..........  121
LG2_-_5Scons 136 .....................................................-..............................................-.........-..-.--..........  121
LG2_-_5Scons 137 .....................................................-..............................................-.........-..-.--..........  121
LG2_-_5Scons 138 .....................................................-..............................................-.........-..-.--..........  121
LG6_-_5Scons     -----------------------------........................-..............................................-.........-..-.--..........   92
LG2_-_5Scons 139 -----------------------------........................-..............................................-.........-..-.--..........   92
LG2_-_5Scons 140 -....................................................-..............................................-.........-..-.--..........  120
LG2_-_5Scons 141 -....................................................-..............................................-.........-..-.--..........  120
LG2_-_5Scons 142 -....................................................-..............................................-.........-..-.--..........  120
LG2_-_5Scons 143 -....................................................-..............................................-.........-..-.--..........  120
LG2_-_5Scons 144 -....................................................-..............................................-.........-..-.--..........  120
LG2_-_5Scons 145 -....................................................-..............................................-.........-..-.--..........  120
LG2_-_5Scons 146 -....................................................-..............................................-.........-..-.--..........  120
LG2_-_5Scons 147 -....................................................-..............................................-.........-..-.--..........  120
LG2_-_5Scons 148 -....................................................-..............................................-.........-..-.--..........  120
LG2_-_5Scons 149 -....................................................-..............................................-.........-..-.--..........  120
LG2_-_5Scons 150 -....................................................-..............................................-.........-..-.--..........  120
LG2_-_5Scons 151 -....................................................-..............................................-.........-..-.--..........  120
LG2_-_5Scons 152 -....................................................-..............................................-.........-..-.--..........  120
LG2_-_5Scons 153 -....................................................-..............................................-.........-..-.--..........  120
LG2_-_5Scons 154 -....................................................-..............................................-.........-..-.--..........  120
LG2_-_5Scons 155 -....................................................-..............................................-.........-..-.--..........  120
LG2_-_5Scons 156 -....................................................-..............................................-.........-..-.--..........  120
LG2_-_5Scons 157 -....................................................-..............................................-.........-..-.--..........  120
LG3_-_5Scons 100 ........G............................................-..............................................-.........-..-.--..........  121
LG3_-_5Scons 101 ...T.................................................-..............................................-.........-..-.--..........  121
LG2_-_5Scons 158 ...................A.................................-..............................................-.........-..-.--..........  121
LG2_-_5Scons 159 -........A...........................................-..............................................-.........-..-.--..........  120
LG7_-_5Scons     .......G.............................................-..............................................-.........-..-.--........--  119
LG7_-_5Scons 2   .......G.............................................-..............................................-.........-..-.--........--  119
LG5_-_5Scons     .......G.............................................-..............................................-.........-..-.--..........  121
LG2_-_5Scons 160 -----------------------------.............C..........-..............................................-.........-..-.--..........   92
LG2_-_5Scons 161 .....................................................-............................................A.-.........-..-.--..........  121
LG2_-_5Scons 162 .....................................................-..............................................-.........-..-.--......T...  121
LG2_-_5Scons 163 ....................................................C-..............................................-.........-..-.--..........  121
LG2_-_5Scons 164 ....................................................C-..............................................-.........-..-.--..........  121
LG2_-_5Scons 165 ....................................................C-..............................................-.........-..-.--..........  121
LG2_-_5Scons 166 ....................................................C-..............................................-.........-..-.--..........  121
LG2_-_5Scons 167 ....................................................C-..............................................-.........-..-.--..........  121
LG2_-_5Scons 168 ....................................................C-..............................................-.........-..-.--..........  121
LG2_-_5Scons 169 ....................................................C-..............................................-.........-..-.--..........  121
LG2_-_5Scons 170 -...................................................C-..............................................-.........-..-.--..........  120
LG2_-_5Scons 171 -...................................................C-..............................................-.........-..-.--..........  120
LG2_-_5Scons 172 -...................................................C-..............................................-.........-..-.--..........  120
```

```
LG2_-_5Scons 173 -...................................................C-..............................................-.........-..-.--..........  120
LG2_-_5Scons 174 -...................................................C-..............................................-.........-..-.--..........  120
LG2_-_5Scons 175 -...................................................C-..............................................-.........-..-.--..........  120
LG2_-_5Scons 176 -...................................................C-..............................................-.........-..-.--..........  120
LG2_-_5Scons 177 -...................................................C-..............................................-.........-..-.--..........  120
LG2_-_5Scons 178 ....................................................C-..........A...................................-.........-..-.--..........  121
LG3_-_5Scons 102 .....................................................-..............................................-.........-..-.--......A...  121
LG2_-_5Scons 179 -...................................................C-.............................................T-.........-..-.--..........  120
LG2_-_5Scons 180 --...................................................-..............................................-....T....-..-.--..C.......  119
LG2_-_5Scons 181 .....................................................-..............................................-.........-..-.--...A......  121
LG3_-_5Scons 103 .....................................................-..............................................-.........-..C.--..........  122
LG2_-_5Scons 182 -....................................................-..............................................-.........-..C.--..........  121
LG2_-_5Scons 183 -....................................................-..............................................-.........-..C.--..........  121
LG2_-_5Scons 184 -....................................................-..............................................-.........-..C.--..........  121
LG2_-_5Scons 185 -....................................................-..............................................-.........-..C.--..........  121
LG2_-_5Scons 186 ....................................................C-..............................................-.........-..C.--..........  122
LG2_-_5Scons 187 -....................................................-.........................................G..-----------------------------   96
LG2_-_5Scons 188 -....................................................-.........................................G..-----------------------------   96
LG2_-_5Scons 189 -....................................................-.........................................G..-----------------------------   96
LG2_-_5Scons 190 -....................................................-.........................................G..-----------------------------   96
LG2_-_5Scons 191 -....................................................-.........................................G..-----------------------------   96
LG2_-_5Scons 192 -....................................................-.........................................G..-----------------------------   96
LG2_-_5Scons 193 -....................................................-.........................................G..-----------------------------   96
LG2_-_5Scons 194 -....................................................-.........................................G..-----------------------------   96
LG2_-_5Scons 195 -....................................................-.........................................G..-----------------------------   96
LG2_-_5Scons 196 .....................................................-.........................................G..-----------------------------   97
LG2_-_5Scons 197 -....................................................-.........................................G..-------.....T..-.--..........  115
LG2_-_5Scons 198 -....................................................-.................................C.......G..-----------------------------   96
LG2_-_5Scons 199 .....................................................-............................................-.-.........-..-.--..........  120
LG2_-_5Scons 200 -....................................................-..............................................-....-....-..-.--..........  119
LG2_-_5Scons 201 -....................................................-.........................................----.-.........-..-.--..........  116
LG2_-_5Scons 202 -...................................................C-..............................................T.........-..-.--..........  121
LG2_-_5Scons 203 .....................................................-..........................................----------GC..-..-.--..........  112
LG3_-_5Scons 104 .....................................................A..............................................-.........-..-.--..........  122
LG2_-_5Scons 204 -...................................................C--.............................................-.........-..-.--..........  119
LG2_-_5Scons 205 -...................................................C--.............................................-.........-..-.--..........  119
LG2_-_5Scons 207 -----------------------------........................-..............................................-.........-..-.CG..........   94
LG2_-_5Scons 208 --...G...............................................-..................................--------....-.....--..-..-.--..........  109
```

**B**

```
Consensus         GGCAACGGCCATACCACGCTG-AATACACCAGTTCTCGTCTGATCACTGAAGTTAAGCAGCGTCGGGCGCGGTCAGTACTTGGATGGGTGACCGCTTGGGAACACCGCGTGCCGTTGCCTCC  121
fLG3_-_5Scons 2  ................T....-.......................................A.....-------------------------------------------------------   66
fLG3_-_5Scons 3  .....................-......................................................C............................A..............--  119
fLG3_-_5Scons 4  .....................-....................................................................................................  121
fLG3_-_5Scons 5  .....................-....................................................................................................  121
fLG3_-_5Scons 6  .....................-....................................................................................................  121
fLG3_-_5Scons 7  .....................-....................................................................................................  121
fLG3_-_5Scons 8  .....................-....................................................................................................  121
fLG3_-_5Scons 9  .....................-....................................................................................................  121
fLG3_-_5Scons 10 .....................-....................................................................................................  121
fLG3_-_5Scons 11 .....................-....................................................................................................  121
fLG3_-_5Scons 12 .....................-....................................................................................................  121
fLG3_-_5Scons 13 .....................-....................................................................................................  121
fLG3_-_5Scons 14 .....................-....................................................................................................  121
fLG3_-_5Scons 15 .....................-....................................................................................................  121
fLG3_-_5Scons 16 .....................-....................................................................................................  121
fLG3_-_5Scons 17 .....................-....................................................................................................  121
fLG3_-_5Scons 18 .....................-....................................................................................................  121
fLG3_-_5Scons 19 .....................-....................................................................................................  121
fLG3_-_5Scons 20 .....................-....................................................................................................  121
fLG3_-_5Scons 21 .....................-....................................................................................................  121
fLG3_-_5Scons 22 .....................-....................................................................................................  121
fLG3_-_5Scons 23 .....................-....................................................................................................  121
fLG3_-_5Scons 24 .....................-....................................................................................................  121
fLG3_-_5Scons 25 .....................-....................................................................................................  121
fLG3_-_5Scons 26 .....................-....................................................................................................  121
fLG3_-_5Scons 27 .....................-....................................................................................................  121
fLG3_-_5Scons 28 .....................-....................................................................................................  121
fLG3_-_5Scons 29 .....................-....................................................................................................  121
fLG3_-_5Scons 30 .....................-....................................................................................................  121
fLG3_-_5Scons 31 .....................-....................................................................................................  121
fLG3_-_5Scons 32 .....................-....................................................................................................  121
fLG3_-_5Scons 33 .....................-....................................................................................................  121
fLG3_-_5Scons 34 .....................-....................................................................................................  121
fLG3_-_5Scons 35 .....................-....................................................................................................  121
fLG3_-_5Scons 36 .....................-....................................................................................................  121
fLG3_-_5Scons 37 .....................-....................................................................................................  121
fLG3_-_5Scons 38 .....................-....................................................................................................  121
fLG3_-_5Scons 39 .....................-....................................................................................................  121
fLG3_-_5Scons 40 .....................-....................................................................................................  121
fLG3_-_5Scons 41 .....................-....................................................................................................  121
fLG3_-_5Scons 42 .....................-....................................................................................................  121
fLG3_-_5Scons 43 .....................-....................................................................................................  121
fLG3_-_5Scons 44 .....................-....................................................................................................  121
fLG3_-_5Scons 45 .....................-....................................................................................................  121
fLG3_-_5Scons 46 .....................-....................................................................................................  121
fLG3_-_5Scons 47 .....................-....................................................................................................  121
fLG3_-_5Scons 48 .....................-....................................................................................................  121
fLG3_-_5Scons 49 .....................-....................................................................................................  121
fLG3_-_5Scons 50 .....................-....................................................................................................  121
fLG3_-_5Scons 51 .....................-....................................................................................................  121
fLG3_-_5Scons 52 .....................-....................................................................................................  121
```

```
fLG3_-_5Scons 53   .....................-....................................................................................................  121
fLG3_-_5Scons 54   .....................-....................................................................................................  121
fLG3_-_5Scons 55   .....................-....................................................................................................  121
fLG3_-_5Scons 56   .....................-....................................................................................................  121
fLG3_-_5Scons 57   .....................-....................................................................................................  121
fLG3_-_5Scons 58   .....................-....................................................................................................  121
fLG3_-_5Scons 59   .....................-....................................................................................................  121
fLG3_-_5Scons 60   .....................-....................................................................................................  121
fLG3_-_5Scons 61   .....................-....................................................................................................  121
fLG3_-_5Scons 62   .....................-....................................................................................................  121
fLG3_-_5Scons 63   .....................-....................................................................................................  121
fLG3_-_5Scons 64   .....................-....................................................................................................  121
fLG3_-_5Scons 65   .....................-....................................................................................................  121
fLG3_-_5Scons 66   .....................-....................................................................................................  121
fLG3_-_5Scons 67   .....................-....................................................................................................  121
fLG3_-_5Scons 68   .....................-....................................................................................................  121
fLG3_-_5Scons 69   .....................-....................................................................................................  121
fLG3_-_5Scons 70   .....................-....................................................................................................  121
fLG3_-_5Scons 71   .....................-....................................................................................................  121
fLG3_-_5Scons 72   .....................-....................................................................................................  121
fLG3_-_5Scons 73   .....................-....................................................................................................  121
fLG3_-_5Scons 74   .....................-....................................................................................................  121
fLG3_-_5Scons 75   .....................-....................................................................................................  121
fLG3_-_5Scons 76   .....................-....................................................................................................  121
fLG3_-_5Scons 77   .....................-....................................................................................................  121
fLG3_-_5Scons 78   .....................-....................................................................................................  121
fLG3_-_5Scons 79   .....................-....................................................................................................  121
fLG3_-_5Scons 80   .....................-....................................................................................................  121
fLG3_-_5Scons 81   .....................-....................................................................................................  121
fLG3_-_5Scons 82   .....................-....................................................................................................  121
fLG3_-_5Scons 83   .....................-....................................................................................................  121
fLG3_-_5Scons 84   .....................-....................................................................................................  121
fLG3_-_5Scons 85   .....................-....................................................................................................  121
fLG3_-_5Scons 86   .....................-....................................................................................................  121
fLG2_-_5Scons      .....................-....................................................................................................  121
fLG2_-_5Scons 2    .....................-....................................................................................................  121
fLG3_-_5Scons 87   .....................-..................................................................................................--  119
fLG2_-_5Scons 3    ------------------------------........................................................................................----   88
fLG2_-_5Scons 4    -------------------------------.......................................................................................----   87
fLG3_-_5Scons 88   ...........A.........-....................................................................................................  121
fLG3_-_5Scons 89   ------------------------------............................................................................................   92
fLG3_-_5Scons 90   ------------------------------............................................................................................   92
fLG3_-_5Scons 91   ------------------------------............................................................................................   92
fLG3_-_5Scons 92   ------------------------------............................................................................................   92
fLG3_-_5Scons 93   ------------------------------............................................................................................   92
fLG3_-_5Scons 94   ------------------------------............................................................................................   92
fLG3_-_5Scons 95   ------------------------------............................................................................................   92
fLG3_-_5Scons 96   ------------------------------............................................................................................   92
fLG3_-_5Scons 97   ------------------------------............................................................................................   92
fLG3_-_5Scons 98   ------------------------------............................................................................................   92
fLG3_-_5Scons 99   ------------------------------............................................................................................   92
fLG3_-_5Scons 100  ------------------------------............................................................................................   92
fLG3_-_5Scons 101  ------------------------------............................................................................................   92
fLG2_-_5Scons 5    ------------------------------............................................................................................   92
fLG2_-_5Scons 6    ------------------------------............................................................................................   92
fLG2_-_5Scons 7    ------------------------------............................................................................................   92
fLG2_-_5Scons 8    ------------------------------............................................................................................   92
fLG2_-_5Scons 9    ------------------------------............................................................................................   92
fLG2_-_5Scons 10   ------------------------------...........................................................................................-   91
fLG2_-_5Scons 11   ------------------------------...........................................................................................-   91
fLG2_-_5Scons 12   ------------------------------...........................................................................................-   91
fLG2_-_5Scons 13   ------------------------------...........................................................................................-   91
fLG3_-_5Scons 102  .....................-.....................................A..............................................................  121
fLG3_-_5Scons 103  .....................-.....................................A..............................................................  121
fLG3_-_5Scons 104  .....................-..................................................................C.................................  121
fLG3_-_5Scons 105  .....................-.............................................T......................................................  121
fLG3_-_5Scons 106  .....................-.............................................T......................................................  121
fLG3_-_5Scons 107  .....................-.............................................T......................................................  121
fLG3_-_5Scons 108  .....................-.............................................T......................................................  121
fLG3_-_5Scons 109  .....................-.............................................T......................................................  121
fLG3_-_5Scons 110  .....................-.............................................T......................................................  121
fLG3_-_5Scons 111  .....................-.............................................T......................................................  121
fLG3_-_5Scons 112  .....................-.............................................T......................................................  121
fLG2_-_5Scons 14   .....................-.........................................................................C.........................-  120
fLG2_-_5Scons 15   .....................-.........A...............................................................C.........................-  120
fLG2_-_5Scons 16   ------------------------------.................................................................C..........................   92
fLG3_-_5Scons 113  ------------------------------..............................................................................A.............   92
fLG3_-_5Scons 114  ------------------------------..............................................................................A.............   92
fLG3_-_5Scons 115  ------------------------------..................................A.........................................................   92
fLG3_-_5Scons 116  .....................-.............A......................................................................................  121
fLG3_-_5Scons 117  .....................-......................A.............................................................................  121
fLG3_-_5Scons 118  .....................-......................A.............................................................................  121
fLG3_-_5Scons 119  ------------------------------..............A.............................................................................   92
fLG3_-_5Scons 120  ------------------------------...............................A...........T..............................................--   90
fLG3_-_5Scons 121  ------------------------------.....................................................................................A......   92
fLG3_-_5Scons 122  ------------------------------..............--..T..C..........................................T.......T.................--   88
fLG9_-_5Scons      ..................T..A..A.T.............C............C.....A........................................................------  118
```

**Figure S1.** Alignments of 5S rRNA genes in *T. castaneum* (**A**) and *T. freemani* (**B**). Red dashed lines indicate the regions deleted in some monomer subgroups.

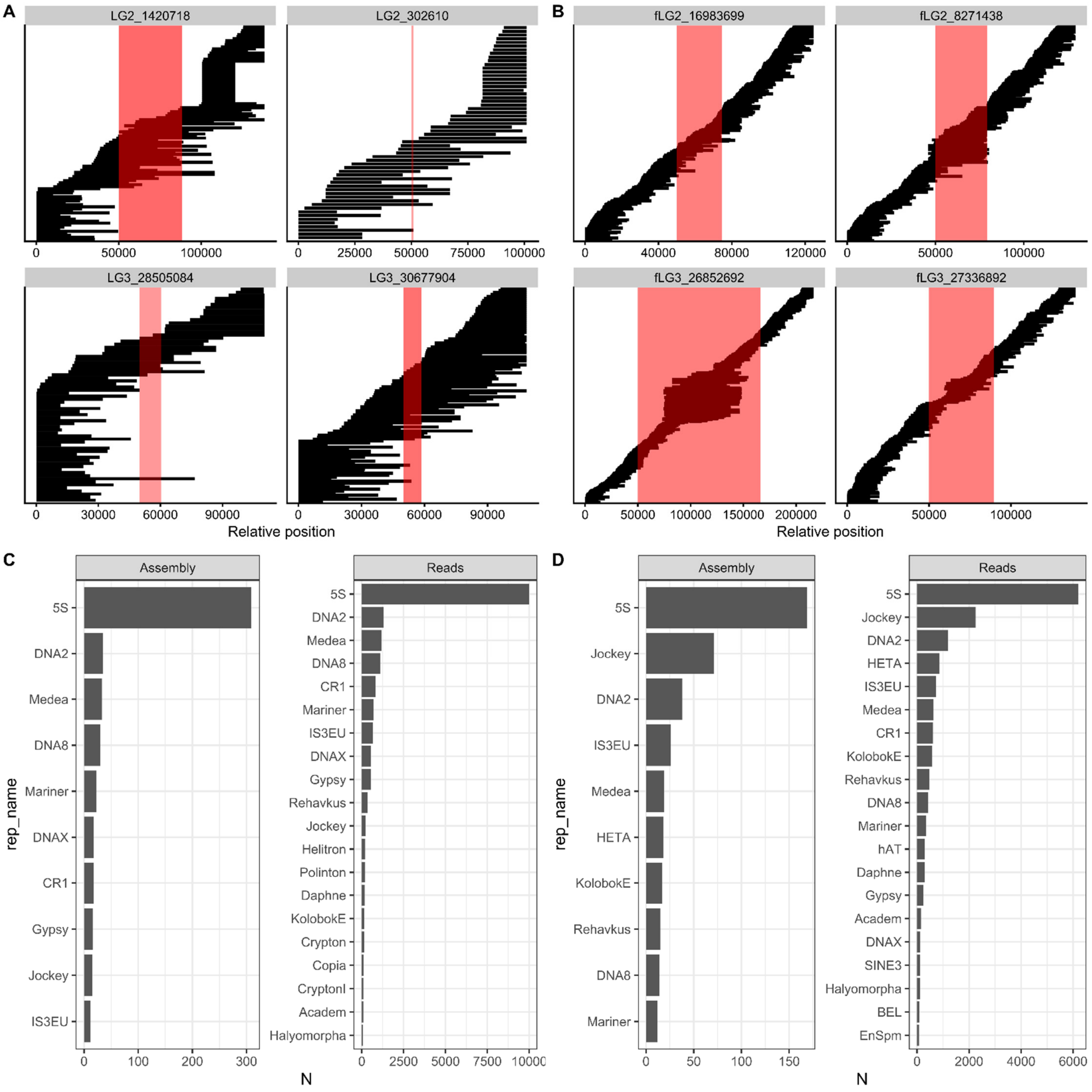


**Figure S2. Analysis of 5S cluster integrity and repeat content in assemblies in comparison to raw read data. (A)** Mappigns of raw ONT reads onto 4 defined clusters (red) of 5S rDNA in TcasONT together with +/-50 kb flank regions. (**B)** Mappigns of raw PacBio reads onto 4 defined clusters (red) of 5S rDNA in Tfree_1.0 together with +/-50 kb flank regions. (**C)** Repeat content analysis of 5S rDNA arrays with +/- 50 kb flank regions in TcasONT and corresponding reads. (**D)** Repeat content analysis of 5S rDNA arrays with +/- 50 kb flank regions in Tfree_1.0 and corresponding reads.

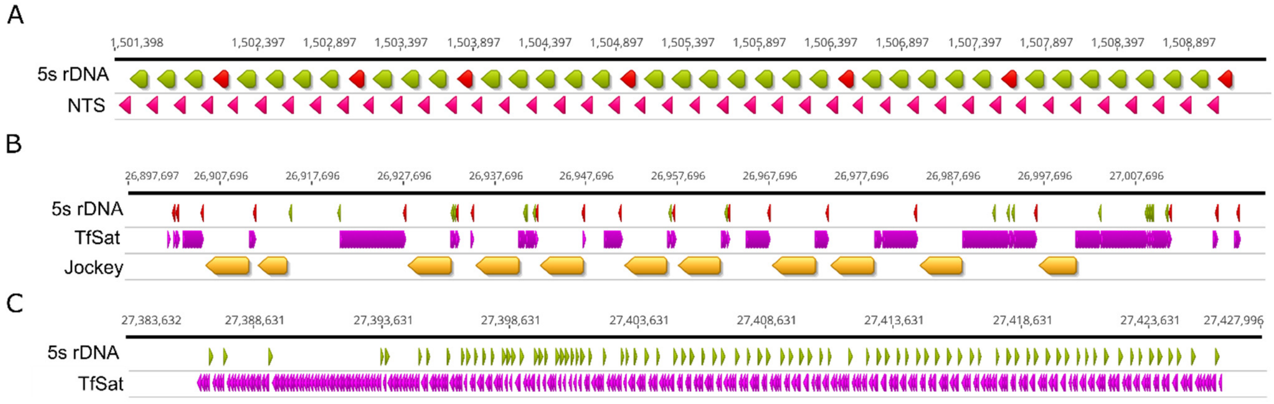


**Figure S3.** Distribution of long form of 5S rDNA (green) and short (pseudogene) (red) in *T. castaneum* and *T. freemani*. **A)** Mix of both long and short form of rRNA genes found on LG2 5s rDNA arrays in *T. castaneum*. **B)** Mix of both long and short form of 5s rRNA genes are found in arrays with Jockey elements in *T. freemani*. **C)** Only long form of 5s rRNA genes is found in arrays without Jockey elements in *T. freemani*.

**A**

```
Consensus                   TTTTTTGTTTTATAACGCTTTAAATAAAA-TTTTAG--GACAGTTAATC-TACTCTGAGGACGAGAAAACGTTCA  71
Cast_NTS_long_LG2_352743    .............................-......--...........-.........................  71
Cast_NTS_long_LG2_353509    .............................-......--...........-.........................  71
Cast_NTS_long_LG2_1471229   .............................-......--...........-.........................  71
Cast_NTS_long_LG2_1471998   .............................-......--...........-.........................  71
Cast_NTS_long_LG2_1472381   .............................-......--...........-.........................  71
Cast_NTS_long_LG2_1472957   .............................-......--...........-.........................  71
Cast_NTS_long_LG2_1473342   .............................-......--...........-.........................  71
Cast_NTS_long_LG2_1474108   .............................-......--...........-.........................  71
Cast_NTS_long_LG2_1475259   .............................-......--...........-.........................  71
Cast_NTS_long_LG2_1475643   .............................-......--...........-.........................  71
Cast_NTS_long_LG2_1476794   .............................-......--...........-.........................  71
Cast_NTS_long_LG2_1477370   .............................-......--...........-.........................  71
Cast_NTS_long_LG2_1477945   .............................-......--...........-.........................  71
Cast_NTS_long_LG2_1478713   .............................-......--...........-.........................  71
Cast_NTS_long_LG2_1479289   .............................-......--...........-.........................  71
Cast_NTS_long_LG2_1480056   .............................-......--...........-.........................  71
Cast_NTS_long_LG2_1480821   .............................-......--...........-.........................  71
Cast_NTS_long_LG2_1481398   .............................-......--...........-.........................  71
Cast_NTS_long_LG2_1481782   .............................-......--...........-.........................  71
Cast_NTS_long_LG2_1481974   .............................-......--...........-.........................  71
Cast_NTS_long_LG2_1482166   .............................-......--...........-.........................  71
Cast_NTS_long_LG2_1482358   .............................-......--...........-.........................  71
Cast_NTS_long_LG2_1482741   .............................-......--...........-.........................  71
Cast_NTS_long_LG2_1482933   .............................-......--...........-.........................  71
Cast_NTS_long_LG2_1483318   .............................-......--...........-.........................  71
Cast_NTS_long_LG2_1483510   .............................-......--...........-.........................  71
Cast_NTS_long_LG2_1483897   .............................-......--...........-.........................  71
Cast_NTS_long_LG2_1484268   .............................-......--...........-.........................  71
Cast_NTS_long_LG2_1484460   .............................-......--...........-.........................  71
Cast_NTS_long_LG2_1484652   .............................-......--...........-.........................  71
Cast_NTS_long_LG2_1484844   .............................-......--...........-.........................  71
Cast_NTS_long_LG2_1485036   .............................-......--...........-.........................  71
Cast_NTS_long_LG2_1485415   .............................-......--...........-.........................  71
Cast_NTS_long_LG2_1485990   .............................-......--...........-.........................  71
Cast_NTS_long_LG2_1486182   .............................-......--...........-.........................  71
Cast_NTS_long_LG2_1486567   .............................-......--...........-.........................  71
Cast_NTS_long_LG2_1486946   .............................-......--...........-.........................  71
Cast_NTS_long_LG2_1487138   .............................-......--...........-.........................  71
Cast_NTS_long_LG2_1487330   .............................-......--...........-.........................  71
Cast_NTS_long_LG2_1487701   .............................-......--...........-.........................  71
Cast_NTS_long_LG2_1488085   .............................-......--...........-.........................  71
Cast_NTS_long_LG2_1488277   .............................-......--...........-.........................  71
Cast_NTS_long_LG2_1488469   .............................-......--...........-.........................  71
Cast_NTS_long_LG2_1488840   .............................-......--...........-.........................  71
Cast_NTS_long_LG2_1489412   .............................-......--...........-.........................  71
Cast_NTS_long_LG2_1489604   .............................-......--...........-.........................  71
```

```
Cast_NTS_long_LG2_1489975  .............................-......--...........-.........................  71
Cast_NTS_long_LG2_1490167  .............................-......--...........-.........................  71
Cast_NTS_long_LG2_1490743  .............................-......--...........-.........................  71
Cast_NTS_long_LG2_1491878  .............................-......--...........-.........................  71
Cast_NTS_long_LG2_1492070  .............................-......--...........-.........................  71
Cast_NTS_long_LG2_1492838  .............................-......--...........-.........................  71
Cast_NTS_long_LG2_1493222  .............................-......--...........-.........................  71
Cast_NTS_long_LG2_1493414  .............................-......--...........-.........................  71
Cast_NTS_long_LG2_1493606  .............................-......--...........-.........................  71
Cast_NTS_long_LG2_1494182  .............................-......--...........-.........................  71
Cast_NTS_long_LG2_1494950  .............................-......--...........-.........................  71
Cast_NTS_long_LG2_1495334  .............................-......--...........-.........................  71
Cast_NTS_long_LG2_1495526  .............................-......--...........-.........................  71
Cast_NTS_long_LG2_1495908  .............................-......--...........-.........................  71
Cast_NTS_long_LG2_1496292  .............................-......--...........-.........................  71
Cast_NTS_long_LG2_1496484  .............................-......--...........-.........................  71
Cast_NTS_long_LG2_1496676  .............................-......--...........-.........................  71
Cast_NTS_long_LG2_1496868  .............................-......--...........-.........................  71
Cast_NTS_long_LG2_1497060  .............................-......--...........-.........................  71
Cast_NTS_long_LG2_1497444  .............................-......--...........-.........................  71
Cast_NTS_long_LG2_1497828  .............................-......--...........-.........................  71
Cast_NTS_long_LG2_1498020  .............................-......--...........-.........................  71
Cast_NTS_long_LG2_1498212  .............................-......--...........-.........................  71
Cast_NTS_long_LG2_1498404  .............................-......--...........-.........................  71
Cast_NTS_long_LG2_1498775  .............................-......--...........-.........................  71
Cast_NTS_long_LG2_1499160  .............................-......--...........-.........................  71
Cast_NTS_long_LG2_1499352  .............................-......--...........-.........................  71
Cast_NTS_long_LG2_1499727  .............................-......--...........-.........................  71
Cast_NTS_long_LG2_1500110  .............................-......--...........-.........................  71
Cast_NTS_long_LG2_1500302  .............................-......--...........-.........................  71
Cast_NTS_long_LG2_1500494  .............................-......--...........-.........................  71
Cast_NTS_long_LG2_1500878  .............................-......--...........-.........................  71
Cast_NTS_long_LG2_1501262  .............................-......--...........-.........................  71
Cast_NTS_long_LG2_1501624  .............................-......--...........-.........................  71
Cast_NTS_long_LG2_1501816  .............................-......--...........-.........................  71
Cast_NTS_long_LG2_1502187  .............................-......--...........-.........................  71
Cast_NTS_long_LG2_1502572  .............................-......--...........-.........................  71
Cast_NTS_long_LG2_1502764  .............................-......--...........-.........................  71
Cast_NTS_long_LG2_1503135  .............................-......--...........-.........................  71
Cast_NTS_long_LG2_1503890  .............................-......--...........-.........................  71
Cast_NTS_long_LG2_1505605  .............................-......--...........-.........................  71
Cast_NTS_long_LG2_1506181  .............................-......--...........-.........................  71
Cast_NTS_long_LG2_1506552  .............................-......--...........-.........................  71
Cast_NTS_long_LG2_1507320  .............................-......--...........-.........................  71
Cast_NTS_long_LG2_1507691  .............................-......--...........-.........................  71
Cast_NTS_long_LG2_1508074  .............................-......--...........-.........................  71
Cast_NTS_long_LG2_1482550  -............................-......--...........-.........................  70
Cast_NTS_long_LG2_1491495  -............................-......--...........-.........................  70
Cast_NTS_long_LG2_1499919  -............................-......--...........-.........................  70
Cast_NTS_long_LG2_352546   .............................-......--...........-...................T.....  71
Cast_NTS_long_LG2_1471613  ...C.........................-......--...........-.........................  71
Cast_NTS_long_LG2_1498967  ...C.........................-......--...........-.........................  71
Cast_NTS_long_LG2_1501431  ...C.........................-......--...........-.........................  71
Cast_NTS_long_LG2_1502379  ...C.........................-......--...........-.........................  71
Cast_NTS_long_LG2_1472573  .............................-......--..........T-.........................  71
Cast_NTS_long_LG2_1474300  .............................-......--..........T-.........................  71
Cast_NTS_long_LG2_1474876  .............................-......--..........T-.........................  71
Cast_NTS_long_LG2_1475835  .............................-......--..........T-.........................  71
Cast_NTS_long_LG2_1476218  .............................-......--..........T-.........................  71
Cast_NTS_long_LG2_1478329  .............................-......--..........T-.........................  71
Cast_NTS_long_LG2_1478905  .............................-......--..........T-.........................  71
Cast_NTS_long_LG2_1479672  .............................-......--..........T-.........................  71
Cast_NTS_long_LG2_1485607  .............................-......--..........T-.........................  71
Cast_NTS_long_LG2_1486375  .............................-......--..........T-.........................  71
Cast_NTS_long_LG2_1487522  .............................-......--..........T-.........................  71
Cast_NTS_long_LG2_1487893  .............................-......--..........T-.........................  71
Cast_NTS_long_LG2_1489221  .............................-......--..........T-.........................  71
Cast_NTS_long_LG2_1490551  .............................-......--..........T-.........................  71
Cast_NTS_long_LG2_1490935  .............................-......--..........T-.........................  71
Cast_NTS_long_LG2_1492262  .............................-......--..........T-.........................  71
Cast_NTS_long_LG2_1492454  .............................-......--..........T-.........................  71
Cast_NTS_long_LG2_1492646  .............................-......--..........T-.........................  71
Cast_NTS_long_LG2_1493798  .............................-......--..........T-.........................  71
Cast_NTS_long_LG2_1493990  .............................-......--..........T-.........................  71
Cast_NTS_long_LG2_1494374  .............................-......--..........T-.........................  71
Cast_NTS_long_LG2_1494758  .............................-......--..........T-.........................  71
Cast_NTS_long_LG2_1495142  .............................-......--..........T-.........................  71
Cast_NTS_long_LG2_1497252  .............................-......--..........T-.........................  71
Cast_NTS_long_LG2_1499544  .............................-......--..........T-.........................  71
Cast_NTS_long_LG2_1500686  .............................-......--..........T-.........................  71
Cast_NTS_long_LG2_1503327  .............................-......--..........T-.........................  71
Cast_NTS_long_LG2_1504082  .............................-......--..........T-.........................  71
Cast_NTS_long_LG2_1504274  .............................-......--..........T-.........................  71
Cast_NTS_long_LG2_1504466  .............................-......--..........T-.........................  71
Cast_NTS_long_LG2_1504658  .............................-......--..........T-.........................  71
Cast_NTS_long_LG2_1505029  .............................-......--..........T-.........................  71
Cast_NTS_long_LG2_1505221  .............................-......--..........T-.........................  71
Cast_NTS_long_LG2_1506744  .............................-......--..........T-.........................  71
Cast_NTS_long_LG2_1507128  .............................-......--..........T-.........................  71
Cast_NTS_long_LG2_1507882  .............................-......--..........T-.........................  71
Cast_NTS_long_LG2_1508266  .............................-......--..........T-.........................  71
Cast_NTS_long_LG2_1508842  .............................-......--..........T-.........................  71
Cast_NTS_long_LG2_1491304  -............................-......--..........T-.........................  70
Cast_NTS_long_LG2_1484089  .............................-......--..T.......T-.........................  71
Cast_NTS_long_LG2_1485228  .............................-......--..T.......T-.........................  71
```

```
Cast_NTS_long_LG2_1488661  .............................-......--..T.......T-.........................  71
Cast_NTS_long_LG2_1489796  .............................-......--..T.......T-.........................  71
Cast_NTS_long_LG2_1498596  .............................-......--..T.......T-.........................  71
Cast_NTS_long_LG2_1502008  .............................-......--..T.......T-.........................  71
Cast_NTS_long_LG2_1502956  .............................-......--..T.......T-.........................  71
Cast_NTS_long_LG2_1503711  .............................-......--..T.......T-.........................  71
Cast_NTS_long_LG2_1504850  .............................-......--..T.......T-.........................  71
Cast_NTS_long_LG2_1505989  .............................-......--..T.......T-.........................  71
Cast_NTS_long_LG2_1506373  .............................-......--..T.......T-.........................  71
Cast_NTS_long_LG2_1507512  .............................-......--..T.......T-.........................  71
Cast_NTS_long_LG3_28555020 .............................-C.....--...........-...................T.....  71
Cast_NTS_long_LG3_30736517 .............................-C.....--...........-...................T.....  71
Cast_NTS_long_LG2_1474684  .............................-......--...........-........AA...............  71
Cast_NTS_long_LG2_1479097  .............................-......--...........-........AA...............  71
Cast_NTS_long_LG2_1481206  .............................-......--...........-........AA...............  71
Cast_NTS_long_LG2_1490359  .............................-......--..........G-.........................  71
Cast_NTS_long_LG2_1493030  .............................-......--..........G-.........................  71
Cast_NTS_long_LG2_1491686  .............................-......--..........G-....T....................  71
Cast_NTS_long_LG2_353126   .............................-......--...........-.....................G...  71
Cast_NTS_long_LG2_1505413  .................TC..........-......--..........T-.........................  71
Cast_NTS_long_LG2_1475068  -................TC..........-......--..........T-.........................  70
Cast_NTS_long_LG2_1494566  .............................-......--..........G-.C..T....................  71
Cast_NTS_long_LG2_1496100  .............................-......--..........G-.C..T....................  71
Cast_NTS_long_LG2_1501070  .............................-......--..........G-.C..T....................  71
Cast_NTS_long_LG2_1509034  .............................-......--..........G-.C..T....................  71
Cast_NTS_long_LG2_1471037  .............................-......--..........G-.C..T....A...............  71
Cast_NTS_long_LG2_1471806  .............................-......--..........G-.C..T....A...............  71
Cast_NTS_long_LG2_1472190  .............................-......--..........G-.C..T....A...............  71
Cast_NTS_long_LG2_1472765  .............................-......--..........G-.C..T....A...............  71
Cast_NTS_long_LG2_1473149  .............................-......--..........G-.C..T....A...............  71
Cast_NTS_long_LG2_1473534  .............................-......--..........G-.C..T....A...............  71
Cast_NTS_long_LG2_1473917  .............................-......--..........G-.C..T....A...............  71
Cast_NTS_long_LG2_1474492  .............................-......--..........G-.C..T....A...............  71
Cast_NTS_long_LG2_1475451  .............................-......--..........G-.C..T....A...............  71
Cast_NTS_long_LG2_1476410  .............................-......--..........G-.C..T....A...............  71
Cast_NTS_long_LG2_1476602  .............................-......--..........G-.C..T....A...............  71
Cast_NTS_long_LG2_1476986  .............................-......--..........G-.C..T....A...............  71
Cast_NTS_long_LG2_1477178  .............................-......--..........G-.C..T....A...............  71
Cast_NTS_long_LG2_1477753  .............................-......--..........G-.C..T....A...............  71
Cast_NTS_long_LG2_1478137  .............................-......--..........G-.C..T....A...............  71
Cast_NTS_long_LG2_1478521  .............................-......--..........G-.C..T....A...............  71
Cast_NTS_long_LG2_1479864  .............................-......--..........G-.C..T....A...............  71
Cast_NTS_long_LG2_1480248  .............................-......--..........G-.C..T....A...............  71
Cast_NTS_long_LG2_1481590  .............................-......--..........G-.C..T....A...............  71
Cast_NTS_long_LG2_1497636  .............................-......--..........G-.C..T....A...............  71
Cast_NTS_long_LG2_1503519  .............................-......--..........G-.C..T....A...............  71
Cast_NTS_long_LG2_1505797  .............................-......--..........G-.C..T....A...............  71
Cast_NTS_long_LG2_1506936  .............................-......--..........G-.C..T....A...............  71
Cast_NTS_long_LG2_1508458  .............................-......--..........G-.C..T....A...............  71
Cast_NTS_long_LG2_1508650  .............................-......--..........G-.C..T....A...............  71
Cast_NTS_long_LG2_1473726  -............................-......--..........G-.C..T....A...............  70
Cast_NTS_long_LG2_1477562  -............................-......--..........G-.C..T....A...............  70
Cast_NTS_long_LG2_1480630  -............................-......--..........G-.C..T....A...............  70
Cast_NTS_long_LG2_1471421  ....C........................-......--..........G-.C..T....A...............  71
Cast_NTS_long_LG2_1476027  -..........................C.-......--..........G-.C..T....A...............  70
Cast_NTS_long_LG2_1495718  --....T.G....................-......--..........T-.........................  69
Cast_NTS_long_LG3_28556563 ..................C..........-C...TA--...........-.........................  71
Cast_NTS_long_LG3_28557911 ..................C..........-C...TA--...........-.C..T....................  71
Cast_NTS_long_LG3_28558682 -.................C..........-C...TA--...........-.C..T....................  70
Cast_NTS_long_LG2_1480440  -............................-......--..........T-A-.......................  69
Cast_NTS_long_LG2_1486759  .............................-......--..........T-.-.......................  70
Cast_NTS_long_LG2_1479481  .............................-......--..........G-.-..T....................  70
Cast_NTS_long_LG2_1485799  .............................-......--..........G-.-..T....A...............  70
Cast_NTS_long_LG3_28557334 ..................C..........-C...TA--...........-.-.CT....................  70
Cast_NTS_long_LG2_1481013  .............................-......--...........T.........................  72
Cast_NTS_long_LG2_1483125  .............................-......--..........TC.........................  72
Cast_NTS_long_LG2_1491113  .............................-......--...........-........-................  70
Cast_NTS_long_LG2_353318   ..............G..............-.....T--...........-............A....-......  70
Cast_NTS_long_LG2_1489032  .............................-......--..........----T.T....................  68
Cast_NTS_long_LG2_1483703  .............................-......GA...........-.........................  73
Cast_NTS_long_LG2_1470653  .............................C......--..A........-...................T.....  72
Cast_NTS_long_LG3_28560031 ..................C..........C......--...........-.........................  72
Cast_NTS_long_LG3_28561189 ..................C..........C......--...........-.........................  72
Cast_NTS_long_LG3_28561382 ..................C..........C......--...........-.........................  72
Cast_NTS_long_LG3_28564085 ..................C..........C......--...........-.........................  72
Cast_NTS_long_LG3_28555598 ...........G......C..........C......--...........-.........................  72
Cast_NTS_long_LG3_28560996 ...........G......C..........C......--...........-.........................  72
Cast_NTS_long_LG3_28560803 .................TC..........C......--...........-.........................  72
Cast_NTS_long_LG3_30729183 ..................C..........C......--.....C.....-.........................  72
Cast_NTS_long_LG3_30730341 ..................C..........C......--.....C.....-.........................  72
Cast_NTS_long_LG3_30730727 ..................C..........C......--.....C.....-.........................  72
Cast_NTS_long_LG3_30734394 ..................C..........C......--.....C.....-.........................  72
Cast_NTS_long_LG3_30734780 ..................C..........C......--.....C.....-.........................  72
Cast_NTS_long_LG3_28557718 ..................C..........C......--A..........-.........................  72
Cast_NTS_long_LG3_28558296 ..................C..........C......--A..........-.........................  72
Cast_NTS_long_LG3_28558604 ..................C..........C......--A..........-.........................  72
Cast_NTS_long_LG3_30728990 ..................C..........C......--A..........-.........................  72
Cast_NTS_long_LG3_30729569 ..................C..........C......--A..........-.........................  72
Cast_NTS_long_LG3_30730148 ..................C..........C......--A..........-.........................  72
Cast_NTS_long_LG3_30731114 ..................C..........C......--A..........-.........................  72
Cast_NTS_long_LG3_30732079 ..................C..........C......--A..........-.........................  72
Cast_NTS_long_LG3_30733044 ..................C..........C......--A..........-.........................  72
Cast_NTS_long_LG3_30733623 ..................C..........C......--A..........-.........................  72
Cast_NTS_long_LG3_30733816 ..................C..........C......--A..........-.........................  72
Cast_NTS_long_LG3_30735166 ..................C..........C......--A..........-.........................  72
```

```
Cast_NTS_long_LG3_30735552 ..................C..........C......--A..........-.........................  72
Cast_NTS_long_LG3_30734202 -.................C..........C......--A..........-.........................  71
Cast_NTS_long_LG3_30729376 ..................C..........C......--A..........-..............A..........  72
Cast_NTS_long_LG3_30730920 ..................C..........C......--A..........-..............A..........  72
Cast_NTS_long_LG3_30732465 ..................C..........C......--A..........-..............A..........  72
Cast_NTS_long_LG3_30733430 ..................C..........C......--A..........-..............A..........  72
Cast_NTS_long_LG3_30734973 ..................C..........C......--A..........-..............A..........  72
Cast_NTS_long_LG3_28565244 ...........G......C..........C......--A..........-.......G.................  72
Cast_NTS_long_LG3_30728025 ...........G......C..........C......--A..........-.......G.................  72
Cast_NTS_long_LG3_30729762 ...........G......C..........C......--A..........-.......G.................  72
Cast_NTS_long_LG3_30461572 ...........G.....TC..........C......--.....C....-.........................  72
Cast_NTS_long_LG3_30728411 ...........G.....TC..........C......--.....C....-.........................  72
Cast_NTS_long_LG3_30731500 ...........G.....TC..........C......--.....C....-.........................  72
Cast_NTS_long_LG3_30735938 ...........G.....TC..........C......--.....C....-.........................  72
Cast_NTS_long_LG3_30736131 ...........G.....TC..........C......--.....C....-.........................  72
Cast_NTS_long_LG3_30736324 ...........G.....TC..........C......--.....C....-.........................  72
Cast_NTS_long_LG3_28562732 ..................C..........C......--...........-.............T...........  72
Cast_NTS_long_LG3_28563504 ..................C..........C......--A..........-.............T...........  72
Cast_NTS_long_LG3_28559645 ..................C..........C......--T..........-.........................  72
Cast_NTS_long_LG3_28562539 ..................C..........C......--T..........-.........................  72
Cast_NTS_long_LG3_28559838 .................TC..........C......--T..........-.........................  72
Cast_NTS_long_LG3_28557141 ...........G.....TC..........C......--T..........-.........................  72
Cast_NTS_long_LG3_28560610 ...........G.....TC..........C......--T..........-.........................  72
Cast_NTS_long_LG3_28563892 ...........G.....TC..........C......--T..........-.........................  72
Cast_NTS_long_LG3_28564665 ...........G.....TC..........C......--T..........-.........................  72
Cast_NTS_long_LG3_28564858 ...........G.....TC..........C......--T..........-.........................  72
Cast_NTS_long_LG3_28561575 -..........G.....TC..........C......--T..........-.........................  71
Cast_NTS_long_LG3_28558489 .................TC..........C......--T..........-.............T...........  72
Cast_NTS_long_LG3_28563698 .................TC..........C......--T..........-.............T...........  72
Cast_NTS_long_LG3_28564471 .................TC..........C......--T..........-.............T...........  72
Cast_NTS_long_LG3_28555984 .........A........C..........C......--.....C....-.........................  72
Cast_NTS_long_LG3_28559259 ..................C..........C......--...........-.C.......................  72
Cast_NTS_long_LG3_28560417 ..................C..........C......--...........-.C.......................  72
Cast_NTS_long_LG3_28561767 ..................C..........C......--...........-.C.......................  72
Cast_NTS_long_LG3_28555791 ..................C..........C......--...........-.C..T....................  72
Cast_NTS_long_LG3_28556370 ..................C..........C......--...........-.C..T....................  72
Cast_NTS_long_LG3_28556948 ..................C..........C......--...........-.C..T....................  72
Cast_NTS_long_LG3_28557525 ..................C..........C......--...........-.C..T....................  72
Cast_NTS_long_LG3_28558103 ..................C..........C......--...........-.C..T....................  72
Cast_NTS_long_LG3_28558873 ..................C..........C......--...........-.C..T....................  72
Cast_NTS_long_LG3_28559066 ..................C..........C......--...........-.C..T....................  72
Cast_NTS_long_LG3_28559452 ..................C..........C......--...........-.C..T....................  72
Cast_NTS_long_LG3_28560224 ..................C..........C......--...........-.C..T....................  72
Cast_NTS_long_LG3_28561960 ..................C..........C......--...........-.C..T....................  72
Cast_NTS_long_LG3_28562346 ..................C..........C......--...........-.C..T....................  72
Cast_NTS_long_LG3_28562925 ..................C..........C......--...........-.C..T....................  72
Cast_NTS_long_LG3_28563118 ..................C..........C......--...........-.C..T....................  72
Cast_NTS_long_LG3_28563311 ..................C..........C......--...........-.C..T....................  72
Cast_NTS_long_LG3_28564278 ..................C..........C......--...........-.C..T....................  72
Cast_NTS_long_LG3_28562153 ..................C..........C......--...........-.C..T..............T.....  72
Cast_NTS_long_LG3_30461766 -------------------..........C......--...........-.C..T..............T.C...  53
Cast_NTS_long_LG3_28556177 ..................C..........C.....T--...........-.C..T....................  72
Cast_NTS_long_LG3_28556755 ..................C..........C.....T--...........-.C..T....................  72
Cast_NTS_long_LG3_28555212 ...........G.....TC..........C......--.....C....-.C..T....................  72
Cast_NTS_long_LG3_28555405 ...........G.....TC.C........C......--.....C....-.C..T....................  72
Cast_NTS_long_LG3_28946643 ............A................C......--...........-.........................  72
Cast_NTS_long_LG3_28946450 ............A................C......--....A......-.........................  72
Cast_NTS_long_LG3_30461379 ............A...ATC..........C......--...........-.C..T..............TT....  72
Cast_NTS_long_LG6_12284052 .....A.....T.....TC..........C......--...........-.........................  72
Cast_NTS_long_LG3_30734009 ..................C..........C......--A..........-..........C........TT....  72
Cast_NTS_long_LG3_30728797 ..................C..........C......--A..........-.C........C........TT....  72
Cast_NTS_long_LG3_30730534 ..................C..........C......--A..........-.C........C........TT....  72
Cast_NTS_long_LG3_30731886 ..................C..........C......--A..........-.C........C........TT....  72
Cast_NTS_long_LG3_30732272 ..................C..........C......--A..........-.C........C........TT....  72
Cast_NTS_long_LG3_30732851 ..................C..........C......--A..........-.C........C........TT....  72
Cast_NTS_long_LG3_30733237 ..................C..........C......--A..........-.C........C........TT....  72
Cast_NTS_long_LG3_30734587 ..................C..........C......--A..........-.C........C........TT....  72
Cast_NTS_long_LG3_28565051 .......G...G......C..........C......--...........-.C........C........TT....  72
Cast_NTS_long_LG3_30728218 .......G...G......C..........C......--...........-.C........C........TT....  72
Cast_NTS_long_LG3_30729955 .......G...G......C..........C......--...........-.C........C........TT....  72
Cast_NTS_long_LG3_30731307 .......G...G......C..........C......--...........-.C..G.....C........TT....  72
Cast_NTS_long_LG3_30731693 .......G...G......C..........C......--...........-.C..G.....C........TT....  72
Cast_NTS_long_LG3_30732658 .......G...G......C..........C......--...........-.C..G.....C........TT....  72
Cast_NTS_long_LG3_30735359 .......G...G......C..........C......--...........-.C..G.....C........TT....  72
Cast_NTS_long_LG3_30735745 .......G...G......C..........C......--...........-.C..G.....C........TT....  72
Cast_NTS_long_LG2_1470846  .................TC..........--C..G.--...........-.........................  70
Cast_NTS_long_LG2_1514133  .................TC..........--C....--...........-.C..T....................  70
Cast_NTS_long_LG2_352935   .................TC.........T--C....--...........-.C..T....................  70
Cast_NTS_long_LG3_28565437 -............G...TCC...--...T-CA.A..--...T.......-.C..T....................  68
Cast_NTS_long_LG3_30727836 -............G...TCC...--...T-CA.A..--...T.......-.C..T....................  68
```

**B**

```
Consensus                              TGAGGCAACGGCACGCGGTGTTCCCAAGCAAATTGTAAATTTGTCAATTGTAAATTTTGTCGTCTTCAAAAGAGATTAAC-TAGAAAGTAATAAGAATA-TTTAGAATGTAAGTGCAAAAAAAAGGA  125
TfSat09_F1cl156x122L_fLG3_26910978     ---.............................................................................-..T...A...........-...........................  122
TfSat09_F1cl156x122L_fLG3_26911223     ---.............................................................................-..T...A...........-...........................  122
TfSat09_F1cl156x122L_fLG3_26911345     ---.............................................................................-..T...A...........-...........................  122
TfSat09_F1cl156x122L_fLG3_26920906     ---.............................................................................-..T...A...........-...........................  122
TfSat09_F1cl156x122L_fLG3_26921151     ---.............................................................................-..T...A...........-...........................  122
TfSat09_F1cl156x122L_fLG3_26921520     ---.............................................................................-..T...A...........-...........................  122
TfSat09_F1cl156x122L_fLG3_26921642     ---.............................................................................-..T...A...........-...........................  122
TfSat09_F1cl156x122L_fLG3_26922132     ---.............................................................................-..T...A...........-...........................  122
TfSat09_F1cl156x122L_fLG3_26922254     ---.............................................................................-..T...A...........-...........................  122
TfSat09_F1cl156x122L_fLG3_26922744     ---.............................................................................-..T...A...........-...........................  122
TfSat09_F1cl156x122L_fLG3_26922866     ---.............................................................................-..T...A...........-...........................  122
TfSat09_F1cl156x122L_fLG3_26927019     ---.............................................................................-..T...A...........-...........................  122
TfSat09_F1cl156x122L_fLG3_26927141     ---.............................................................................-..T...A...........-...........................  122
TfSat09_F1cl156x122L_fLG3_26927263     ---.............................................................................-..T...A...........-...........................  122
TfSat09_F1cl156x122L_fLG3_26933031     ---.............................................................................-..T...A...........-...........................  122
TfSat09_F1cl156x122L_fLG3_26933543     ---.............................................................................-..T...A...........-...........................  122
TfSat09_F1cl156x122L_fLG3_26935186     ---.............................................................................-..T...A...........-...........................  122
TfSat09_F1cl156x122L_fLG3_26941375     ---.............................................................................-..T...A...........-...........................  122
TfSat09_F1cl156x122L_fLG3_26941497     ---.............................................................................-..T...A...........-...........................  122
TfSat09_F1cl156x122L_fLG3_26941619     ---.............................................................................-..T...A...........-...........................  122
TfSat09_F1cl156x122L_fLG3_26941741     ---.............................................................................-..T...A...........-...........................  122
TfSat09_F1cl156x122L_fLG3_26941863     ---.............................................................................-..T...A...........-...........................  122
TfSat09_F1cl156x122L_fLG3_26941985     ---.............................................................................-..T...A...........-...........................  122
TfSat09_F1cl156x122L_fLG3_26966742     ---.............................................................................-..T...A...........-...........................  122
TfSat09_F1cl156x122L_fLG3_26982539     ---.............................................................................-..T...A...........-...........................  122
TfSat09_F1cl156x122L_fLG3_27010322     ---.............................................................................-..T...A...........-...........................  122
TfSat09_F1cl156x122L_fLG3_27018749     ---.............................................................................-..T...A...........-...........................  122
TfSat09_F1cl156x122L_fLG3_27386570     ---.............................................................................-..T...A...........-...........................  122
TfSat09_F1cl156x122L_fLG3_27387132     ---.............................................................................-..T...A...........-...........................  122
TfSat09_F1cl156x122L_fLG3_27387572     ---.............................................................................-..T...A...........-...........................  122
TfSat09_F1cl156x122L_fLG3_27388060     ---.............................................................................-..T...A...........-...........................  122
TfSat09_F1cl156x122L_fLG3_27388426     ---.............................................................................-..T...A...........-...........................  122
TfSat09_F1cl156x122L_fLG3_27389355     ---.............................................................................-..T...A...........-...........................  122
TfSat09_F1cl156x122L_fLG3_27389599     ---.............................................................................-..T...A...........-...........................  122
TfSat09_F1cl156x122L_fLG3_27389721     ---.............................................................................-..T...A...........-...........................  122
TfSat09_F1cl156x122L_fLG3_27389965     ---.............................................................................-..T...A...........-...........................  122
TfSat09_F1cl156x122L_fLG3_27390087     ---.............................................................................-..T...A...........-...........................  122
TfSat09_F1cl156x122L_fLG3_27390331     ---.............................................................................-..T...A...........-...........................  122
TfSat09_F1cl156x122L_fLG3_27390575     ---.............................................................................-..T...A...........-...........................  122
TfSat09_F1cl156x122L_fLG3_27390697     ---.............................................................................-..T...A...........-...........................  122
TfSat09_F1cl156x122L_fLG3_27391185     ---.............................................................................-..T...A...........-...........................  122
TfSat09_F1cl156x122L_fLG3_27391429     ---.............................................................................-..T...A...........-...........................  122
TfSat09_F1cl156x122L_fLG3_27391551     ---.............................................................................-..T...A...........-...........................  122
TfSat09_F1cl156x122L_fLG3_27391917     ---.............................................................................-..T...A...........-...........................  122
TfSat09_F1cl156x122L_fLG3_27392527     ---.............................................................................-..T...A...........-...........................  122
TfSat09_F1cl156x122L_fLG3_27392649     ---.............................................................................-..T...A...........-...........................  122
TfSat09_F1cl156x122L_fLG3_27393015     ---.............................................................................-..T...A...........-...........................  122
TfSat09_F1cl156x122L_fLG3_27393894     ---.............................................................................-..T...A...........-...........................  122
TfSat09_F1cl156x122L_fLG3_27394138     ---.............................................................................-..T...A...........-...........................  122
TfSat09_F1cl156x122L_fLG3_27394260     ---.............................................................................-..T...A...........-...........................  122
TfSat09_F1cl156x122L_fLG3_27394504     ---.............................................................................-..T...A...........-...........................  122
TfSat09_F1cl156x122L_fLG3_27394626     ---.............................................................................-..T...A...........-...........................  122
TfSat09_F1cl156x122L_fLG3_27394748     ---.............................................................................-..T...A...........-...........................  122
TfSat09_F1cl156x122L_fLG3_27395187     ---.............................................................................-..T...A...........-...........................  122
TfSat09_F1cl156x122L_fLG3_27395506     ---.............................................................................-..T...A...........-...........................  122
TfSat09_F1cl156x122L_fLG3_27395628     ---.............................................................................-..T...A...........-...........................  122
TfSat09_F1cl156x122L_fLG3_27395872     ---.............................................................................-..T...A...........-...........................  122
TfSat09_F1cl156x122L_fLG3_27396311     ---.............................................................................-..T...A...........-...........................  122
TfSat09_F1cl156x122L_fLG3_27397068     ---.............................................................................-..T...A...........-...........................  122
TfSat09_F1cl156x122L_fLG3_27397385     ---.............................................................................-..T...A...........-...........................  122
TfSat09_F1cl156x122L_fLG3_27397702     ---.............................................................................-..T...A...........-...........................  122
TfSat09_F1cl156x122L_fLG3_27398019     ---.............................................................................-..T...A...........-...........................  122
TfSat09_F1cl156x122L_fLG3_27398850     ---.............................................................................-..T...A...........-...........................  122
TfSat09_F1cl156x122L_fLG3_27399167     ---.............................................................................-..T...A...........-...........................  122
TfSat09_F1cl156x122L_fLG3_27400119     ---.............................................................................-..T...A...........-...........................  122
TfSat09_F1cl156x122L_fLG3_27401535     ---.............................................................................-..T...A...........-...........................  122
TfSat09_F1cl156x122L_fLG3_27401852     ---.............................................................................-..T...A...........-...........................  122
```

| | | |
|---|---|---|
| TfSat09_F1cl156x122L_fLG3_27402658 | ---.............................................................................-..T...A...........-........................... | 122 |
| TfSat09_F1cl156x122L_fLG3_27403292 | ---.............................................................................-..T...A...........-........................... | 122 |
| TfSat09_F1cl156x122L_fLG3_27403731 | ---.............................................................................-..T...A...........-........................... | 122 |
| TfSat09_F1cl156x122L_fLG3_27425928 | ---.............................................................................-..T...A...........-........................... | 122 |
| TfSat09_F1cl156x122L_fLG3_26911100 | ---.............................................................................-..T...A...........-........................--- | 119 |
| TfSat09_F1cl156x122L_fLG3_26922009 | ---.............................................................................-..T...A...........-........................--- | 119 |
| TfSat09_F1cl156x122L_fLG3_26922498 | ---.............................................................................-..T...A...........-........................--- | 119 |
| TfSat09_F1cl156x122L_fLG3_26922621 | ---.............................................................................-..T...A...........-........................--- | 119 |
| TfSat09_F1cl156x122L_fLG3_26956685 | ---.............................................................................-..T...A...........-........................--- | 119 |
| TfSat09_F1cl156x122L_fLG3_27398146 | ---.............................................................................-..T...A...........-........................--- | 119 |
| TfSat09_F1cl156x122L_fLG3_27402417 | ---.............................................................................-..T...A...........-........................--- | 119 |
| TfSat09_F1cl156x122L_fLG3_27404176 | ---.............................................................................-..T...A...........-........................--- | 119 |
| TfSat09_F1cl156x122L_fLG3_27405502 | ---.............................................................................-..T...A...........-........................--- | 119 |
| TfSat09_F1cl156x122L_fLG3_27406707 | ---.............................................................................-..T...A...........-........................--- | 119 |
| TfSat09_F1cl156x122L_fLG3_27408477 | ---.............................................................................-..T...A...........-........................--- | 119 |
| TfSat09_F1cl156x122L_fLG3_27408797 | ---.............................................................................-..T...A...........-........................--- | 119 |
| TfSat09_F1cl156x122L_fLG3_27409362 | ---.............................................................................-..T...A...........-........................--- | 119 |
| TfSat09_F1cl156x122L_fLG3_27410124 | ---.............................................................................-..T...A...........-........................--- | 119 |
| TfSat09_F1cl156x122L_fLG3_27412383 | ---.............................................................................-..T...A...........-........................--- | 119 |
| TfSat09_F1cl156x122L_fLG3_27413146 | ---.............................................................................-..T...A...........-........................--- | 119 |
| TfSat09_F1cl156x122L_fLG3_27414670 | ---.............................................................................-..T...A...........-........................--- | 119 |
| TfSat09_F1cl156x122L_fLG3_27416759 | ---.............................................................................-..T...A...........-........................--- | 119 |
| TfSat09_F1cl156x122L_fLG3_27417448 | ---.............................................................................-..T...A...........-........................--- | 119 |
| TfSat09_F1cl156x122L_fLG3_27418211 | ---.............................................................................-..T...A...........-........................--- | 119 |
| TfSat09_F1cl156x122L_fLG3_27418974 | ---.............................................................................-..T...A...........-........................--- | 119 |
| TfSat09_F1cl156x122L_fLG3_27420178 | ---.............................................................................-..T...A...........-........................--- | 119 |
| TfSat09_F1cl156x122L_fLG3_27420942 | ---.............................................................................-..T...A...........-........................--- | 119 |
| TfSat09_F1cl156x122L_fLG3_27421263 | ---.............................................................................-..T...A...........-........................--- | 119 |
| TfSat09_F1cl156x122L_fLG3_27423032 | ---.............................................................................-..T...A...........-........................--- | 119 |
| TfSat09_F1cl156x122L_fLG3_27423795 | ---.............................................................................-..T...A...........-........................--- | 119 |
| TfSat09_F1cl156x122L_fLG3_27424558 | ---.............................................................................-..T...A...........-........................--- | 119 |
| TfSat09_F1cl156x122L_fLG3_27425563 | ---.............................................................................-..T...A...........-........................--- | 119 |
| TfSat09_F1cl156x122L_fLG3_27392161 | ---.............................................................................-..T...A...........-........................T.. | 122 |
| TfSat09_F1cl156x122L_fLG3_26921887 | ---...........................................G.................................-..T...A...........-........................... | 122 |
| TfSat09_F1cl156x122L_fLG3_26922376 | ---...........................................G.................................-..T...A...........-........................... | 122 |
| TfSat09_F1cl156x122L_fLG3_26922988 | ---...........................................G.................................-..T...A...........-........................... | 122 |
| TfSat09_F1cl156x122L_fLG3_26923233 | ---...........................................G.................................-..T...A...........-........................... | 122 |
| TfSat09_F1cl156x122L_fLG3_26923477 | ---...........................................G.................................-..T...A...........-........................... | 122 |
| TfSat09_F1cl156x122L_fLG3_26923721 | ---...........................................G.................................-..T...A...........-........................... | 122 |
| TfSat09_F1cl156x122L_fLG3_26923965 | ---...........................................G.................................-..T...A...........-........................... | 122 |
| TfSat09_F1cl156x122L_fLG3_26924331 | ---...........................................G.................................-..T...A...........-........................... | 122 |
| TfSat09_F1cl156x122L_fLG3_26924453 | ---...........................................G.................................-..T...A...........-........................... | 122 |
| TfSat09_F1cl156x122L_fLG3_26924575 | ---...........................................G.................................-..T...A...........-........................... | 122 |
| TfSat09_F1cl156x122L_fLG3_26924697 | ---...........................................G.................................-..T...A...........-........................... | 122 |
| TfSat09_F1cl156x122L_fLG3_26924941 | ---...........................................G.................................-..T...A...........-........................... | 122 |
| TfSat09_F1cl156x122L_fLG3_26925185 | ---...........................................G.................................-..T...A...........-........................... | 122 |
| TfSat09_F1cl156x122L_fLG3_26925430 | ---...........................................G.................................-..T...A...........-........................... | 122 |
| TfSat09_F1cl156x122L_fLG3_26925674 | ---...........................................G.................................-..T...A...........-........................... | 122 |
| TfSat09_F1cl156x122L_fLG3_26925919 | ---...........................................G.................................-..T...A...........-........................... | 122 |
| TfSat09_F1cl156x122L_fLG3_26926163 | ---...........................................G.................................-..T...A...........-........................... | 122 |
| TfSat09_F1cl156x122L_fLG3_26926531 | ---...........................................G.................................-..T...A...........-........................... | 122 |
| TfSat09_F1cl156x122L_fLG3_26926775 | ---...........................................G.................................-..T...A...........-........................... | 122 |
| TfSat09_F1cl156x122L_fLG3_26940742 | ---...........................................G.................................-..T...A...........-........................... | 122 |
| TfSat09_F1cl156x122L_fLG3_26962802 | ---...........................................G.................................-..T...A...........-........................... | 122 |
| TfSat09_F1cl156x122L_fLG3_26991364 | ---...........................................G.................................-..T...A...........-........................... | 122 |
| TfSat09_F1cl156x122L_fLG3_26991730 | ---...........................................G.................................-..T...A...........-........................... | 122 |
| TfSat09_F1cl156x122L_fLG3_26992776 | ---...........................................G.................................-..T...A...........-........................... | 122 |
| TfSat09_F1cl156x122L_fLG3_26995001 | ---...........................................G.................................-..T...A...........-........................... | 122 |
| TfSat09_F1cl156x122L_fLG3_26995734 | ---...........................................G.................................-..T...A...........-........................... | 122 |
| TfSat09_F1cl156x122L_fLG3_26996100 | ---...........................................G.................................-..T...A...........-........................... | 122 |
| TfSat09_F1cl156x122L_fLG3_27002413 | ---...........................................G.................................-..T...A...........-........................... | 122 |
| TfSat09_F1cl156x122L_fLG3_27002535 | ---...........................................G.................................-..T...A...........-........................... | 122 |
| TfSat09_F1cl156x122L_fLG3_27002901 | ---...........................................G.................................-..T...A...........-........................... | 122 |
| TfSat09_F1cl156x122L_fLG3_27003267 | ---...........................................G.................................-..T...A...........-........................... | 122 |
| TfSat09_F1cl156x122L_fLG3_27010078 | ---...........................................G.................................-..T...A...........-........................... | 122 |
| TfSat09_F1cl156x122L_fLG3_26926408 | ---...........................................G.................................-..T...A...........-........................--- | 119 |
| TfSat09_F1cl156x122L_fLG3_26995244 | ---...........................................G.................................-..T...A...........-........................--- | 119 |
| TfSat09_F1cl156x122L_fLG3_26995611 | ---...........................................G.................................-..T...A...........-........................--- | 119 |
| TfSat09_F1cl156x122L_fLG3_26940621 | ---...........................................G.................................-..T...A...........-.......................---- | 118 |
| TfSat09_F1cl156x122L_fLG3_26962681 | ---...........................................G.................................-..T...A...........-.......................---- | 118 |
| TfSat09_F1cl156x122L_fLG3_26923843 | ---...........A.................................................................-..T...A...........-........................... | 122 |

```
TfSat09_F1cl156x122L_fLG3_26924819    ---...........A.................................................................-..T...A...........-...........................  122
TfSat09_F1cl156x122L_fLG3_26926897    ---...........A.................................................................-..T...A...........-...........................  122
TfSat09_F1cl156x122L_fLG3_26923110    ---...........A.................................................................-..T...A...........-........................---  119
TfSat09_F1cl156x122L_fLG3_26925307    ---...........A.................................................................-..T...A...........-........................---  119
TfSat09_F1cl156x122L_fLG3_26925796    ---...........A.................................................................-..T...A...........-........................---  119
TfSat09_F1cl156x122L_fLG3_26926285    ---...........A.................................................................-..T...A...........-........................---  119
TfSat09_F1cl156x122L_fLG3_26923355    ---...........A...............................G.................................-..T...A...........-...........................  122
TfSat09_F1cl156x122L_fLG3_26923599    ---...........A...............................G.................................-..T...A...........-...........................  122
TfSat09_F1cl156x122L_fLG3_26924209    ---...........A...............................G.................................-..T...A...........-...........................  122
TfSat09_F1cl156x122L_fLG3_26925063    ---...........A...............................G.................................-..T...A...........-...........................  122
TfSat09_F1cl156x122L_fLG3_26925552    ---...........A...............................G.................................-..T...A...........-...........................  122
TfSat09_F1cl156x122L_fLG3_26926041    ---...........A...............................G.................................-..T...A...........-...........................  122
TfSat09_F1cl156x122L_fLG3_26926653    ---...........A...............................G.................................-..T...A...........-...........................  122
TfSat09_F1cl156x122L_fLG3_27388670    ---..........................................................................G..-..T...A...........-...........................  122
TfSat09_F1cl156x122L_fLG3_27393259    ---..........................................................................G..-..T...A...........-...........................  122
TfSat09_F1cl156x122L_fLG3_27388918    ---..........................................................................G..-..T...A...........-........................---  119
TfSat09_F1cl156x122L_fLG3_26927507    ---.......................................................A.....................-..T...A...........-...........................  122
TfSat09_F1cl156x122L_fLG3_27010688    ---...................................................................G.........-..T...A...........-...........................  122
TfSat09_F1cl156x122L_fLG3_27010810    ---.............................................................................-..T...A...........-....A......................  122
TfSat09_F1cl156x122L_fLG3_27018871    ---.........................................................T...................-..T...A...........-...........................  122
TfSat09_F1cl156x122L_fLG3_27390819    ---....................................................................A........-..T...A...........-...........................  122
TfSat09_F1cl156x122L_fLG3_27391673    ---....................................................................A........-..T...A...........-...........................  122
TfSat09_F1cl156x122L_fLG3_27392039    ---..........................G..................................................-..T...A...........-...........................  122
TfSat09_F1cl156x122L_fLG3_27395750    ---....................................C........................................-..T...A...........-...........................  122
TfSat09_F1cl156x122L_fLG3_27396555    ---...............................................C.............................-..T...A...........-...........................  122
TfSat09_F1cl156x122L_fLG3_27399411    ---...............................................C.............................-..T...A...........-...........................  122
TfSat09_F1cl156x122L_fLG3_27400241    ---...............................................C.............................-..T...A...........-...........................  122
TfSat09_F1cl156x122L_fLG3_27402096    ---...............................................C.............................-..T...A...........-...........................  122
TfSat09_F1cl156x122L_fLG3_27402780    ---...............................................C.............................-..T...A...........-...........................  122
TfSat09_F1cl156x122L_fLG3_27425118    ---...............................................C.............................-..T...A...........-...........................  122
TfSat09_F1cl156x122L_fLG3_27425806    ---.............................................................................-..T...A.......G...-...........................  122
TfSat09_F1cl156x122L_fLG3_27389477    ---.............................................................................-..T...A.......T...-...........................  122
TfSat09_F1cl156x122L_fLG3_27394016    ---.............................................................................-..T...A.......T...-...........................  122
TfSat09_F1cl156x122L_fLG3_27396433    ---.............................................................................-..T...A.......T...-...........................  122
TfSat09_F1cl156x122L_fLG3_27399289    ---.............................................................................-..T...A.......T...-...........................  122
TfSat09_F1cl156x122L_fLG3_27401974    ---.............................................................................-..T...A.......T...-...........................  122
TfSat09_F1cl156x122L_fLG3_27402536    ---.............................................................................-..T...A.......T...-...........................  122
TfSat09_F1cl156x122L_fLG3_27424996    ---.............................................................................-..T...A.......T...-...........................  122
TfSat09_F1cl156x122L_fLG3_26956808    ---.............................................................................-..T...A...........-....C......................  122
TfSat09_F1cl156x122L_fLG3_27389843    ---......................................................................A.....T-..T...A...........-...........................  122
TfSat09_F1cl156x122L_fLG3_27394382    ---......................................................................A.....T-..T...A...........-...........................  122
TfSat09_F1cl156x122L_fLG3_26921028    ---............................................................................G-..T...A...........-........................---  119
TfSat09_F1cl156x122L_fLG3_26921273    ---............................................................................G-..T...A...........-........................---  119
TfSat09_F1cl156x122L_fLG3_27010566    ---........................T....................................................-..T...A...........-...........................  122
TfSat09_F1cl156x122L_fLG3_27387938    ---..............T..............................................................-..T...A...........-...........................  122
TfSat09_F1cl156x122L_fLG3_26921396    ---.............................................................................-..T...A.....T.....-........................---  119
TfSat09_F1cl156x122L_fLG3_26903971    ---.............................................................................-A.T...A...........-...........................  122
TfSat09_F1cl156x122L_fLG3_26904460    ---.............................................................................-A.T...A...........-...........................  122
TfSat09_F1cl156x122L_fLG3_26957247    ---.............................................................................-G.T...A...........-...........................  122
TfSat09_F1cl156x122L_fLG3_26904582    ---...........................................G.................................-A.T...A...........-...........................  122
TfSat09_F1cl156x122L_fLG3_26904826    ---...........................................G.................................-A.T...A...........-...........................  122
TfSat09_F1cl156x122L_fLG3_26905070    ---...........................................G.................................-A.T...A...........-...........................  122
TfSat09_F1cl156x122L_fLG3_26905314    ---...........................................G.................................-A.T...A...........-...........................  122
TfSat09_F1cl156x122L_fLG3_26905558    ---...........................................G.................................-A.T...A...........-...........................  122
TfSat09_F1cl156x122L_fLG3_27009834    ---...........................................G.................................-A.T...A...........-...........................  122
TfSat09_F1cl156x122L_fLG3_27009956    ---...........................................G.................................-A.T...A...........-...........................  122
TfSat09_F1cl156x122L_fLG3_27011054    ---...........................................G.................................-A.T...A...........-...........................  122
TfSat09_F1cl156x122L_fLG3_26904704    ---...........................................G.................................-A.T...AC..........-...........................  122
TfSat09_F1cl156x122L_fLG3_26904948    ---..................................G........G.................................-A.T...A...........-...........................  122
TfSat09_F1cl156x122L_fLG3_26905436    ---...........................................G...........A.....................-A.T...A...........-...........................  122
TfSat09_F1cl156x122L_fLG3_26905192    ---...........................................G...............A.................-A.T...A...........-...........................  122
TfSat09_F1cl156x122L_fLG3_26927629    ---.....A.......................................................................-..T...A...........-...........................  122
TfSat09_F1cl156x122L_fLG3_27387816    ---.....G.......................................................................-..T...A...........-...........................  122
TfSat09_F1cl156x122L_fLG3_27392405    ---.....G.......................................................................-..T...A...........-...........................  122
TfSat09_F1cl156x122L_fLG3_27009322    ---.....A.......................................................................-A.T...A...........-...........................  122
TfSat09_F1cl156x122L_fLG3_27386692    ---.............................................................................-..T...C...........-...........................  122
TfSat09_F1cl156x122L_fLG3_27387254    ---.............................................................................-..T...C...........-...........................  122
TfSat09_F1cl156x122L_fLG3_27388548    ---.............................................................................-..T...C...........-...........................  122
TfSat09_F1cl156x122L_fLG3_27388792    ---.............................................................................-..T...C...........-...........................  122
TfSat09_F1cl156x122L_fLG3_27389037    ---.............................................................................-..T...C...........-...........................  122
TfSat09_F1cl156x122L_fLG3_27392771    ---.............................................................................-..T...C...........-...........................  122
```

```
TfSat09_F1cl156x122L_fLG3_27393137    ---.............................................................................-..T...C...........-...........................  122
TfSat09_F1cl156x122L_fLG3_27393381    ---.............................................................................-..T...C...........-...........................  122
TfSat09_F1cl156x122L_fLG3_27395994    ---.............................................................................-..T...C...........-...........................  122
TfSat09_F1cl156x122L_fLG3_27404490    ---.............................................................................-..T...C...........-...........................  122
TfSat09_F1cl156x122L_fLG3_27405178    ---.............................................................................-..T...C...........-...........................  122
TfSat09_F1cl156x122L_fLG3_27405818    ---.............................................................................-..T...C...........-...........................  122
TfSat09_F1cl156x122L_fLG3_27406260    ---.............................................................................-..T...C...........-...........................  122
TfSat09_F1cl156x122L_fLG3_27407023    ---.............................................................................-..T...C...........-...........................  122
TfSat09_F1cl156x122L_fLG3_27407588    ---.............................................................................-..T...C...........-...........................  122
TfSat09_F1cl156x122L_fLG3_27408030    ---.............................................................................-..T...C...........-...........................  122
TfSat09_F1cl156x122L_fLG3_27408916    ---.............................................................................-..T...C...........-...........................  122
TfSat09_F1cl156x122L_fLG3_27409678    ---.............................................................................-..T...C...........-...........................  122
TfSat09_F1cl156x122L_fLG3_27410440    ---.............................................................................-..T...C...........-...........................  122
TfSat09_F1cl156x122L_fLG3_27410882    ---.............................................................................-..T...C...........-...........................  122
TfSat09_F1cl156x122L_fLG3_27411201    ---.............................................................................-..T...C...........-...........................  122
TfSat09_F1cl156x122L_fLG3_27411323    ---.............................................................................-..T...C...........-...........................  122
TfSat09_F1cl156x122L_fLG3_27411568    ---.............................................................................-..T...C...........-...........................  122
TfSat09_F1cl156x122L_fLG3_27412010    ---.............................................................................-..T...C...........-...........................  122
TfSat09_F1cl156x122L_fLG3_27412699    ---.............................................................................-..T...C...........-...........................  122
TfSat09_F1cl156x122L_fLG3_27413462    ---.............................................................................-..T...C...........-...........................  122
TfSat09_F1cl156x122L_fLG3_27413904    ---.............................................................................-..T...C...........-...........................  122
TfSat09_F1cl156x122L_fLG3_27414986    ---.............................................................................-..T...C...........-...........................  122
TfSat09_F1cl156x122L_fLG3_27415428    ---.............................................................................-..T...C...........-...........................  122
TfSat09_F1cl156x122L_fLG3_27416312    ---.............................................................................-..T...C...........-...........................  122
TfSat09_F1cl156x122L_fLG3_27417075    ---.............................................................................-..T...C...........-...........................  122
TfSat09_F1cl156x122L_fLG3_27417764    ---.............................................................................-..T...C...........-...........................  122
TfSat09_F1cl156x122L_fLG3_27418527    ---.............................................................................-..T...C...........-...........................  122
TfSat09_F1cl156x122L_fLG3_27419290    ---.............................................................................-..T...C...........-...........................  122
TfSat09_F1cl156x122L_fLG3_27419731    ---.............................................................................-..T...C...........-...........................  122
TfSat09_F1cl156x122L_fLG3_27420494    ---.............................................................................-..T...C...........-...........................  122
TfSat09_F1cl156x122L_fLG3_27421579    ---.............................................................................-..T...C...........-...........................  122
TfSat09_F1cl156x122L_fLG3_27422021    ---.............................................................................-..T...C...........-...........................  122
TfSat09_F1cl156x122L_fLG3_27422143    ---.............................................................................-..T...C...........-...........................  122
TfSat09_F1cl156x122L_fLG3_27422585    ---.............................................................................-..T...C...........-...........................  122
TfSat09_F1cl156x122L_fLG3_27423348    ---.............................................................................-..T...C...........-...........................  122
TfSat09_F1cl156x122L_fLG3_27424111    ---.............................................................................-..T...C...........-...........................  122
TfSat09_F1cl156x122L_fLG3_27414223    ---...........................................................C.................-..T...C...........-...........................  122
TfSat09_F1cl156x122L_fLG3_27415870    ---...........................................................C.................-..T...C...........-...........................  122
TfSat09_F1cl156x122L_fLG3_27388182    ---.............................................................................-..T...C.........A.-...........................  122
TfSat09_F1cl156x122L_fLG3_27390209    ---..........A..................................................................-..T...A...........-...........................  122
TfSat09_F1cl156x122L_fLG3_27390453    ---..........A..................................................................-..T...A...........-...........................  122
TfSat09_F1cl156x122L_fLG3_27390941    ---..........A..................................................................-..T...A...........-...........................  122
TfSat09_F1cl156x122L_fLG3_27391063    ---..........A..................................................................-..T...A...........-...........................  122
TfSat09_F1cl156x122L_fLG3_27391795    ---..........A..................................................................-..T...A...........-...........................  122
TfSat09_F1cl156x122L_fLG3_27394870    ---..........A..................................................................-..T...A...........-...........................  122
TfSat09_F1cl156x122L_fLG3_27387694    ---..........A.....................C............................................-..T...A...........-...........................  122
TfSat09_F1cl156x122L_fLG3_27388304    ---..........A.....................C............................................-..T...A...........-...........................  122
TfSat09_F1cl156x122L_fLG3_27392283    ---..........A.....................C............................................-..T...A...........-...........................  122
TfSat09_F1cl156x122L_fLG3_27392893    ---..........A.....................C............................................-..T...A...........-...........................  122
TfSat09_F1cl156x122L_fLG3_27391307    ---..........A.............A....................................................-..T...A...........-...........................  122
TfSat09_F1cl156x122L_fLG3_27386448    ---..........A.......................................T..........................-..T...A...........-...........................  122
TfSat09_F1cl156x122L_fLG3_27387010    ---..........A.......................................T..........................-..T...A...........-...........................  122
TfSat09_F1cl156x122L_fLG3_27403609    ---............................................................T......GA........-..T...A...........-...........................  122
TfSat09_F1cl156x122L_fLG3_27404048    ---............................................................T......GA........-..T...A...........-...........................  122
TfSat09_F1cl156x122L_fLG3_27424874    ---............................................................T......GA........-..T...A...........-...........................  122
TfSat09_F1cl156x122L_fLG3_27425435    ---............................................................T......GA........-..T...A...........-...........................  122
TfSat09_F1cl156x122L_fLG3_26927385    ---.....A......................................................T......GA........-..T...A...........-...........................  122
TfSat09_F1cl156x122L_fLG3_26924087    ---...........................................G...................A.............-..T...A...........-...........................  122
TfSat09_F1cl156x122L_fLG3_26965277    ---.......C...................................G.................................-..T...A...........-...........................  122
TfSat09_F1cl156x122L_fLG3_26972887    ---.......C...................................G.................................-..T...A...........-...........................  122
TfSat09_F1cl156x122L_fLG3_26979278    ---.......C...................................G.................................-..T...A...........-...........................  122
TfSat09_F1cl156x122L_fLG3_26940500    ---.......C...................................G.................................-..T...A...........-.......................----  118
TfSat09_F1cl156x122L_fLG3_26962560    ---.......C...................................G.................................-..T...A...........-.......................----  118
TfSat09_F1cl156x122L_fLG3_26921764    ---...........A.................................................................-..T...A..........C-........................---  119
TfSat09_F1cl156x122L_fLG3_26903015    ---.............A...........................................T...................-..C...A.......G...-.......................----  118
TfSat09_F1cl156x122L_fLG3_27011370    ---.....A.......A...........................................T...................-..C...A.......G...-.......................----  118
TfSat09_F1cl156x122L_fLG3_27010200    ---.............................................................................-..................-...........................  122
TfSat09_F1cl156x122L_fLG3_27010444    ---.............................................................................-..................-...........................  122
TfSat09_F1cl156x122L_fLG3_27425687    ---.............................................................................-..................-........................---  119
TfSat09_var2_fLG3_27010264 (reversed) -------------------------------------------------------------------.............-..................-........................---   55
TfSat09_var2_fLG3_27010508 (reversed) -------------------------------------------------------------------.............-..................-........................---   55
```

```
TfSat09_var2_fLG3_27425687 (reversed) -------------------------------------------------------------------.............-..................-........................---   55
TfSat09_F1cl156x122L_fLG3_26957125    ---..............................................................................-..................-..............A............  122
TfSat09_F1cl156x122L_fLG3_27018627    ---..............................................................................-......A.......G...-...........................  122
TfSat09_F1cl156x122L_fLG3_26965399    ---..............................................................................-..............G...-...........................  122
TfSat09_F1cl156x122L_fLG3_26965644    ---..............................................................................-..............G...-...........................  122
TfSat09_F1cl156x122L_fLG3_26966254    ---..............................................................................-..............G...-...........................  122
TfSat09_F1cl156x122L_fLG3_26966499    ---..............................................................................-..............G...-...........................  122
TfSat09_F1cl156x122L_fLG3_26966864    ---..............................................................................-..............G...-...........................  122
TfSat09_F1cl156x122L_fLG3_26966986    ---..............................................................................-..............G...-...........................  122
TfSat09_F1cl156x122L_fLG3_26967108    ---..............................................................................-..............G...-...........................  122
TfSat09_F1cl156x122L_fLG3_26973009    ---..............................................................................-..............G...-...........................  122
TfSat09_F1cl156x122L_fLG3_26973254    ---..............................................................................-..............G...-...........................  122
TfSat09_F1cl156x122L_fLG3_26979400    ---..............................................................................-..............G...-...........................  122
TfSat09_F1cl156x122L_fLG3_26981442    ---..............................................................................-..............G...-...........................  122
TfSat09_F1cl156x122L_fLG3_26982661    ---..............................................................................-..............G...-...........................  122
TfSat09_F1cl156x122L_fLG3_26982783    ---..............................................................................-..............G...-...........................  122
TfSat09_F1cl156x122L_fLG3_26982905    ---..............................................................................-..............G...-...........................  122
TfSat09_F1cl156x122L_fLG3_26989050    ---..............................................................................-..............G...-...........................  122
TfSat09_F1cl156x122L_fLG3_26989903    ---..............................................................................-..............G...-...........................  122
TfSat09_F1cl156x122L_fLG3_26990269    ---..............................................................................-..............G...-...........................  122
TfSat09_F1cl156x122L_fLG3_26990877    ---..............................................................................-..............G...-...........................  122
TfSat09_F1cl156x122L_fLG3_26996343    ---..............................................................................-..............G...-...........................  122
TfSat09_F1cl156x122L_fLG3_27007353    ---..............................................................................-..............G...-...........................  122
TfSat09_F1cl156x122L_fLG3_26949814    ---..............................................................................-..............G...-........................---  119
TfSat09_F1cl156x122L_fLG3_26965521    ---..............................................................................-..............G...-........................---  119
TfSat09_F1cl156x122L_fLG3_26966376    ---..............................................................................-..............G...-........................---  119
TfSat09_F1cl156x122L_fLG3_26973131    ---..............................................................................-..............G...-........................---  119
TfSat09_F1cl156x122L_fLG3_26979522    ---..............................................................................-..............G...-........................---  119
TfSat09_F1cl156x122L_fLG3_26980466    ---..............................................................................-..............G...-........................---  119
TfSat09_F1cl156x122L_fLG3_26980589    ---..............................................................................-..............G...-........................---  119
TfSat09_F1cl156x122L_fLG3_26980833    ---..............................................................................-..............G...-........................---  119
TfSat09_F1cl156x122L_fLG3_26981198    ---..............................................................................-..............G...-........................---  119
TfSat09_F1cl156x122L_fLG3_26981806    ---..............................................................................-..............G...-........................---  119
TfSat09_F1cl156x122L_fLG3_26982050    ---..............................................................................-..............G...-........................---  119
TfSat09_F1cl156x122L_fLG3_26983515    ---..............................................................................-..............G...-........................---  119
TfSat09_F1cl156x122L_fLG3_26989414    ---..............................................................................-..............G...-........................---  119
TfSat09_F1cl156x122L_fLG3_26989780    ---..............................................................................-..............G...-........................---  119
TfSat09_F1cl156x122L_fLG3_26992532    ---..............................................................................-..............G...-........................---  119
TfSat09_F1cl156x122L_fLG3_26992898    ---..............................................................................-..............G...-........................---  119
TfSat09_F1cl156x122L_fLG3_26993509    ---..............................................................................-..............G...-........................---  119
TfSat09_F1cl156x122L_fLG3_26994069    ---..............................................................................-..............G...-........................---  119
TfSat09_F1cl156x122L_fLG3_26994511    ---..............................................................................-..............G...-........................---  119
TfSat09_F1cl156x122L_fLG3_26994634    ---..............................................................................-..............G...-........................---  119
TfSat09_F1cl156x122L_fLG3_26994878    ---..............................................................................-..............G...-........................---  119
TfSat09_F1cl156x122L_fLG3_27001313    ---..............................................................................-..............G...-........................---  119
TfSat09_F1cl156x122L_fLG3_27001437    ---..............................................................................-..............G...-........................---  119
TfSat09_F1cl156x122L_fLG3_27001560    ---..............................................................................-..............G...-........................---  119
TfSat09_F1cl156x122L_fLG3_27001683    ---..............................................................................-..............G...-........................---  119
TfSat09_F1cl156x122L_fLG3_27002169    ---..............................................................................-..............G...-........................---  119
TfSat09_F1cl156x122L_fLG3_27004069    ---..............................................................................-..............G...-........................---  119
TfSat09_F1cl156x122L_fLG3_27004192    ---..............................................................................-..............G...-........................---  119
TfSat09_F1cl156x122L_fLG3_27004788    ---..............................................................................-..............G...-........................---  119
TfSat09_F1cl156x122L_fLG3_27004912    ---..............................................................................-..............G...-........................---  119
TfSat09_F1cl156x122L_fLG3_27005036    ---..............................................................................-..............G...-........................---  119
TfSat09_F1cl156x122L_fLG3_27005281    ---..............................................................................-..............G...-........................---  119
TfSat09_F1cl156x122L_fLG3_27005646    ---..............................................................................-..............G...-........................---  119
TfSat09_F1cl156x122L_fLG3_27005770    ---..............................................................................-..............G...-........................---  119
TfSat09_F1cl156x122L_fLG3_27006378    ---..............................................................................-..............G...-........................---  119
TfSat09_F1cl156x122L_fLG3_27006986    ---..............................................................................-..............G...-........................---  119
TfSat09_F1cl156x122L_fLG3_27404616    ---..............................................................................-..............G...-........................---  119
TfSat09_F1cl156x122L_fLG3_27404739    ---..............................................................................-..............G...-........................---  119
TfSat09_F1cl156x122L_fLG3_27404862    ---..............................................................................-..............G...-........................---  119
TfSat09_F1cl156x122L_fLG3_27405944    ---..............................................................................-..............G...-........................---  119
TfSat09_F1cl156x122L_fLG3_27406386    ---..............................................................................-..............G...-........................---  119
TfSat09_F1cl156x122L_fLG3_27407149    ---..............................................................................-..............G...-........................---  119
TfSat09_F1cl156x122L_fLG3_27407272    ---..............................................................................-..............G...-........................---  119
TfSat09_F1cl156x122L_fLG3_27407714    ---..............................................................................-..............G...-........................---  119
TfSat09_F1cl156x122L_fLG3_27408156    ---..............................................................................-..............G...-........................---  119
TfSat09_F1cl156x122L_fLG3_27409042    ---..............................................................................-..............G...-........................---  119
TfSat09_F1cl156x122L_fLG3_27409804    ---..............................................................................-..............G...-........................---  119
TfSat09_F1cl156x122L_fLG3_27410566    ---..............................................................................-..............G...-........................---  119
```

```
TfSat09_F1cl156x122L_fLG3_27411449    ---.............................................................................-..............G...-........................---   119
TfSat09_F1cl156x122L_fLG3_27411694    ---.............................................................................-..............G...-........................---   119
TfSat09_F1cl156x122L_fLG3_27412136    ---.............................................................................-..............G...-........................---   119
TfSat09_F1cl156x122L_fLG3_27412259    ---.............................................................................-..............G...-........................---   119
TfSat09_F1cl156x122L_fLG3_27412825    ---.............................................................................-..............G...-........................---   119
TfSat09_F1cl156x122L_fLG3_27413588    ---.............................................................................-..............G...-........................---   119
TfSat09_F1cl156x122L_fLG3_27414349    ---.............................................................................-..............G...-........................---   119
TfSat09_F1cl156x122L_fLG3_27415112    ---.............................................................................-..............G...-........................---   119
TfSat09_F1cl156x122L_fLG3_27415554    ---.............................................................................-..............G...-........................---   119
TfSat09_F1cl156x122L_fLG3_27415996    ---.............................................................................-..............G...-........................---   119
TfSat09_F1cl156x122L_fLG3_27416438    ---.............................................................................-..............G...-........................---   119
TfSat09_F1cl156x122L_fLG3_27417201    ---.............................................................................-..............G...-........................---   119
TfSat09_F1cl156x122L_fLG3_27417324    ---.............................................................................-..............G...-........................---   119
TfSat09_F1cl156x122L_fLG3_27417890    ---.............................................................................-..............G...-........................---   119
TfSat09_F1cl156x122L_fLG3_27418653    ---.............................................................................-..............G...-........................---   119
TfSat09_F1cl156x122L_fLG3_27419416    ---.............................................................................-..............G...-........................---   119
TfSat09_F1cl156x122L_fLG3_27419857    ---.............................................................................-..............G...-........................---   119
TfSat09_F1cl156x122L_fLG3_27420620    ---.............................................................................-..............G...-........................---   119
TfSat09_F1cl156x122L_fLG3_27421705    ---.............................................................................-..............G...-........................---   119
TfSat09_F1cl156x122L_fLG3_27422269    ---.............................................................................-..............G...-........................---   119
TfSat09_F1cl156x122L_fLG3_27422711    ---.............................................................................-..............G...-........................---   119
TfSat09_F1cl156x122L_fLG3_27423474    ---.............................................................................-..............G...-........................---   119
TfSat09_F1cl156x122L_fLG3_27424237    ---.............................................................................-..............G...-........................---   119
TfSat09_F1cl156x122L_fLG3_27426054    ---.............................................................................-..............G...-........................---   119
TfSat09_F1cl156x122L_fLG3_26951391    ---.............................................................................-..............G...-.......................----   118
TfSat09_F1cl156x122L_fLG3_26965766    ---.............................................................................-..............G...-.......................----   118
TfSat09_F1cl156x122L_fLG3_26966010    ---.............................................................................-..............G...-.......................----   118
TfSat09_F1cl156x122L_fLG3_26966621    ---.............................................................................-..............G...-.......................----   118
TfSat09_F1cl156x122L_fLG3_26967230    ---.............................................................................-..............G...-.......................----   118
TfSat09_F1cl156x122L_fLG3_26967474    ---.............................................................................-..............G...-.......................----   118
TfSat09_F1cl156x122L_fLG3_26967595    ---.............................................................................-..............G...-.......................----   118
TfSat09_F1cl156x122L_fLG3_26973376    ---.............................................................................-..............G...-.......................----   118
TfSat09_F1cl156x122L_fLG3_26973620    ---.............................................................................-..............G...-.......................----   118
TfSat09_F1cl156x122L_fLG3_26973864    ---.............................................................................-..............G...-.......................----   118
TfSat09_F1cl156x122L_fLG3_26973985    ---.............................................................................-..............G...-.......................----   118
TfSat09_F1cl156x122L_fLG3_26980224    ---.............................................................................-..............G...-.......................----   118
TfSat09_F1cl156x122L_fLG3_26980712    ---.............................................................................-..............G...-.......................----   118
TfSat09_F1cl156x122L_fLG3_26980956    ---.............................................................................-..............G...-.......................----   118
TfSat09_F1cl156x122L_fLG3_26981077    ---.............................................................................-..............G...-.......................----   118
TfSat09_F1cl156x122L_fLG3_26981564    ---.............................................................................-..............G...-.......................----   118
TfSat09_F1cl156x122L_fLG3_26981929    ---.............................................................................-..............G...-.......................----   118
TfSat09_F1cl156x122L_fLG3_26982418    ---.............................................................................-..............G...-.......................----   118
TfSat09_F1cl156x122L_fLG3_26983027    ---.............................................................................-..............G...-.......................----   118
TfSat09_F1cl156x122L_fLG3_26983271    ---.............................................................................-..............G...-.......................----   118
TfSat09_F1cl156x122L_fLG3_26983638    ---.............................................................................-..............G...-.......................----   118
TfSat09_F1cl156x122L_fLG3_26989172    ---.............................................................................-..............G...-.......................----   118
TfSat09_F1cl156x122L_fLG3_26989293    ---.............................................................................-..............G...-.......................----   118
TfSat09_F1cl156x122L_fLG3_26989659    ---.............................................................................-..............G...-.......................----   118
TfSat09_F1cl156x122L_fLG3_26990391    ---.............................................................................-..............G...-.......................----   118
TfSat09_F1cl156x122L_fLG3_26990633    ---.............................................................................-..............G...-.......................----   118
TfSat09_F1cl156x122L_fLG3_26990999    ---.............................................................................-..............G...-.......................----   118
TfSat09_F1cl156x122L_fLG3_26991243    ---.............................................................................-..............G...-.......................----   118
TfSat09_F1cl156x122L_fLG3_26992095    ---.............................................................................-..............G...-.......................----   118
TfSat09_F1cl156x122L_fLG3_26992216    ---.............................................................................-..............G...-.......................----   118
TfSat09_F1cl156x122L_fLG3_26992655    ---.............................................................................-..............G...-.......................----   118
TfSat09_F1cl156x122L_fLG3_26993021    ---.............................................................................-..............G...-.......................----   118
TfSat09_F1cl156x122L_fLG3_26993265    ---.............................................................................-..............G...-.......................----   118
TfSat09_F1cl156x122L_fLG3_26993753    ---.............................................................................-..............G...-.......................----   118
TfSat09_F1cl156x122L_fLG3_26994192    ---.............................................................................-..............G...-.......................----   118
TfSat09_F1cl156x122L_fLG3_26994757    ---.............................................................................-..............G...-.......................----   118
TfSat09_F1cl156x122L_fLG3_26996465    ---.............................................................................-..............G...-.......................----   118
TfSat09_F1cl156x122L_fLG3_27001806    ---.............................................................................-..............G...-.......................----   118
TfSat09_F1cl156x122L_fLG3_27002048    ---.............................................................................-..............G...-.......................----   118
TfSat09_F1cl156x122L_fLG3_27003632    ---.............................................................................-..............G...-.......................----   118
TfSat09_F1cl156x122L_fLG3_27003753    ---.............................................................................-..............G...-.......................----   118
TfSat09_F1cl156x122L_fLG3_27004423    ---.............................................................................-..............G...-.......................----   118
TfSat09_F1cl156x122L_fLG3_27004667    ---.............................................................................-..............G...-.......................----   118
TfSat09_F1cl156x122L_fLG3_27005160    ---.............................................................................-..............G...-.......................----   118
TfSat09_F1cl156x122L_fLG3_27005404    ---.............................................................................-..............G...-.......................----   118
TfSat09_F1cl156x122L_fLG3_27005894    ---.............................................................................-..............G...-.......................----   118
TfSat09_F1cl156x122L_fLG3_27006015    ---.............................................................................-..............G...-.......................----   118
```

```
TfSat09_F1cl156x122L_fLG3_27006136    ---.............................................................................-..............G...-.......................----  118
TfSat09_F1cl156x122L_fLG3_27006257    ---.............................................................................-..............G...-.......................----  118
TfSat09_F1cl156x122L_fLG3_27006502    ---.............................................................................-..............G...-.......................----  118
TfSat09_F1cl156x122L_fLG3_27006623    ---.............................................................................-..............G...-.......................----  118
TfSat09_F1cl156x122L_fLG3_27006865    ---.............................................................................-..............G...-.......................----  118
TfSat09_F1cl156x122L_fLG3_27007109    ---.............................................................................-..............G...-.......................----  118
TfSat09_F1cl156x122L_fLG3_27007597    ---.............................................................................-..............G...-.......................----  118
TfSat09_F1cl156x122L_fLG3_27007841    ---.............................................................................-..............G...-.......................----  118
TfSat09_F1cl156x122L_fLG3_27008084    ---.............................................................................-..............G...-.......................----  118
TfSat09_F1cl156x122L_fLG3_27008205    ---.............................................................................-..............G...-.......................----  118
TfSat09_F1cl156x122L_fLG3_27008326    ---.............................................................................-..............G...-.......................----  118
TfSat09_F1cl156x122L_fLG3_26979645    ---.............................................................................-..............G...-........................C..  122
TfSat09_F1cl156x122L_fLG3_26991486    ---...........................................G.................................-..............G...-........................---  119
TfSat09_F1cl156x122L_fLG3_26995367    ---...........................................G.................................-..............G...-........................---  119
TfSat09_F1cl156x122L_fLG3_26995856    ---...........................................G.................................-..............G...-........................---  119
TfSat09_F1cl156x122L_fLG3_27002657    ---...........................................G.................................-..............G...-........................---  119
TfSat09_F1cl156x122L_fLG3_26940864    ---...........................................G.................................-..............G...-.......................----  118
TfSat09_F1cl156x122L_fLG3_26962924    ---...........................................G.................................-..............G...-.......................----  118
TfSat09_F1cl156x122L_fLG3_26991609    ---...........................................G.................................-..............G...-.......................----  118
TfSat09_F1cl156x122L_fLG3_26995490    ---...........................................G.................................-..............G...-.......................----  118
TfSat09_F1cl156x122L_fLG3_26995979    ---...........................................G.................................-..............G...-.......................----  118
TfSat09_F1cl156x122L_fLG3_26996222    ---...........................................G.................................-..............G...-.......................----  118
TfSat09_F1cl156x122L_fLG3_27002292    ---...........................................G.................................-..............G...-.......................----  118
TfSat09_F1cl156x122L_fLG3_27002780    ---...........................................G.................................-..............G...-.......................----  118
TfSat09_F1cl156x122L_fLG3_27003146    ---...........................................G.................................-..............G...-.......................----  118
TfSat09_F1cl156x122L_fLG3_27003389    ---...........................................G.................................-..............G...-.......................----  118
TfSat09_F1cl156x122L_fLG3_27006744    ---.......................................................A.....................-..............G...-.......................----  118
TfSat09_F1cl156x122L_fLG3_26991973    ---.............................................................................-..............G...-........A..................  122
TfSat09_F1cl156x122L_fLG3_27003510    ---.............................................................................-..............G...-........A..................  122
TfSat09_F1cl156x122L_fLG3_27008568    ---........T....................................................................-..............G...-...........................  122
TfSat09_F1cl156x122L_fLG3_26991852    ---...........................................GC................................-..............G...-.......................----  118
TfSat09_F1cl156x122L_fLG3_27008690    ---........T....................................................................-..............G...-.......A...............----  118
TfSat09_F1cl156x122L_fLG3_27016450    ---.......T.....................................................................-..............G...-........................---  119
TfSat09_F1cl156x122L_fLG3_26996586    ---.....................................................G.......................-..............G...-...........................  122
TfSat09_F1cl156x122L_fLG3_27008811    ---.....................G.......................................................-..............G...-.........................--  120
TfSat09_F1cl156x122L_fLG3_26982296    ---.........................................................................A...-..............G...-...........................  122
TfSat09_F1cl156x122L_fLG3_26993632    ---.............................................................................-.............CG...-.......................----  118
TfSat09_F1cl156x122L_fLG3_26989537    ---..........................................................C..................-..............G...-...........................  122
TfSat09_F1cl156x122L_fLG3_26951268    ---..........................................................C..................-..............G...-........................---  119
TfSat09_F1cl156x122L_fLG3_26965887    ---..........................................................C..................-..............G...-........................---  119
TfSat09_F1cl156x122L_fLG3_26966131    ---..........................................................C..................-..............G...-........................---  119
TfSat09_F1cl156x122L_fLG3_26967351    ---..........................................................C..................-..............G...-........................---  119
TfSat09_F1cl156x122L_fLG3_26973497    ---..........................................................C..................-..............G...-........................---  119
TfSat09_F1cl156x122L_fLG3_26973741    ---..........................................................C..................-..............G...-........................---  119
TfSat09_F1cl156x122L_fLG3_26980101    ---..........................................................C..................-..............G...-........................---  119
TfSat09_F1cl156x122L_fLG3_26982173    ---..........................................................C..................-..............G...-........................---  119
TfSat09_F1cl156x122L_fLG3_26983148    ---..........................................................C..................-..............G...-........................---  119
TfSat09_F1cl156x122L_fLG3_26983392    ---..........................................................C..................-..............G...-........................---  119
TfSat09_F1cl156x122L_fLG3_26990146    ---..........................................................C..................-..............G...-........................---  119
TfSat09_F1cl156x122L_fLG3_26990754    ---..........................................................C..................-..............G...-........................---  119
TfSat09_F1cl156x122L_fLG3_26993142    ---..........................................................C..................-..............G...-........................---  119
TfSat09_F1cl156x122L_fLG3_26993386    ---..........................................................C..................-..............G...-........................---  119
TfSat09_F1cl156x122L_fLG3_27004544    ---..........................................................C..................-..............G...-........................---  119
TfSat09_F1cl156x122L_fLG3_27007230    ---..........................................................C..................-..............G...-........................---  119
TfSat09_F1cl156x122L_fLG3_26949937    ---..........................................................C..................-..............G...-.......................----  118
TfSat09_F1cl156x122L_fLG3_26950058    ---..........................................................C..................-..............G...-.......................----  118
TfSat09_F1cl156x122L_fLG3_26950179    ---..........................................................C..................-..............G...-.......................----  118
TfSat09_F1cl156x122L_fLG3_26950300    ---..........................................................C..................-..............G...-.......................----  118
TfSat09_F1cl156x122L_fLG3_26950421    ---..........................................................C..................-..............G...-.......................----  118
TfSat09_F1cl156x122L_fLG3_26950542    ---..........................................................C..................-..............G...-.......................----  118
TfSat09_F1cl156x122L_fLG3_26950663    ---..........................................................C..................-..............G...-.......................----  118
TfSat09_F1cl156x122L_fLG3_26950784    ---..........................................................C..................-..............G...-.......................----  118
TfSat09_F1cl156x122L_fLG3_26950905    ---..........................................................C..................-..............G...-.......................----  118
TfSat09_F1cl156x122L_fLG3_26951026    ---..........................................................C..................-..............G...-.......................----  118
TfSat09_F1cl156x122L_fLG3_26951147    ---..........................................................C..................-..............G...-.......................----  118
TfSat09_F1cl156x122L_fLG3_26980345    ---..........................................................C..................-..............G...-.......................----  118
TfSat09_F1cl156x122L_fLG3_26981321    ---..........................................................C..................-..............G...-.......................----  118
TfSat09_F1cl156x122L_fLG3_26981685    ---..........................................................C..................-..............G...-.......................----  118
TfSat09_F1cl156x122L_fLG3_26990025    ---..........................................................C..................-..............G...-.......................----  118
TfSat09_F1cl156x122L_fLG3_26990512    ---..........................................................C..................-..............G...-.......................----  118
TfSat09_F1cl156x122L_fLG3_27001927    ---..........................................................C..................-..............G...-.......................----  118
```

```
TfSat09_F1cl156x122L_fLG3_27005525    ---..........................................................C..................-..............G...-.......................----  118
TfSat09_F1cl156x122L_fLG3_26988927    ---.......C..................................................C..................-..............G...-........................---  119
TfSat09_F1cl156x122L_fLG3_26991120    ---..........................................................C..................-..............G...-.........A..............---  119
TfSat09_F1cl156x122L_fLG3_27003023    ---..............C............................G.................................-..............G...-........................---  119
TfSat09_F1cl156x122L_fLG3_26903726    ---.............................................................................-...T..A.......G...-...........................  122
TfSat09_F1cl156x122L_fLG3_26904093    ---.............................................................................-...T..A.......G...-...........................  122
TfSat09_F1cl156x122L_fLG3_26904215    ---.............................................................................-...T..A.......G...-...........................  122
TfSat09_F1cl156x122L_fLG3_27007962    ---.............................................................................-...T..A.......G...-...........................  122
TfSat09_F1cl156x122L_fLG3_27010932    ---.............................................................................-...T..A.......G...-...........................  122
TfSat09_F1cl156x122L_fLG3_27016328    ---.............................................................................-...T..A.......G...-...........................  122
TfSat09_F1cl156x122L_fLG3_26903848    ---.............................................................................-...T..A.......G...-........................---  119
TfSat09_F1cl156x122L_fLG3_26904337    ---.............................................................................-...T..A.......G...-........................---  119
TfSat09_F1cl156x122L_fLG3_27007718    ---.............................................................................-...T..A.......G...-........................---  119
TfSat09_F1cl156x122L_fLG3_27008447    ---......A......................................................................-...T..A.......G...-.......................----  118
TfSat09_F1cl156x122L_fLG3_27007475    ---.......C.....................................................................-...T..A.......G...-...........................  122
TfSat09_F1cl156x122L_fLG3_26995123    ---...........................................G.................................-..............G...-..............AA.......G.A-  121
TfSat09_F1cl156x122L_fLG3_26996708    ---.....................................................G.......................-..............G...T...........................  123
TfSat09_var2_fLG2_8314852 (reversed)  -.......-------------------------------------------------C.....TC.T.............-....G..A...G.A....-A....G.C...C............---   72
TfSat09_var2_fLG2_8321610 (reversed)  -.......-------------------------------------------------C.....TC.T.............-....G..A...G.A....-A....G.C...C............---   72
TfSat09_var2_fLG2_8321682 (reversed)  -.......-------------------------------------------------C.....TC.T.............-....G..A...G.A....-A....G.C...C............---   72
TfSat09_var2_fLG2_8321754 (reversed)  -.......-------------------------------------------------C.....TC.T.............-....G..A...G.A....-A....G.C...C............---   72
TfSat09_var2_fLG2_8321826 (reversed)  -.......-------------------------------------------------C.....TC.T.............-....G..A...G.A....-A....G.C...C............---   72
TfSat09_var2_fLG2_8321898 (reversed)  -.......-------------------------------------------------C.....TC.T.............-....G..A...G.A....-A....G.C...C............---   72
TfSat09_var2_fLG2_8322186 (reversed)  -.......-------------------------------------------------C.....TC.T.............-....G..A...G.A....-A....G.C...C............---   72
TfSat09_var2_fLG2_8322402 (reversed)  -.......-------------------------------------------------C.....TC.T.............-....G..A...G.A....-A....G.C...C............---   72
TfSat09_var2_fLG2_8322546 (reversed)  -.......-------------------------------------------------C.....TC.T.............-....G..A...G.A....-A....G.C...C............---   72
TfSat09_var2_fLG2_8322690 (reversed)  -.......-------------------------------------------------C.....TC.T.............-....G..A...G.A....-A....G.C...C............---   72
TfSat09_var2_fLG2_8322762 (reversed)  -.......-------------------------------------------------C.....TC.T.............-....G..A...G.A....-A....G.C...C............---   72
TfSat09_var2_fLG2_8322906 (reversed)  -.......-------------------------------------------------C.....TC.T.............-....G..A...G.A....-A....G.C...C............---   72
TfSat09_var2_fLG2_8328259 (reversed)  -.......-------------------------------------------------C.....TC.T.............-....G..A...G.A....-A....G.C...C............---   72
TfSat09_var2_fLG2_8328331 (reversed)  -.......-------------------------------------------------C.....TC.T.............-....G..A...G.A....-A....G.C...C............---   72
TfSat09_var2_fLG2_8328403 (reversed)  -.......-------------------------------------------------C.....TC.T.............-....G..A...G.A....-A....G.C...C............---   72
TfSat09_var2_fLG2_8328475 (reversed)  -.......-------------------------------------------------C.....TC.T.............-....G..A...G.A....-A....G.C...C............---   72
TfSat09_var2_fLG2_8328547 (reversed)  -.......-------------------------------------------------C.....TC.T.............-....G..A...G.A....-A....G.C...C............---   72
TfSat09_var2_fLG2_8328619 (reversed)  -.......-------------------------------------------------C.....TC.T.............-....G..A...G.A....-A....G.C...C............---   72
TfSat09_var2_fLG2_8328691 (reversed)  -.......-------------------------------------------------C.....TC.T.............-....G..A...G.A....-A....G.C...C............---   72
TfSat09_var2_fLG2_8328835 (reversed)  -.......-------------------------------------------------C.....TC.T.............-....G..A...G.A....-A....G.C...C............---   72
TfSat09_var2_fLG2_8329195 (reversed)  -.......-------------------------------------------------C.....TC.T.............-....G..A...G.A....-A....G.C...C............---   72
TfSat09_var2_fLG2_8329267 (reversed)  -.......-------------------------------------------------C.....TC.T.............-....G..A...G.A....-A....G.C...C............---   72
TfSat09_var2_fLG2_8329411 (reversed)  -.......-------------------------------------------------C.....TC.T.............-....G..A...G.A....-A....G.C...C............---   72
TfSat09_var2_fLG2_8329483 (reversed)  -.......-------------------------------------------------C.....TC.T.............-....G..A...G.A....-A....G.C...C............---   72
TfSat09_var2_fLG2_8329555 (reversed)  -.......-------------------------------------------------C.....TC.T.............-....G..A...G.A....-A....G.C...C............---   72
TfSat09_var2_fLG2_8336358 (reversed)  -.......-------------------------------------------------C.....TC.T.............-....G..A...G.A....-A....G.C...C............---   72
TfSat09_var2_fLG2_8341280 (reversed)  -.......-------------------------------------------------C.....TC.T.............-....G..A...G.A....-A....G.C...C............---   72
TfSat09_var2_fLG2_8341423 (reversed)  -.......-------------------------------------------------C.....TC.T.............-....G..A...G.A....-A....G.C...C............---   72
TfSat09_var2_fLG2_8341495 (reversed)  -.......-------------------------------------------------C.....TC.T.............-....G..A...G.A....-A....G.C...C............---   72
TfSat09_var2_fLG2_8341709 (reversed)  -.......-------------------------------------------------C.....TC.T.............-....G..A...G.A....-A....G.C...C............---   72
TfSat09_var2_fLG2_8341853 (reversed)  -.......-------------------------------------------------C.....TC.T.............-....G..A...G.A....-A....G.C...C............---   72
TfSat09_var2_fLG2_8341925 (reversed)  -.......-------------------------------------------------C.....TC.T.............-....G..A...G.A....-A....G.C...C............---   72
TfSat09_var2_fLG2_8342140 (reversed)  -.......-------------------------------------------------C.....TC.T.............-....G..A...G.A....-A....G.C...C............---   72
TfSat09_var2_fLG2_8342212 (reversed)  -.......-------------------------------------------------C.....TC.T.............-....G..A...G.A....-A....G.C...C............---   72
TfSat09_var2_fLG2_8342427 (reversed)  -.......-------------------------------------------------C.....TC.T.............-....G..A...G.A....-A....G.C...C............---   72
TfSat09_var2_fLG2_8342643 (reversed)  -.......-------------------------------------------------C.....TC.T.............-....G..A...G.A....-A....G.C...C............---   72
TfSat09_var2_fLG2_8342860 (reversed)  -.......-------------------------------------------------C.....TC.T.............-....G..A...G.A....-A....G.C...C............---   72
TfSat09_var2_fLG2_8343279 (reversed)  -.......-------------------------------------------------C.....TC.T.............-....G..A...G.A....-A....G.C...C............---   72
TfSat09_var2_fLG2_8343496 (reversed)  -.......-------------------------------------------------C.....TC.T.............-....G..A...G.A....-A....G.C...C............---   72
TfSat09_var2_fLG2_8343645 (reversed)  -.......-------------------------------------------------C.....TC.T.............-....G..A...G.A....-A....G.C...C............---   72
TfSat09_var2_fLG2_8344436 (reversed)  -.......-------------------------------------------------C.....TC.T.............-....G..A...G.A....-A....G.C...C............---   72
TfSat09_var2_fLG2_8344580 (reversed)  -.......-------------------------------------------------C.....TC.T.............-....G..A...G.A....-A....G.C...C............---   72
TfSat09_var2_fLG2_8344724 (reversed)  -.......-------------------------------------------------C.....TC.T.............-....G..A...G.A....-A....G.C...C............---   72
TfSat09_var2_fLG2_8344796 (reversed)  -.......-------------------------------------------------C.....TC.T.............-....G..A...G.A....-A....G.C...C............---   72
TfSat09_var2_fLG2_8345215 (reversed)  -.......-------------------------------------------------C.....TC.T.............-....G..A...G.A....-A....G.C...C............---   72
TfSat09_var2_fLG2_8345432 (reversed)  -.......-------------------------------------------------C.....TC.T.............-....G..A...G.A....-A....G.C...C............---   72
TfSat09_var2_fLG2_8350903 (reversed)  -.......-------------------------------------------------C.....TC.T.............-....G..A...G.A....-A....G.C...C............---   72
TfSat09_var2_fLG2_8351047 (reversed)  -.......-------------------------------------------------C.....TC.T.............-....G..A...G.A....-A....G.C...C............---   72
TfSat09_var2_fLG2_8351191 (reversed)  -.......-------------------------------------------------C.....TC.T.............-....G..A...G.A....-A....G.C...C............---   72
TfSat09_var2_fLG2_8351335 (reversed)  -.......-------------------------------------------------C.....TC.T.............-....G..A...G.A....-A....G.C...C............---   72
TfSat09_var2_fLG2_8351550 (reversed)  -.......-------------------------------------------------C.....TC.T.............-....G..A...G.A....-A....G.C...C............---   72
TfSat09_var2_fLG2_8351622 (reversed)  -.......-------------------------------------------------C.....TC.T.............-....G..A...G.A....-A....G.C...C............---   72
TfSat09_var2_fLG2_17027370 (reversed) -.......-------------------------------------------------C.....TC.T.............-....G..A...G.A....-A....G.C...C............---   72
TfSat09_var2_fLG2_17033871 (reversed) -.......-------------------------------------------------C.....TC.T.............-....G..A...G.A....-A....G.C...C............---   72
```

```
TfSat09_var2_fLG2_17033943 (reversed) -.......------------------------------------------------C.....TC.T.............-....G..A...G.A....-A....G.C...C............---   72
TfSat09_var2_fLG2_17034015 (reversed) -.......------------------------------------------------C.....TC.T.............-....G..A...G.A....-A....G.C...C............---   72
TfSat09_var2_fLG2_17034087 (reversed) -.......------------------------------------------------C.....TC.T.............-....G..A...G.A....-A....G.C...C............---   72
TfSat09_var2_fLG2_17034159 (reversed) -.......------------------------------------------------C.....TC.T.............-....G..A...G.A....-A....G.C...C............---   72
TfSat09_var2_fLG2_17034447 (reversed) -.......------------------------------------------------C.....TC.T.............-....G..A...G.A....-A....G.C...C............---   72
TfSat09_var2_fLG2_17034519 (reversed) -.......------------------------------------------------C.....TC.T.............-....G..A...G.A....-A....G.C...C............---   72
TfSat09_var2_fLG2_17034591 (reversed) -.......------------------------------------------------C.....TC.T.............-....G..A...G.A....-A....G.C...C............---   72
TfSat09_var2_fLG2_17038483 (reversed) -.......------------------------------------------------C.....TC.T.............-....G..A...G.A....-A....G.C...C............---   72
TfSat09_var2_fLG2_17043491 (reversed) -.......------------------------------------------------C.....TC.T.............-....G..A...G.A....-A....G.C...C............---   72
TfSat09_var2_fLG2_17043563 (reversed) -.......------------------------------------------------C.....TC.T.............-....G..A...G.A....-A....G.C...C............---   72
TfSat09_var2_fLG2_17043635 (reversed) -.......------------------------------------------------C.....TC.T.............-....G..A...G.A....-A....G.C...C............---   72
TfSat09_var2_fLG2_17044241 (reversed) -.......------------------------------------------------C.....TC.T.............-....G..A...G.A....-A....G.C...C............---   72
TfSat09_var2_fLG2_17044385 (reversed) -.......------------------------------------------------C.....TC.T.............-....G..A...G.A....-A....G.C...C............---   72
TfSat09_var2_fLG2_17044529 (reversed) -.......------------------------------------------------C.....TC.T.............-....G..A...G.A....-A....G.C...C............---   72
TfSat09_var2_fLG2_17044601 (reversed) -.......------------------------------------------------C.....TC.T.............-....G..A...G.A....-A....G.C...C............---   72
TfSat09_var2_fLG2_17044745 (reversed) -.......------------------------------------------------C.....TC.T.............-....G..A...G.A....-A....G.C...C............---   72
TfSat09_var2_fLG2_17044817 (reversed) -.......------------------------------------------------C.....TC.T.............-....G..A...G.A....-A....G.C...C............---   72
TfSat09_var2_fLG2_17044889 (reversed) -.......------------------------------------------------C.....TC.T.............-....G..A...G.A....-A....G.C...C............---   72
TfSat09_var2_fLG2_17044961 (reversed) -.......------------------------------------------------C.....TC.T.............-....G..A...G.A....-A....G.C...C............---   72
TfSat09_var2_fLG2_17045176 (reversed) -.......------------------------------------------------C.....TC.T.............-....G..A...G.A....-A....G.C...C............---   72
TfSat09_var2_fLG2_17045248 (reversed) -.......------------------------------------------------C.....TC.T.............-....G..A...G.A....-A....G.C...C............---   72
TfSat09_var2_fLG2_17045320 (reversed) -.......------------------------------------------------C.....TC.T.............-....G..A...G.A....-A....G.C...C............---   72
TfSat09_var2_fLG2_17045463 (reversed) -.......------------------------------------------------C.....TC.T.............-....G..A...G.A....-A....G.C...C............---   72
TfSat09_var2_fLG2_17046106 (reversed) -.......------------------------------------------------C.....TC.T.............-....G..A...G.A....-A....G.C...C............---   72
TfSat09_var2_fLG2_17046748 (reversed) -.......------------------------------------------------C.....TC.T.............-....G..A...G.A....-A....G.C...C............---   72
TfSat09_var2_fLG2_17047320 (reversed) -.......------------------------------------------------C.....TC.T.............-....G..A...G.A....-A....G.C...C............---   72
TfSat09_var2_fLG2_17047536 (reversed) -.......------------------------------------------------C.....TC.T.............-....G..A...G.A....-A....G.C...C............---   72
TfSat09_var2_fLG2_17047752 (reversed) -.......------------------------------------------------C.....TC.T.............-....G..A...G.A....-A....G.C...C............---   72
TfSat09_var2_fLG2_17048325 (reversed) -.......------------------------------------------------C.....TC.T.............-....G..A...G.A....-A....G.C...C............---   72
TfSat09_var2_fLG2_17048540 (reversed) -.......------------------------------------------------C.....TC.T.............-....G..A...G.A....-A....G.C...C............---   72
TfSat09_var2_fLG2_17049185 (reversed) -.......------------------------------------------------C.....TC.T.............-....G..A...G.A....-A....G.C...C............---   72
TfSat09_var2_fLG2_17049329 (reversed) -.......------------------------------------------------C.....TC.T.............-....G..A...G.A....-A....G.C...C............---   72
TfSat09_var2_fLG2_17049473 (reversed) -.......------------------------------------------------C.....TC.T.............-....G..A...G.A....-A....G.C...C............---   72
TfSat09_var2_fLG2_17049545 (reversed) -.......------------------------------------------------C.....TC.T.............-....G..A...G.A....-A....G.C...C............---   72
TfSat09_var2_fLG2_17049617 (reversed) -.......------------------------------------------------C.....TC.T.............-....G..A...G.A....-A....G.C...C............---   72
TfSat09_var2_fLG2_17049689 (reversed) -.......------------------------------------------------C.....TC.T.............-....G..A...G.A....-A....G.C...C............---   72
TfSat09_var2_fLG2_17049761 (reversed) -.......------------------------------------------------C.....TC.T.............-....G..A...G.A....-A....G.C...C............---   72
TfSat09_var2_fLG2_17049833 (reversed) -.......------------------------------------------------C.....TC.T.............-....G..A...G.A....-A....G.C...C............---   72
TfSat09_var2_fLG2_17049977 (reversed) -.......------------------------------------------------C.....TC.T.............-....G..A...G.A....-A....G.C...C............---   72
TfSat09_var2_fLG2_17050049 (reversed) -.......------------------------------------------------C.....TC.T.............-....G..A...G.A....-A....G.C...C............---   72
TfSat09_var2_fLG2_17050193 (reversed) -.......------------------------------------------------C.....TC.T.............-....G..A...G.A....-A....G.C...C............---   72
TfSat09_var2_fLG2_17050265 (reversed) -.......------------------------------------------------C.....TC.T.............-....G..A...G.A....-A....G.C...C............---   72
TfSat09_var2_fLG2_17055572 (reversed) -.......------------------------------------------------C.....TC.T.............-....G..A...G.A....-A....G.C...C............---   72
TfSat09_var2_fLG2_17056637 (reversed) -.......------------------------------------------------C.....TC.T.............-....G..A...G.A....-A....G.C...C............---   72
TfSat09_var2_fLG2_17056709 (reversed) -.......------------------------------------------------C.....TC.T.............-....G..A...G.A....-A....G.C...C............---   72
TfSat09_var2_fLG2_17056854 (reversed) -.......------------------------------------------------C.....TC.T.............-....G..A...G.A....-A....G.C...C............---   72
TfSat09_var2_fLG2_17056999 (reversed) -.......------------------------------------------------C.....TC.T.............-....G..A...G.A....-A....G.C...C............---   72
TfSat09_var2_fLG2_17057144 (reversed) -.......------------------------------------------------C.....TC.T.............-....G..A...G.A....-A....G.C...C............---   72
TfSat09_var2_fLG2_17057505 (reversed) -.......------------------------------------------------C.....TC.T.............-....G..A...G.A....-A....G.C...C............---   72
TfSat09_var2_fLG2_8314924 (reversed)  --......------------------------------------------------C.....TC.T.............-....G..A...G.A....-A....G.C...C............---   71
TfSat09_var2_fLG2_8314997 (reversed)  --......------------------------------------------------C.....TC.T.............-....G..A...G.A....-A....G.C...C............---   71
TfSat09_var2_fLG2_8315070 (reversed)  --......------------------------------------------------C.....TC.T.............-....G..A...G.A....-A....G.C...C............---   71
TfSat09_var2_fLG2_8315215 (reversed)  --......------------------------------------------------C.....TC.T.............-....G..A...G.A....-A....G.C...C............---   71
TfSat09_var2_fLG2_8315288 (reversed)  --......------------------------------------------------C.....TC.T.............-....G..A...G.A....-A....G.C...C............---   71
TfSat09_var2_fLG2_8315361 (reversed)  --......------------------------------------------------C.....TC.T.............-....G..A...G.A....-A....G.C...C............---   71
TfSat09_var2_fLG2_8315434 (reversed)  --......------------------------------------------------C.....TC.T.............-....G..A...G.A....-A....G.C...C............---   71
TfSat09_var2_fLG2_8315507 (reversed)  --......------------------------------------------------C.....TC.T.............-....G..A...G.A....-A....G.C...C............---   71
TfSat09_var2_fLG2_8315652 (reversed)  --......------------------------------------------------C.....TC.T.............-....G..A...G.A....-A....G.C...C............---   71
TfSat09_var2_fLG2_8315725 (reversed)  --......------------------------------------------------C.....TC.T.............-....G..A...G.A....-A....G.C...C............---   71
TfSat09_var2_fLG2_8315870 (reversed)  --......------------------------------------------------C.....TC.T.............-....G..A...G.A....-A....G.C...C............---   71
TfSat09_var2_fLG2_8316015 (reversed)  --......------------------------------------------------C.....TC.T.............-....G..A...G.A....-A....G.C...C............---   71
TfSat09_var2_fLG2_8316560 (reversed)  --......------------------------------------------------C.....TC.T.............-....G..A...G.A....-A....G.C...C............---   71
TfSat09_var2_fLG2_17026714 (reversed) --......------------------------------------------------C.....TC.T.............-....G..A...G.A....-A....G.C...C............---   71
TfSat09_var2_fLG2_17026787 (reversed) --......------------------------------------------------C.....TC.T.............-....G..A...G.A....-A....G.C...C............---   71
TfSat09_var2_fLG2_17026860 (reversed) --......------------------------------------------------C.....TC.T.............-....G..A...G.A....-A....G.C...C............---   71
TfSat09_var2_fLG2_17026933 (reversed) --......------------------------------------------------C.....TC.T.............-....G..A...G.A....-A....G.C...C............---   71
TfSat09_var2_fLG2_17027006 (reversed) --......------------------------------------------------C.....TC.T.............-....G..A...G.A....-A....G.C...C............---   71
TfSat09_var2_fLG2_17027079 (reversed) --......------------------------------------------------C.....TC.T.............-....G..A...G.A....-A....G.C...C............---   71
TfSat09_var2_fLG2_17027224 (reversed) --......------------------------------------------------C.....TC.T.............-....G..A...G.A....-A....G.C...C............---   71
TfSat09_var2_fLG2_17027297 (reversed) --......------------------------------------------------C.....TC.T.............-....G..A...G.A....-A....G.C...C............---   71
TfSat09_var2_fLG2_17027514 (reversed) --......------------------------------------------------C.....TC.T.............-....G..A...G.A....-A....G.C...C............---   71
```

```
TfSat09_var2_fLG2_17027587 (reversed) --......------------------------------------------------C.....TC.T.............-....G..A...G.A....-A....G.C...C............---   71
TfSat09_var2_fLG2_17027660 (reversed) --......------------------------------------------------C.....TC.T.............-....G..A...G.A....-A....G.C...C............---   71
TfSat09_var2_fLG2_17027733 (reversed) --......------------------------------------------------C.....TC.T.............-....G..A...G.A....-A....G.C...C............---   71
TfSat09_var2_fLG2_17027806 (reversed) --......------------------------------------------------C.....TC.T.............-....G..A...G.A....-A....G.C...C............---   71
TfSat09_var2_fLG2_17027879 (reversed) --......------------------------------------------------C.....TC.T.............-....G..A...G.A....-A....G.C...C............---   71
TfSat09_var2_fLG2_17027952 (reversed) --......------------------------------------------------C.....TC.T.............-....G..A...G.A....-A....G.C...C............---   71
TfSat09_var2_fLG2_17028025 (reversed) --......------------------------------------------------C.....TC.T.............-....G..A...G.A....-A....G.C...C............---   71
TfSat09_var2_fLG2_17028098 (reversed) --......------------------------------------------------C.....TC.T.............-....G..A...G.A....-A....G.C...C............---   71
TfSat09_var2_fLG2_17028171 (reversed) --......------------------------------------------------C.....TC.T.............-....G..A...G.A....-A....G.C...C............---   71
TfSat09_var2_fLG2_17028316 (reversed) --......------------------------------------------------C.....TC.T.............-....G..A...G.A....-A....G.C...C............---   71
TfSat09_var2_fLG2_17028861 (reversed) --......------------------------------------------------C.....TC.T.............-....G..A...G.A....-A....G.C...C............---   71
TfSat09_var2_fLG2_17027442 (reversed) --......------------------------------------------------C.....TC.T.............-....G..A...G.A....-A....G.C...C...........----   70
TfSat09_var2_fLG2_17049905 (reversed) ---.....------------------------------------------------C.....TC.T.............-....G..A...G.A....-A....G.C...C............---   70
TfSat09_var2_fLG2_17057361 (reversed) -----...------------------------------------------------C.....TC.T.............-....G..A...G.A....-A....G.C...C............---   68
TfSat09_var2_fLG2_8317067 (reversed)  ----------------------------------------------------------------.T.............-....G..A...G.A....-A....G.C...C............---   57
TfSat09_var2_fLG2_8323050 (reversed)  ----------------------------------------------------------------.T.............-....G..A...G.A....-A....G.C...C............---   57
TfSat09_var2_fLG2_8329699 (reversed)  ----------------------------------------------------------------.T.............-....G..A...G.A....-A....G.C...C............---   57
TfSat09_var2_fLG2_8336501 (reversed)  ----------------------------------------------------------------.T.............-....G..A...G.A....-A....G.C...C............---   57
TfSat09_var2_fLG2_17029369 (reversed) ----------------------------------------------------------------.T.............-....G..A...G.A....-A....G.C...C............---   57
TfSat09_var2_fLG2_17034663 (reversed) ----------------------------------------------------------------.T.............-....G..A...G.A....-A....G.C...C............---   57
TfSat09_var2_fLG2_17038555 (reversed) ----------------------------------------------------------------.T.............-....G..A...G.A....-A....G.C...C............---   57
TfSat09_var2_fLG2_17043707 (reversed) ----------------------------------------------------------------.T.............-....G..A...G.A....-A....G.C...C............---   57
TfSat09_var2_fLG2_17050480 (reversed) ----------------------------------------------------------------.T.............-....G..A...G.A....-A....G.C...C............---   57
TfSat09_var2_fLG2_8321970 (reversed)  ........------------------------------------------------C.....T..T.............-....G..A...G.A....-A....G.C...C............---   73
TfSat09_var2_fLG2_17034231 (reversed) ........------------------------------------------------C.....T..T.............-....G..A...G.A....-A....G.C...C............---   73
TfSat09_var2_fLG2_8315143 (reversed)  -.......------------------------------------------------C.....T..T.............-....G..A...G.A....-A....G.C...C............---   72
TfSat09_var2_fLG2_8316412 (reversed)  -.......------------------------------------------------C.....T..T.............-....G..A...G.A....-A....G.C...C............---   72
TfSat09_var2_fLG2_8322114 (reversed)  -.......------------------------------------------------C.....T..T.............-....G..A...G.A....-A....G.C...C............---   72
TfSat09_var2_fLG2_8322258 (reversed)  -.......------------------------------------------------C.....T..T.............-....G..A...G.A....-A....G.C...C............---   72
TfSat09_var2_fLG2_8322330 (reversed)  -.......------------------------------------------------C.....T..T.............-....G..A...G.A....-A....G.C...C............---   72
TfSat09_var2_fLG2_8322474 (reversed)  -.......------------------------------------------------C.....T..T.............-....G..A...G.A....-A....G.C...C............---   72
TfSat09_var2_fLG2_8322618 (reversed)  -.......------------------------------------------------C.....T..T.............-....G..A...G.A....-A....G.C...C............---   72
TfSat09_var2_fLG2_8322834 (reversed)  -.......------------------------------------------------C.....T..T.............-....G..A...G.A....-A....G.C...C............---   72
TfSat09_var2_fLG2_8328763 (reversed)  -.......------------------------------------------------C.....T..T.............-....G..A...G.A....-A....G.C...C............---   72
TfSat09_var2_fLG2_8328907 (reversed)  -.......------------------------------------------------C.....T..T.............-....G..A...G.A....-A....G.C...C............---   72
TfSat09_var2_fLG2_8328979 (reversed)  -.......------------------------------------------------C.....T..T.............-....G..A...G.A....-A....G.C...C............---   72
TfSat09_var2_fLG2_8329123 (reversed)  -.......------------------------------------------------C.....T..T.............-....G..A...G.A....-A....G.C...C............---   72
TfSat09_var2_fLG2_8329339 (reversed)  -.......------------------------------------------------C.....T..T.............-....G..A...G.A....-A....G.C...C............---   72
TfSat09_var2_fLG2_8336286 (reversed)  -.......------------------------------------------------C.....T..T.............-....G..A...G.A....-A....G.C...C............---   72
TfSat09_var2_fLG2_8341781 (reversed)  -.......------------------------------------------------C.....T..T.............-....G..A...G.A....-A....G.C...C............---   72
TfSat09_var2_fLG2_8342355 (reversed)  -.......------------------------------------------------C.....T..T.............-....G..A...G.A....-A....G.C...C............---   72
TfSat09_var2_fLG2_17028713 (reversed) -.......------------------------------------------------C.....T..T.............-....G..A...G.A....-A....G.C...C............---   72
TfSat09_var2_fLG2_17034375 (reversed) -.......------------------------------------------------C.....T..T.............-....G..A...G.A....-A....G.C...C............---   72
TfSat09_var2_fLG2_17058410 (reversed) ---------------------------------------------------------.....T..T.............-....G..A...G.A....-A....G.C...C............---   64
TfSat09_var2_fLG2_8316922 (reversed)  -.......------------------------------------------------C.....TC.T.............-....G..A...G.A....-A....G.C...C...........----   71
TfSat09_var2_fLG2_8322043 (reversed)  -.......------------------------------------------------C.....TC.T.............-....G..A...G.A....-A....G.C...C...........----   71
TfSat09_var2_fLG2_8336142 (reversed)  -.......------------------------------------------------C.....TC.T.............-....G..A...G.A....-A....G.C...C...........----   71
TfSat09_var2_fLG2_8336214 (reversed)  -.......------------------------------------------------C.....TC.T.............-....G..A...G.A....-A....G.C...C...........----   71
TfSat09_var2_fLG2_8351407 (reversed)  -.......------------------------------------------------C.....TC.T.............-....G..A...G.A....-A....G.C...C...........----   71
TfSat09_var2_fLG2_17029224 (reversed) -.......------------------------------------------------C.....TC.T.............-....G..A...G.A....-A....G.C...C...........----   71
TfSat09_var2_fLG2_17034304 (reversed) -.......------------------------------------------------C.....TC.T.............-....G..A...G.A....-A....G.C...C...........----   71
TfSat09_var2_fLG2_17044674 (reversed) -.......------------------------------------------------C.....TC.T.............-....G..A...G.A....-A....G.C...C...........----   71
TfSat09_var2_fLG2_17045033 (reversed) -.......------------------------------------------------C.....TC.T.............-....G..A...G.A....-A....G.C...C...........----   71
TfSat09_var2_fLG2_17045392 (reversed) -.......------------------------------------------------C.....TC.T.............-....G..A...G.A....-A....G.C...C...........----   71
TfSat09_var2_fLG2_17045535 (reversed) -.......------------------------------------------------C.....TC.T.............-....G..A...G.A....-A....G.C...C...........----   71
TfSat09_var2_fLG2_17045606 (reversed) -.......------------------------------------------------C.....TC.T.............-....G..A...G.A....-A....G.C...C...........----   71
TfSat09_var2_fLG2_17045677 (reversed) -.......------------------------------------------------C.....TC.T.............-....G..A...G.A....-A....G.C...C...........----   71
TfSat09_var2_fLG2_17045748 (reversed) -.......------------------------------------------------C.....TC.T.............-....G..A...G.A....-A....G.C...C...........----   71
TfSat09_var2_fLG2_17045891 (reversed) -.......------------------------------------------------C.....TC.T.............-....G..A...G.A....-A....G.C...C...........----   71
TfSat09_var2_fLG2_17045962 (reversed) -.......------------------------------------------------C.....TC.T.............-....G..A...G.A....-A....G.C...C...........----   71
TfSat09_var2_fLG2_17046178 (reversed) -.......------------------------------------------------C.....TC.T.............-....G..A...G.A....-A....G.C...C...........----   71
TfSat09_var2_fLG2_17046321 (reversed) -.......------------------------------------------------C.....TC.T.............-....G..A...G.A....-A....G.C...C...........----   71
TfSat09_var2_fLG2_17046392 (reversed) -.......------------------------------------------------C.....TC.T.............-....G..A...G.A....-A....G.C...C...........----   71
TfSat09_var2_fLG2_17046463 (reversed) -.......------------------------------------------------C.....TC.T.............-....G..A...G.A....-A....G.C...C...........----   71
TfSat09_var2_fLG2_17046534 (reversed) -.......------------------------------------------------C.....TC.T.............-....G..A...G.A....-A....G.C...C...........----   71
TfSat09_var2_fLG2_17046677 (reversed) -.......------------------------------------------------C.....TC.T.............-....G..A...G.A....-A....G.C...C...........----   71
TfSat09_var2_fLG2_17046820 (reversed) -.......------------------------------------------------C.....TC.T.............-....G..A...G.A....-A....G.C...C...........----   71
TfSat09_var2_fLG2_17046891 (reversed) -.......------------------------------------------------C.....TC.T.............-....G..A...G.A....-A....G.C...C...........----   71
TfSat09_var2_fLG2_17046962 (reversed) -.......------------------------------------------------C.....TC.T.............-....G..A...G.A....-A....G.C...C...........----   71
TfSat09_var2_fLG2_17047105 (reversed) -.......------------------------------------------------C.....TC.T.............-....G..A...G.A....-A....G.C...C...........----   71
TfSat09_var2_fLG2_17047176 (reversed) -.......------------------------------------------------C.....TC.T.............-....G..A...G.A....-A....G.C...C...........----   71
```

```
TfSat09_var2_fLG2_17047392 (reversed) -.......-------------------------------------------------C.....TC.T.............-....G..A...G.A....-A....G.C...C...........----   71
TfSat09_var2_fLG2_17047608 (reversed) -.......-------------------------------------------------C.....TC.T.............-....G..A...G.A....-A....G.C...C...........----   71
TfSat09_var2_fLG2_17047824 (reversed) -.......-------------------------------------------------C.....TC.T.............-....G..A...G.A....-A....G.C...C...........----   71
TfSat09_var2_fLG2_17047895 (reversed) -.......-------------------------------------------------C.....TC.T.............-....G..A...G.A....-A....G.C...C...........----   71
TfSat09_var2_fLG2_17048038 (reversed) -.......-------------------------------------------------C.....TC.T.............-....G..A...G.A....-A....G.C...C...........----   71
TfSat09_var2_fLG2_17048181 (reversed) -.......-------------------------------------------------C.....TC.T.............-....G..A...G.A....-A....G.C...C...........----   71
TfSat09_var2_fLG2_17048397 (reversed) -.......-------------------------------------------------C.....TC.T.............-....G..A...G.A....-A....G.C...C...........----   71
TfSat09_var2_fLG2_17048468 (reversed) -.......-------------------------------------------------C.....TC.T.............-....G..A...G.A....-A....G.C...C...........----   71
TfSat09_var2_fLG2_17048612 (reversed) -.......-------------------------------------------------C.....TC.T.............-....G..A...G.A....-A....G.C...C...........----   71
TfSat09_var2_fLG2_17048683 (reversed) -.......-------------------------------------------------C.....TC.T.............-....G..A...G.A....-A....G.C...C...........----   71
TfSat09_var2_fLG2_17048754 (reversed) -.......-------------------------------------------------C.....TC.T.............-....G..A...G.A....-A....G.C...C...........----   71
TfSat09_var2_fLG2_17048970 (reversed) -.......-------------------------------------------------C.....TC.T.............-....G..A...G.A....-A....G.C...C...........----   71
TfSat09_var2_fLG2_17050337 (reversed) -.......-------------------------------------------------C.....TC.T.............-....G..A...G.A....-A....G.C...C...........----   71
TfSat09_var2_fLG2_17055790 (reversed) -.......-------------------------------------------------C.....TC.T.............-....G..A...G.A....-A....G.C...C...........----   71
TfSat09_var2_fLG2_8336431 (reversed)  -.......-------------------------------------------------C.....TC.T.............-....G..A...G.A....-A....G.C...C..........-----   70
TfSat09_var2_fLG2_8341353 (reversed)  -.......-------------------------------------------------C.....TC.T.............-....G..A...G.A....-A....G.C...C..........-----   70
TfSat09_var2_fLG2_8341568 (reversed)  -.......-------------------------------------------------C.....TC.T.............-....G..A...G.A....-A....G.C...C..........-----   70
TfSat09_var2_fLG2_8341639 (reversed)  -.......-------------------------------------------------C.....TC.T.............-....G..A...G.A....-A....G.C...C..........-----   70
TfSat09_var2_fLG2_8342070 (reversed)  -.......-------------------------------------------------C.....TC.T.............-....G..A...G.A....-A....G.C...C..........-----   70
TfSat09_var2_fLG2_8342285 (reversed)  -.......-------------------------------------------------C.....TC.T.............-....G..A...G.A....-A....G.C...C..........-----   70
TfSat09_var2_fLG2_8329054 (reversed)  -.......-------------------------------------------------C.....TC.T.............-....G..A...G.A....-A....G.C...C.........------   69
TfSat09_var2_fLG2_17058268 (reversed) -.......-------------------------------------------------C.....TC.T.............-....G..A...G.A....-A....G.C...C........-------   68
TfSat09_var2_fLG2_8321551 (reversed)  -.......-------------------------------------------------C.....TC.T.............-....G..A...G.A....-A....G.C...----------------   59
TfSat09_var2_fLG2_8328200 (reversed)  -.......-------------------------------------------------C.....TC.T.............-....G..A...G.A....-A....G.C...----------------   59
TfSat09_var2_fLG2_17033812 (reversed) -.......-------------------------------------------------C.....TC.T.............-....G..A...G.A....-A....G.C...----------------   59
TfSat09_var2_fLG2_8316214 (reversed)  -.......-------------------------------------------------C.....TC.T.............-....G..A...G.A....-A...-----------------------   52
TfSat09_var2_fLG2_17028515 (reversed) -.......-------------------------------------------------C.....TC.T.............-....G..A...G.A....-A...-----------------------   52
TfSat09_var2_fLG2_8342571 (reversed)  -.......-------------------------------------------------C.....TC.T.............-....G..A...G.A....-A....G.C...CT...........---   72
TfSat09_var2_fLG2_8342715 (reversed)  -.......-------------------------------------------------C.....TC.T.............-....G..A...G.A....-A....G.C...CT...........---   72
TfSat09_var2_fLG2_8342932 (reversed)  -.......-------------------------------------------------C.....TC.T.............-....G..A...G.A....-A....G.C...CT...........---   72
TfSat09_var2_fLG2_8343351 (reversed)  -.......-------------------------------------------------C.....TC.T.............-....G..A...G.A....-A....G.C...CT...........---   72
TfSat09_var2_fLG2_8344508 (reversed)  -.......-------------------------------------------------C.....TC.T.............-....G..A...G.A....-A....G.C...CT...........---   72
TfSat09_var2_fLG2_8344652 (reversed)  -.......-------------------------------------------------C.....TC.T.............-....G..A...G.A....-A....G.C...CT...........---   72
TfSat09_var2_fLG2_8345287 (reversed)  -.......-------------------------------------------------C.....TC.T.............-....G..A...G.A....-A....G.C...CT...........---   72
TfSat09_var2_fLG2_8345504 (reversed)  -.......-------------------------------------------------C.....TC.T.............-....G..A...G.A....-A....G.C...CT...........---   72
TfSat09_var2_fLG2_8350975 (reversed)  -.......-------------------------------------------------C.....TC.T.............-....G..A...G.A....-A....G.C...CT...........---   72
TfSat09_var2_fLG2_8351119 (reversed)  -.......-------------------------------------------------C.....TC.T.............-....G..A...G.A....-A....G.C...CT...........---   72
TfSat09_var2_fLG2_17044457 (reversed) -.......-------------------------------------------------C.....TC.T.............-....G..A...G.A....-A....G.C...CT...........---   72
TfSat09_var2_fLG2_17045819 (reversed) -.......-------------------------------------------------C.....TC.T.............-....G..A...G.A....-A....G.C...CT...........---   72
TfSat09_var2_fLG2_17046033 (reversed) -.......-------------------------------------------------C.....TC.T.............-....G..A...G.A....-A....G.C...CT...........---   72
TfSat09_var2_fLG2_17046249 (reversed) -.......-------------------------------------------------C.....TC.T.............-....G..A...G.A....-A....G.C...CT...........---   72
TfSat09_var2_fLG2_17046605 (reversed) -.......-------------------------------------------------C.....TC.T.............-....G..A...G.A....-A....G.C...CT...........---   72
TfSat09_var2_fLG2_17047033 (reversed) -.......-------------------------------------------------C.....TC.T.............-....G..A...G.A....-A....G.C...CT...........---   72
TfSat09_var2_fLG2_17047247 (reversed) -.......-------------------------------------------------C.....TC.T.............-....G..A...G.A....-A....G.C...CT...........---   72
TfSat09_var2_fLG2_17047463 (reversed) -.......-------------------------------------------------C.....TC.T.............-....G..A...G.A....-A....G.C...CT...........---   72
TfSat09_var2_fLG2_17047679 (reversed) -.......-------------------------------------------------C.....TC.T.............-....G..A...G.A....-A....G.C...CT...........---   72
TfSat09_var2_fLG2_17047966 (reversed) -.......-------------------------------------------------C.....TC.T.............-....G..A...G.A....-A....G.C...CT...........---   72
TfSat09_var2_fLG2_17048109 (reversed) -.......-------------------------------------------------C.....TC.T.............-....G..A...G.A....-A....G.C...CT...........---   72
TfSat09_var2_fLG2_17048252 (reversed) -.......-------------------------------------------------C.....TC.T.............-....G..A...G.A....-A....G.C...CT...........---   72
TfSat09_var2_fLG2_17048825 (reversed) -.......-------------------------------------------------C.....TC.T.............-....G..A...G.A....-A....G.C...CT...........---   72
TfSat09_var2_fLG2_17049041 (reversed) -.......-------------------------------------------------C.....TC.T.............-....G..A...G.A....-A....G.C...CT...........---   72
TfSat09_var2_fLG2_17049113 (reversed) -.......-------------------------------------------------C.....TC.T.............-....G..A...G.A....-A....G.C...CT...........---   72
TfSat09_var2_fLG2_17049401 (reversed) -.......-------------------------------------------------C.....TC.T.............-....G..A...G.A....-A....G.C...CT...........---   72
TfSat09_var2_fLG2_17055718 (reversed) -.......-------------------------------------------------C.....TC.T.............-....G..A...G.A....-A....G.C...CT...........---   72
TfSat09_var2_fLG2_17057216 (reversed) -.......-------------------------------------------------C.....TC.T.............-....G..A...G.A....-A....G.C...CT...........---   72
TfSat09_var2_fLG2_17057289 (reversed) -.......-------------------------------------------------C.....TC.T.............-....G..A...G.A....-A....G.C...CT...........---   72
TfSat09_var2_fLG2_17057577 (reversed) -.......-------------------------------------------------C.....TC.T.............-....G..A...G.A....-A....G.C...CT...........---   72
TfSat09_var2_fLG2_17057650 (reversed) -.......-------------------------------------------------C.....TC.T.............-....G..A...G.A....-A....G.C...CT...........---   72
TfSat09_var2_fLG2_17058337 (reversed) -.......-------------------------------------------------C.....TC.T.............-....G..A...G.A....-A....G.C...CT...........---   72
TfSat09_var2_fLG2_8344364 (reversed)  -.......-------------------------------------------------C.....TC.T.............-....G..A...G.A....-A......C...C............---   72
TfSat09_var2_fLG2_8350831 (reversed)  -.......-------------------------------------------------C.....TC.T.............-....G..A...G.A....-A......C...C............---   72
TfSat09_var2_fLG2_8336082 (reversed)  -.......-------------------------------------------------C.....TC.T.............-....G..A...G.A....-A......C...----------------   59
TfSat09_var2_fLG2_8341221 (reversed)  -.......-------------------------------------------------C.....TC.T.............-....G..A...G.A....-A......C...----------------   59
TfSat09_var2_fLG2_8343220 (reversed)  -.......-------------------------------------------------C.....TC.T.............-....G..A...G.A....-A......C...----------------   59
TfSat09_var2_fLG2_8344305 (reversed)  -.......-------------------------------------------------C.....TC.T.............-....G..A...G.A....-A......C...----------------   59
TfSat09_var2_fLG2_8345156 (reversed)  -.......-------------------------------------------------C.....TC.T.............-....G..A...G.A....-A......C...----------------   59
TfSat09_var2_fLG2_8350772 (reversed)  -.......-------------------------------------------------C.....TC.T.............-....G..A...G.A....-A......C...----------------   59
TfSat09_var2_fLG2_17038424 (reversed) -.......-------------------------------------------------C.....TC.T.............-....G..A...G.A....-A......C...----------------   59
TfSat09_var2_fLG2_17055513 (reversed) -.......-------------------------------------------------C.....TC.T.............-....G..A...G.A....-A......C...----------------   59
TfSat09_var2_fLG2_17056578 (reversed) -.......-------------------------------------------------C.....TC.T.............-....G..A...G.A....-A......C...----------------   59
TfSat09_var2_fLG2_17058205 (reversed) -.......-------------------------------------------------C.....TC.T.............-....G..A...G.A....-A......C...----------------   59
```

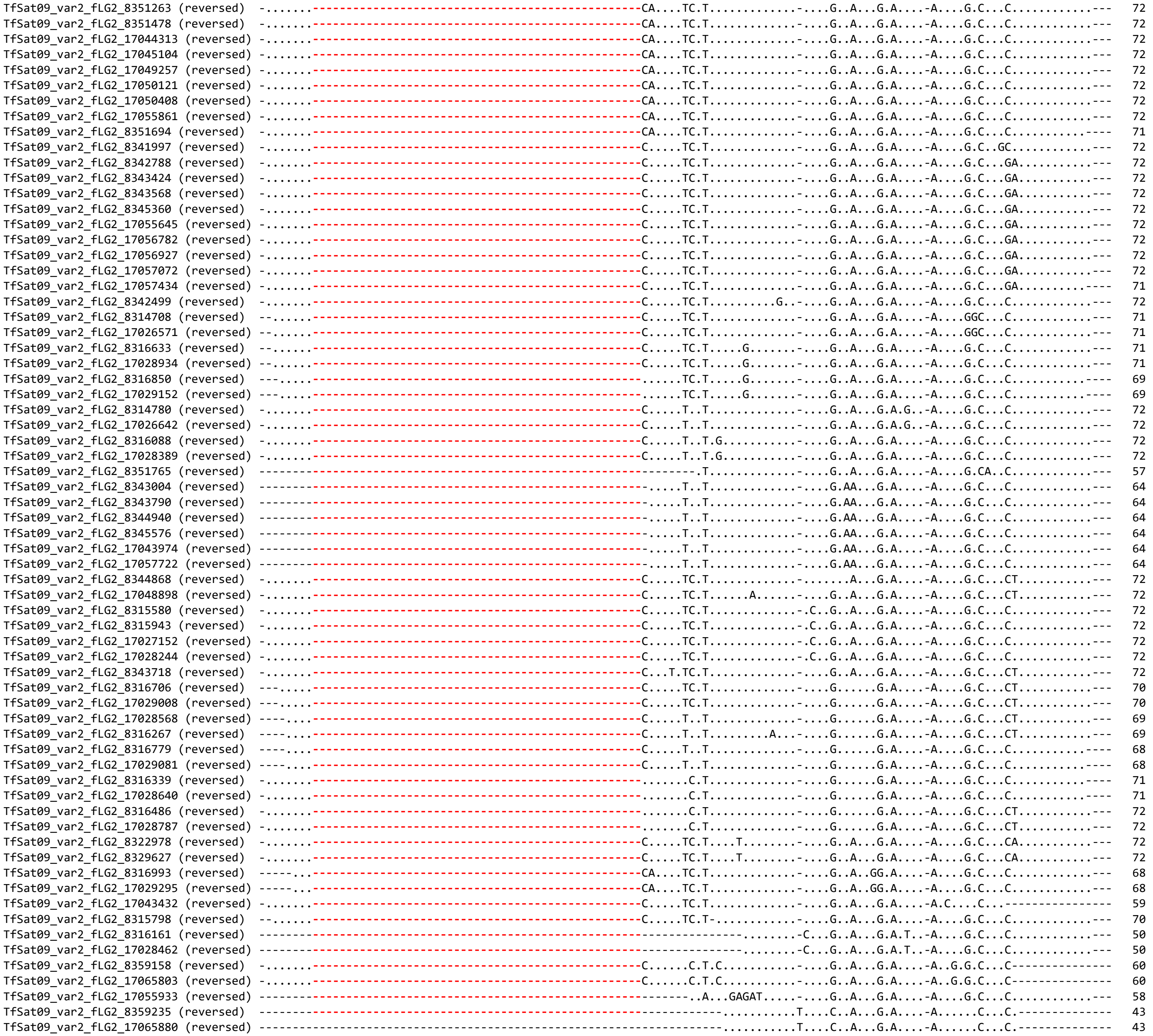

**Figure S4. A)** Alignment of NTS sequences in *T. castaneum*. **B)** Alignment of NTS (TfSat09) in *T. freemani*. Red dashed lines indicate the regions deleted in some NTS subgroups

**A**

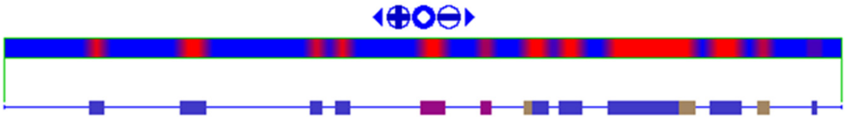

| Name | From | To | Name | From | To | Class | Dir | Sim | Pos/Mm:Ts | Score |
|---|---|---|---|---|---|---|---|---|---|---|
| jockeyONT | 483 | 562 | Jockey-1_DAlb | 1536 | 1619 | NonLTR/Jockey | d | 0.6988 | 1.3333 | 237 |
| jockeyONT | 1000 | 1140 | Jockey-15_DEl | 664 | 804 | NonLTR/Jockey | d | 0.6934 | 1.7273 | 342 |
| jockeyONT | 1735 | 1800 | Neptune-82_XL | 2005 | 2067 | NonLTR/Penelope/Neptune | c | 0.7879 | 1.6667 | 278 |
| jockeyONT | 1875 | 1957 | Jockey_Ele3 | 1713 | 1795 | NonLTR/Jockey | d | 0.7176 | 1.5833 | 254 |
| jockeyONT | 2360 | 2492 | MuDR-4_OES | 3619 | 3766 | DNA/MuDR | c | 0.7388 | 1.9286 | 218 |
| jockeyONT | 2695 | 2750 | Mariner-N5B_PM | 263 | 317 | DNA/Mariner | c | 0.7857 | 2.0000 | 210 |
| jockeyONT | 2941 | 2986 | Gypsy-27_DWil-I | 4947 | 4993 | LTR/Gypsy | d | 0.8298 | 1.5000 | 238 |
| jockeyONT | 2988 | 3073 | Jockey-5_DAlb | 5064 | 5147 | NonLTR/Jockey | d | 0.7765 | 2.0000 | 351 |
| jockeyONT | 3137 | 3261 | Jockey-13_DBp | 1974 | 2099 | NonLTR/Jockey | d | 0.7302 | 1.9375 | 413 |
| jockeyONT | 3412 | 3813 | DOC6_DM | 3141 | 3540 | NonLTR/Jockey | d | 0.6538 | 2.0357 | 343 |
| jockeyONT | 3814 | 3904 | Gypsy-43_DEu-I | 3645 | 3724 | LTR/Gypsy | d | 0.8000 | 2.2500 | 238 |
| jockeyONT | 3991 | 4161 | Jockey-14_AAe | 3810 | 3980 | NonLTR/Jockey | d | 0.7117 | 1.9091 | 340 |
| jockeyONT | 4260 | 4322 | COPIA-I_MT | 9167 | 9224 | LTR/Copia | c | 0.7869 | 1.6667 | 210 |
| jockeyONT | 4566 | 4592 | Tx1-5_LCh | 5392 | 5418 | NonLTR/Tx1 | d | 1.0000 | 99.0000 | 245 |

**B**

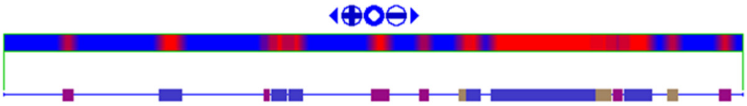

| Name | From | To | Name | From | To | Class | Dir | Sim | Pos/Mm:Ts | Score |
|---|---|---|---|---|---|---|---|---|---|---|
| jockeyFREE | 374 | 436 | DNA-19_AFC | 25 | 85 | DNA | c | 0.7419 | 1.4000 | 202 |
| jockeyFREE | 982 | 1122 | Jockey-15_DEl | 664 | 804 | NonLTR/Jockey | d | 0.7007 | 1.8500 | 353 |
| jockeyFREE | 1640 | 1671 | EnSpm-8_HM | 9521 | 9552 | DNA/EnSpm/CACTA | d | 0.9062 | 3.0000 | 219 |
| jockeyFREE | 1691 | 1783 | NimbX-1_LVa | 1730 | 1820 | NonLTR/Nimb | d | 0.6630 | 1.7647 | 203 |
| jockeyFREE | 1800 | 1882 | Jockey_Ele3 | 1713 | 1795 | NonLTR/Jockey | d | 0.7176 | 1.5833 | 257 |
| jockeyFREE | 2317 | 2425 | SHATAG_MT | 495 | 605 | DNA/hAT | c | 0.7664 | 1.7000 | 244 |
| jockeyFREE | 2617 | 2672 | Mariner-N5B_PM | 263 | 317 | DNA/Mariner | c | 0.7857 | 2.0000 | 210 |
| jockeyFREE | 2863 | 2908 | Gypsy-27_DWil-I | 4947 | 4993 | LTR/Gypsy | d | 0.8085 | 1.4000 | 221 |
| jockeyFREE | 2910 | 2995 | Jockey-5_DAlb | 5064 | 5147 | NonLTR/Jockey | d | 0.7765 | 2.0000 | 351 |
| jockeyFREE | 3069 | 3730 | DOC6_DM | 2879 | 3540 | NonLTR/Jockey | d | 0.6682 | 2.0595 | 604 |
| jockeyFREE | 3731 | 3820 | Gypsy-43_DEu-I | 3645 | 3724 | LTR/Gypsy | d | 0.7882 | 2.5000 | 221 |
| jockeyFREE | 3837 | 3891 | Polinton-1N1_DR | 6044 | 6094 | DNA/Polinton | c | 0.7736 | 1.1111 | 226 |
| jockeyFREE | 3907 | 4077 | Jockey-14_AAe | 3810 | 3980 | NonLTR/Jockey | d | 0.6994 | 2.0000 | 290 |
| jockeyFREE | 4176 | 4238 | COPIA-I_MT | 9167 | 9224 | LTR/Copia | c | 0.7869 | 1.6667 | 210 |
| jockeyFREE | 4500 | 4571 | MuDR-2_GM | 1088 | 1152 | DNA/MuDR | c | 0.7612 | 1.4000 | 226 |

**Figure S5.** Jockey elements in Repbase from *T. castaneum* (**A**) and *T. freemani* (**B**).

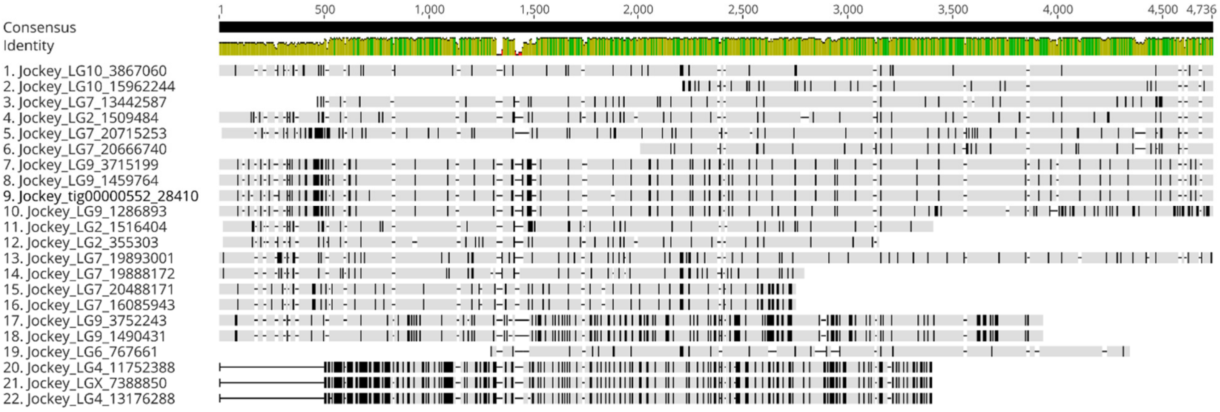


**Figure S6.** Alignment of Jockey elements in *T. castaneum*.
